# Supplementary material for: Enantioselective preparation of mechanically planar chiral rotaxanes by kinetic resolution strategy
Source: Nat Commun. 2021 Jan 15;12:404. doi: 10.1038/s41467-020-20372-0 (PMC7811017; doi:10.1038/s41467-020-20372-0)
Supplement: Supplementary file 1 — Supplementary Information [file 41467_2020_20372_MOESM1_ESM.pdf]

Supplementary Information for

Enantioselective preparation of mechanically planar chiral rotaxanes by kinetic resolution strategy

Ayumi Imayoshi, Bhatraju Vasantha Lakshmi, Yoshihiro Ueda, Tomoyuki Yoshimura, Aki Matayoshi, Takumi Furuta, Takeo Kawabata*

*correspondence to: kawabata@scl.kyoto-u.ac.jp

Table of Contents

1. General Considerations ……………………….……………………….………………………......S2

2. Supplementary Methods ……………………….……………………….…………….…………..….S3

3. Analytical Data ……………………….……………………….…………………..….…………….. S6

4. Computational Details ...……………………….……………………….……………………..….….S12

5. Synthesis of Compounds ……………………….……………………….………….…..…………..S18

6. ^{1}H and ^{13}C NMR Spectroscopy ……………….…………………….………..…...…………..S35

7. Supplementary Reference ….……………………….……………………….……………...….……S66

1. General Considerations

Reagents and solvents: Unless otherwise noted, all commercial reagents and solvents were used as received. Anhydrous toluene, *N*,*N*-dimethylformamide (DMF), tetrahydrofuran (THF), pyridine, dimethylsulfoxide (DMSO), methanol (MeOH), dichloromehthane (CH_2Cl_2) and chloroform ($CHCl_3$) were purchased from commercial suppliers and stored over activated molecular sieves. $CHCl_3$ stabilized with amylene for kinetic resolution of rotaxanes was purchased from Kanto Chemical Co., Inc. and stored over aluminium oxide. Acetic anhydride and 2,4,6-collidine were distilled before use.

Reaction: All reactions, sensitive to air or moisture, were carried out in an argon atmosphere under anhydrous conditions. Thin-layer chromatography (TLC) was performed on Silica gel 60 F_{254} precoated plates (0.25 mm, Merck). Visualization of developed chromatogram was accomplished with UV light and *p*-anysaldehyde (conc. H_2SO_4 in ethanol) or phosphomolybdic acid (5 w/v% in ethanol) stain followed by heating.

Purification: Chromatographic purification of products was accomplished by using Silica gel 60 N (63–210 μm, Kanto Chemical Co., Inc.). Preparative TLC (PTLC) was carried out by using Silica gel 60N F_{254} (0.5 mm, Merck).

Analysis: Analytical HPLC was run on Waters 1525 Binary HPLC Pump, equipped with Waters 2998 Photodiode Array Detector. ^{1}H and ^{13}C NMR spectra were recorded on JEOL ECX-400 (400 and 100 MHz) and JEOL ECA-600 (600 and 150 MHz). Chemical shifts are reported relative to the solvent ($CHCl_3$: δ (^{1}H) = 7.26 ppm, δ (^{13}C) = 77.0 ppm) as reference. Data for ^{1}H NMR are reported as follows: chemical shift (δ ppm), integration, multiplicity (s = singlet, d = doublet, t = triplet, dd = double doublet, ddd = double double doublet, dt = double triplet, m = multiplet, br = broad, brt = broad triplet) and coupling constant (Hz). Data for proton-decoupled ^{13}C NMR are reported in terms of chemical shift. Infrared (IR) spectra were recorded on a JASCO FT-IR 4200 spectrometer and are reported in terms of frequency of absorption (cm^{-1}). High resolution mass spectra (HRMS) were obtained using JEOL JMS-700 mass spectrometer and Bruker Impact HD mass spectrometer. Melting points (m.p.) were recorded using Yanagimoto Micro Melting Point Apparatus PM-500. Specific rotations were measured with JASCO P-2200 polarimeter and HORIBA SEPA-200 automatic digital polarimeter, and are reported as follows: $[\alpha]_D^t$ (*c* = 10 mg/ml, solvent, enantiomeric excess (ee)). UV/Vis absorption spectra were recorded with a JASCO V-550 UV/Vis spectrophotometer. Circular dichroism (CD) spectra were recorded with a JASCO J-720W spectropolarimeter and a JASCO J-820-L spectropolarimeter equipped with a JASCO PTC-423L Peltier Controller.

2. Supplementary Methods

Procedures for preparation of enantiopure mechanically planar chiral rotaxane 1a (Fig. 1c and Table 2, entry 6)

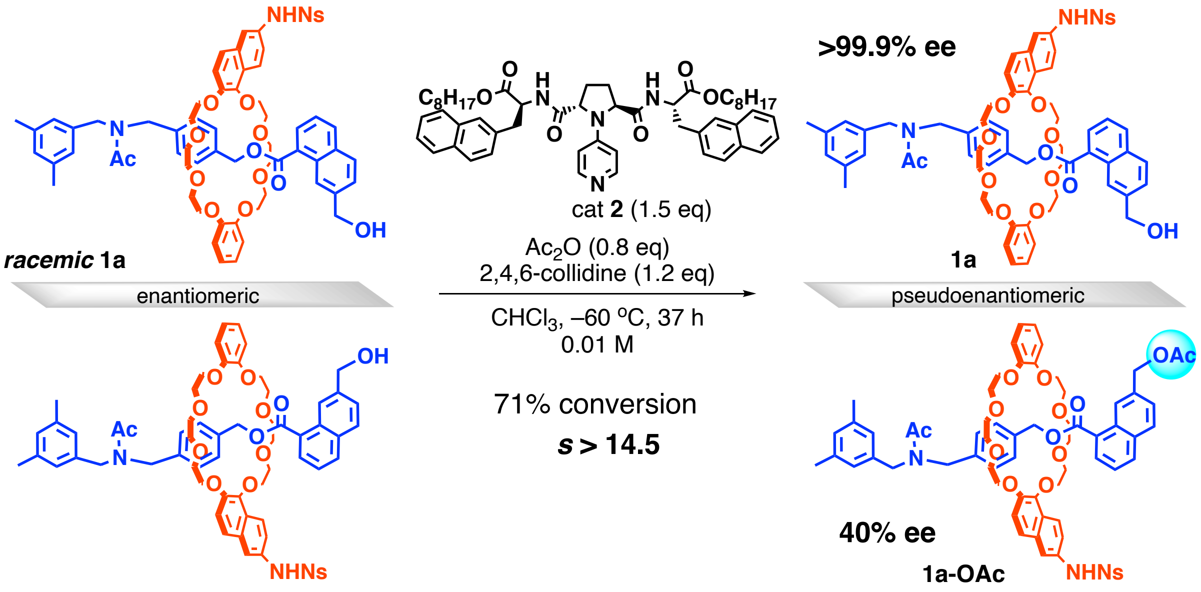

Procedures for preparation of enantiopure mechanically planar chiral rotaxane 1a. To a $CHCl_3$ solution (2.25 ml) of catalyst **2** (54.3 mg, 0.0635 mmol, 1.5 equivalents) were added a $CHCl_3$ solution of 2,4,6-collidine (0.075 M, 0.67 ml, 0.0503 mmol, 1.2 equivalents) and a $CHCl_3$ solution (1.0 ml) of *racemic* rotaxane **1a** (50.0 mg, 0.0424 mmol, 1.0 equivalents) at room temperature. The mixture was cooled to –60 °C. A $CHCl_3$ solution of acetic anhydride (0.105 M, 0.32 ml, 0.0336 mmol, 0.8 equivalents) was added dropwise to the mixture, and the reaction mixture was stirred at –60 °C for 37 h. Then MeOH (15 ml) was added to the reaction mixture at –60 °C, and the mixture was stirred at room temperature for 30 min. The mixture was evaporated to dryness under a reduced pressure. The crude product was subjected to preparative TLC (SiO_2, MeOH/$CHCl_3$ 1/19) to allow isolation of recovered **1a** (15.0 mg, 29% yield) and acylated **1a-OAc** (33.0 mg, 65% yield). Enantiomeric purity of the recovered rotaxane, **1a** (>99.9% ee), and the acylated product, **1a-OAc** (40% ee), was determined by chiral HPLC with CHIRALPAK-IC column eluted by EtOH/CH_2Cl_2 1/60 (0.7 ml/min) at 20 °C. Conversion (C, C = 71%) and selectivity factor (*s*, *s* >14.5) were determined according to the following equation: conversion $C = ee_{1a} / (ee_{1a} + ee_{1a\text{-}OAc})$ and selectivity factor $s = \ln[(1-C)(1-ee_{1a})]/\ln[(1-C)(1+ee_{1a})] = \ln[1-C(1+ee_{1a\text{-}OAc})]/\ln[1-C(1-ee_{1a\text{-}OAc})]$.

General procedures for kinetic resolution of *racemic* rotaxane 1a on an analytical scale (Table 2, entry 5)

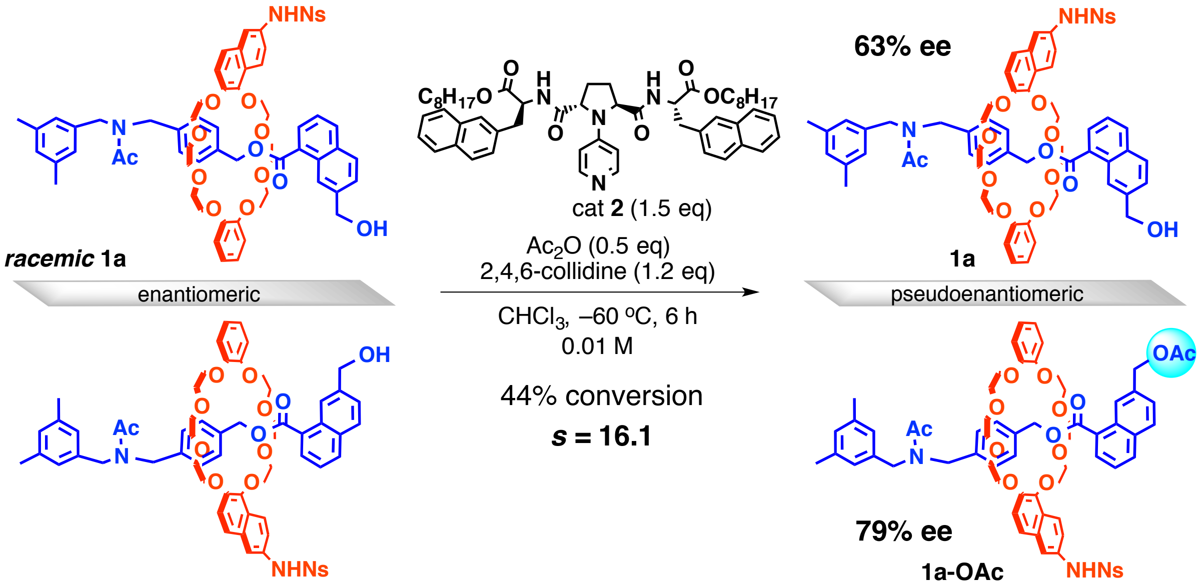

To a $CHCl_3$ solution (15 μl) of catalyst **2** (1.1 mg, 1.3 μmol, 1.5 equivalents), were added $CHCl_3$ (31 μl), a $CHCl_3$ solution of 2,4,6-collidine (0.075 M, 13.4 μl, 1.0 μmol, 1.2 equivalents) and a $CHCl_3$ solution (20 μl) of rotaxane **1a** (1.0 mg, 0.85 μmol, 1.0 equivalents) at room temperature. The mixture was cooled to –60 °C. A $CHCl_3$ solution of acetic anhydride (0.105 M, 4.0 μl, 0.42 μmol, 0.5 equivalents) was added to the mixture and the reaction mixture was kept at –60 °C for 6 h. Then MeOH (300 μl) was added to the reaction mixture at –60 °C, and the mixture was kept at room temperature for 30 min. The mixture was evaporated to dryness under a reduced pressure. The resulting products were analyzed by chiral stationary phase HPLC with CHIRALPAK-IC column eluted by EtOH/CH_2Cl_2=1/60 (0.7 ml/min) at 20 °C to determine the enantiomeric purity of the recovered rotaxane, **1a** (63% ee), and the acylated product, **1a-OAc** (79% ee). Conversion (C, C = 44%) and selectivity factor (*s*, *s* = 16.1) were determined according to the following equation: conversion $C = ee_{1a} / (ee_{1a} + ee_{1a\text{-}OAc})$ and selectivity factor $s = \ln[(1–C)(1–ee_{1a})]/\ln[(1–C)(1+ee_{1a})] = \ln[1–C(1+ee_{1a\text{-}OAc})]/\ln[1–C(1–ee_{1a\text{-}OAc})]$.

catalyst **2**
acylating agent
base

***racemic* 1** → (solvent, –60 ºC) **enantioenriched 1** + **enantioenriched 1-OAc**

Entry	rotaxane	concentration (M)	catalyst **2** (equiv)	acylating agents (equiv)	base (equiv)	solvent	*s*
1	Me (**1a**)	0.01	1.5	Ac_2O (0.5)	2,4,6-collidine (1.2)	$CHCl_3$	16.1
2	Me (**1a**)	0.001	1.5	Ac_2O (0.5)	2,4,6-collidine (1.2)	$CHCl_3$	4.4
3	Me (**1a**)	0.1	1.5	Ac_2O (0.5)	2,4,6-collidine (1.2)	$CHCl_3$	2.6
4	Me (**1a**)	0.006	1.5	Ac_2O (0.5)	2,4,6-collidine (1.7)	$CHCl_3$	6.3
5	Me (**1a**)	0.006	1.5	Ac_2O (0.5)	2,4,6-collidine (1.2)	$CHCl_3$	6.6
6	Me (**1a**)	0.006	1.5	Ac_2O (0.5)	2,4,6-collidine (0.9)	$CHCl_3$	6.3
7	Me (**1a**)	0.01	1.5	Ac_2O (0.5)	triethylamine (1.2)	$CHCl_3$	– *
8	Ad (**1c**)	0.07	0.5	Ac_2O (0.9)	2,4,6-collidine (1.7)	$CHCl_3$	1.9
9	Ad (**1c**)	0.07	0.5	$(^{i}PrCO)_2O$ (0.9)	2,4,6-collidine (1.7)	$CHCl_3$	1.8
10	Me (**1a**)	0.005	0.5	Ac_2O (0.5)	2,4,6-collidine (1.7)	toluene/$CHCl_3$ (20/1)	1.6
11	Me (**1a**)	0.005	0.5	Ac_2O (0.5)	2,4,6-collidine (1.7)	CS_2/$CHCl_3$ (20/1)	1.2

* By-products were observed, and the selectivity factor (*s*) was low.

Supplementary Table 1. Investigation of reaction parameters.

3. Analytical Data

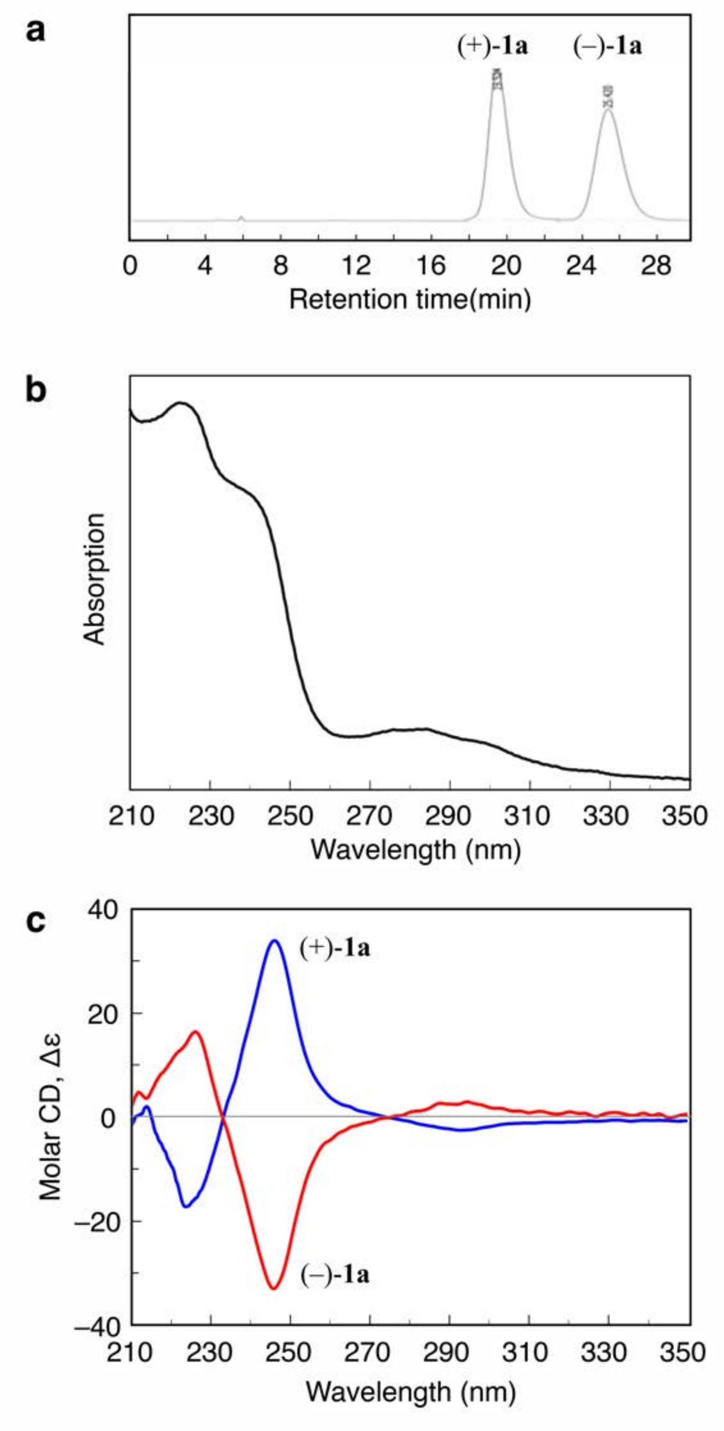

Supplementary Figure 1. Analytical data of rotaxane **1a**. (**a**) Chiral HPLC chromatogram of *racemic* **1a**. Conditions for HPLC: CHIRALPAK IC column (4.6×250 mm); eluent EtOH/CH_2Cl_2 1/60; flow rate 0.7 ml/min; detection 254 nm, temperature 20 °C. (**b**) UV/Vis spectrum of **1a**. Recorded in 0.5×10^{-5} M MeOH. (**c**) CD spectra of a pair of enantiomers of rotaxane **1a**. Recorded in 0.5×10^{-5} M MeOH.

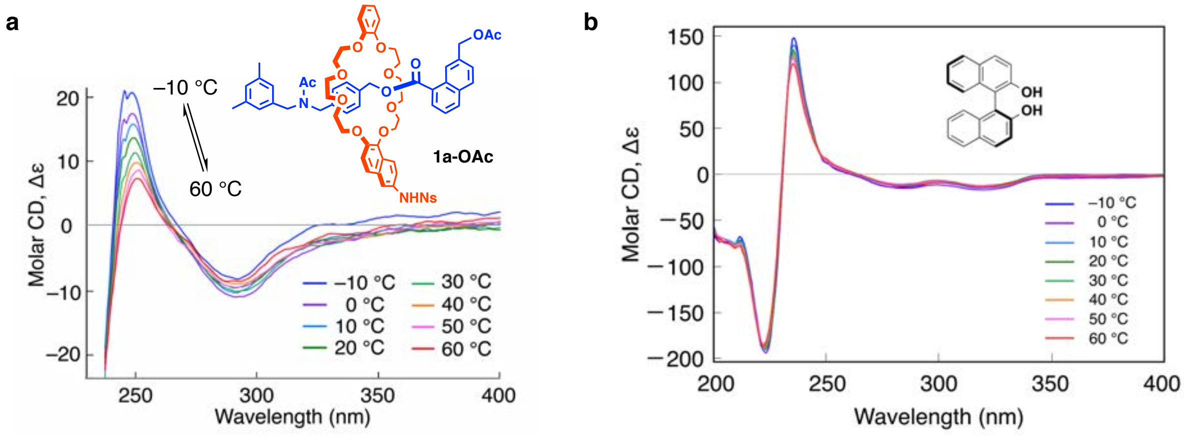

Supplementary Figure 2. Comparison of the temperature-dependency of CD spectra of enantiopure (+)-**1a-OAc** and (*S*)-(–)-1,1'-bi-2-naphthol. (**a**) Unusually strong temperature-dependency of the CD spectra of enantiopure (+)-**1a-OAc**. Recorded by heating a 1.38×10^{-5} M MeCN solution of (+)-**1a-OAc**. The temperature-dependent CD behavior was reversible. The tentative absolute configuration of **1a-OAc** is shown. (**b**) For comparison, Temperature-independent CD spectra of (*S*)-(–)-1,1'-bi-2-naphthol, a typical CD active chiral molecule. The small decrease in molar CD with increasing temperature seems to be caused by thermal expansion of the volume of the solvent. Conditions: Recorded by heating a 0.5×10^{-5} M MeCN solution of (*S*)-(–)-1,1'-bi-2-naphthol from –10 to 60 °C.

a

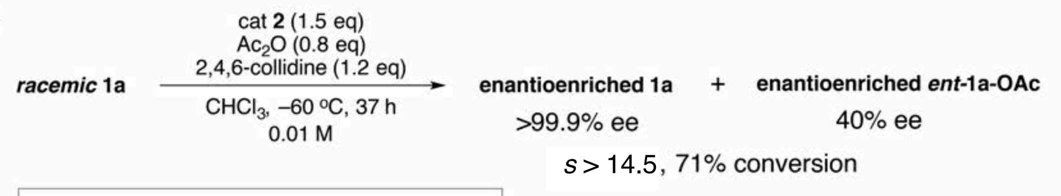

b

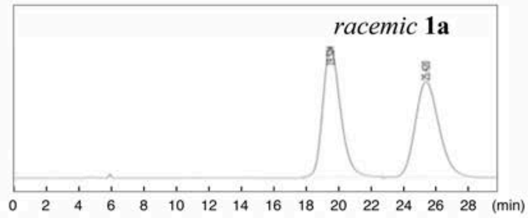

Retention time (min)	Area	% Area
19.524	3765444	50.34
25.420	3714530	49.66

c

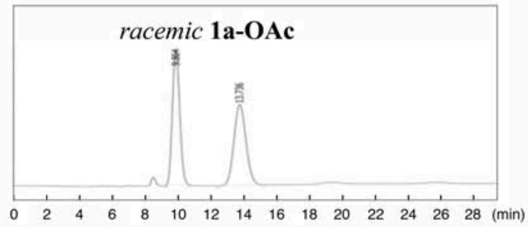

Retention time (min)	Area	% Area
9.864	6906524	50.37
13.736	6806010	49.63

d

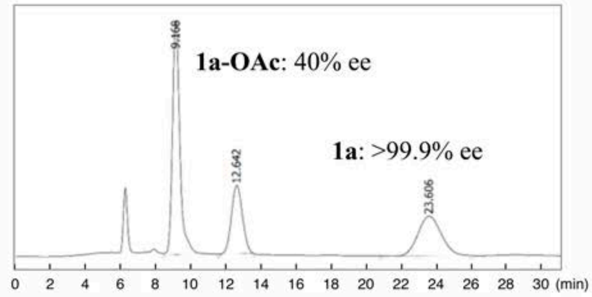

Retention time (min)	Area	% Area
9.168	11240033	51.09
12.642	4831686	21.96
23.606	5928000	26.95

e

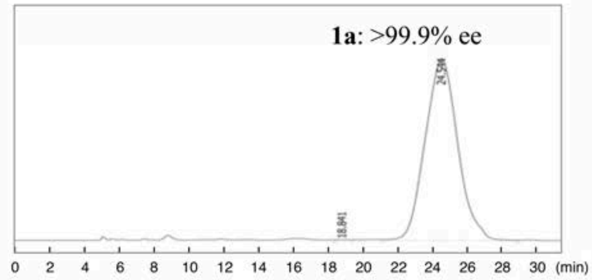

Retention time (min)	Area	% Area
18.841	10026	0.01
24.544	67024334	99.99

f

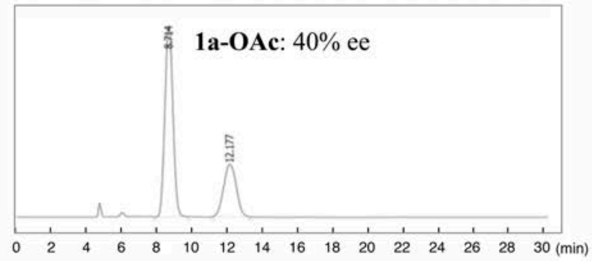

Retention time (min)	Area	% Area
8.714	13329617	69.98
12.177	5719404	30.02

Supplementary Figure 3. Analysis of kinetic resolution of *racemic* **1a** shown in Fig. 1c and Table 2, entry 6. (**a**) Reaction scheme. Chiral HPLC chromatograms of (**b**) *racemic* **1a**, (**c**) *racemic* **1a-OAc**, (**d**) crude product of the kinetic resolution, (**e**) isolated enantioenriched **1a**, and (**f**) isolated enantioenriched **1a-OAc**. Conditions for HPLC: CHIRALPAK IC column (4.6×250 mm); eluent 1/60 EtOH/CH_2Cl_2; flow rate 0.7 ml/min; detection 254 nm, temperature 20 °C.

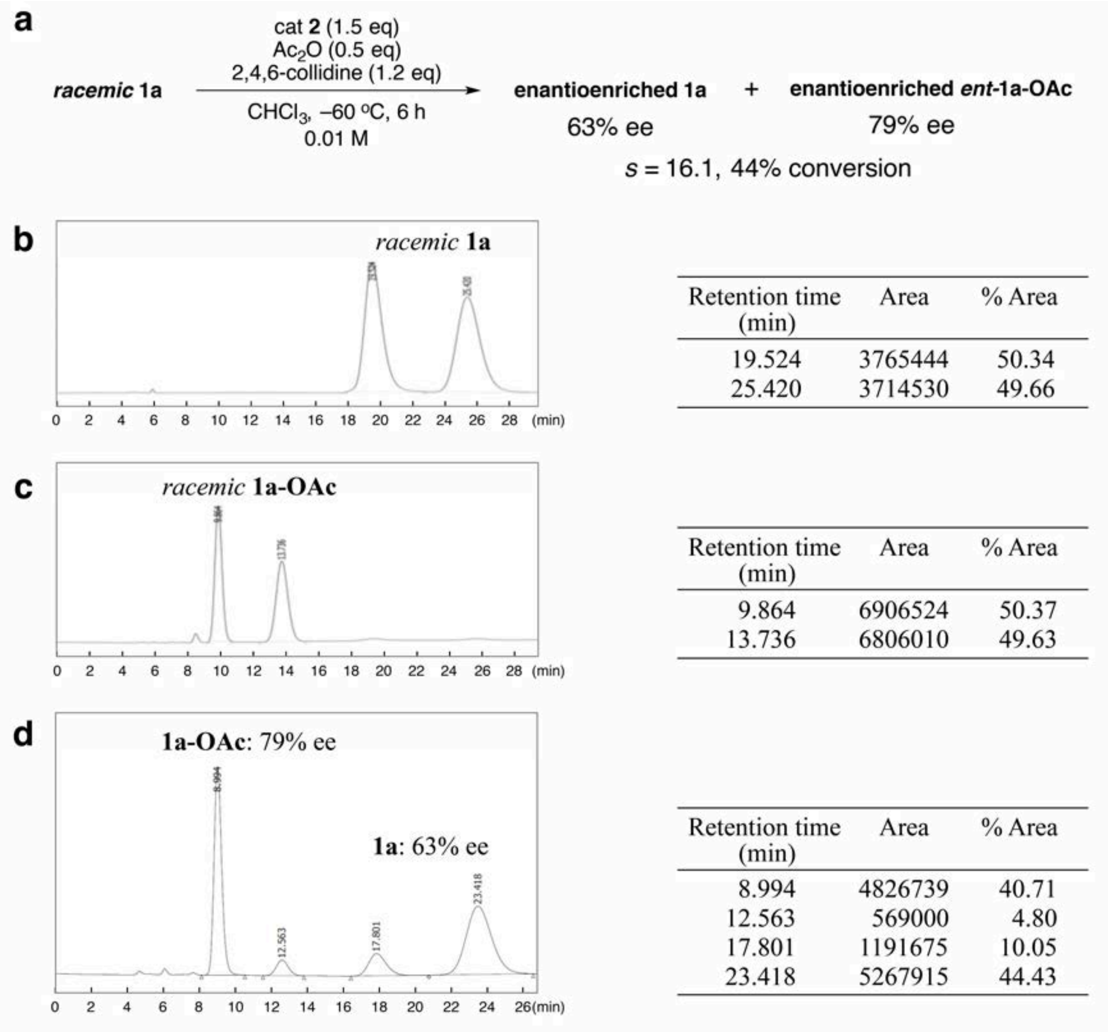

b

Retention time (min)	Area	% Area
19.524	3765444	50.34
25.420	3714530	49.66

c

Retention time (min)	Area	% Area
9.864	6906524	50.37
13.736	6806010	49.63

d

Retention time (min)	Area	% Area
8.994	4826739	40.71
12.563	569000	4.80
17.801	1191675	10.05
23.418	5267915	44.43

Supplementary Figure 4. Analysis of kinetic resolution of *racemic* **1a** shown in Table 2, entry 5. (**a**) Reaction scheme. Chiral HPLC chromatograms of (**b**) *racemic* **1a**, (**c**) *racemic* **1a-OAc**, and (**d**) crude product of the kinetic resolution. Conditions for HPLC: CHIRALPAK IC column (4.6×250 mm); eluent EtOH/CH_2Cl_2 1/60; flow rate 0.7 ml/min; detection 254 nm, temperature 20 °C.

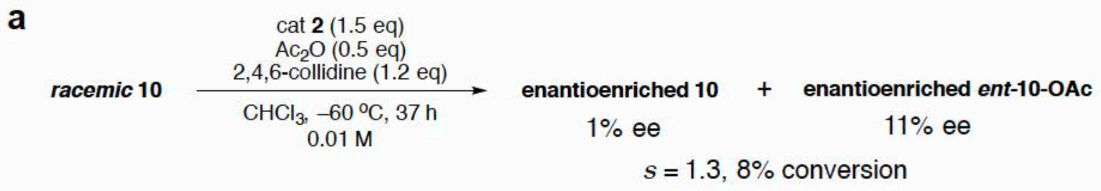

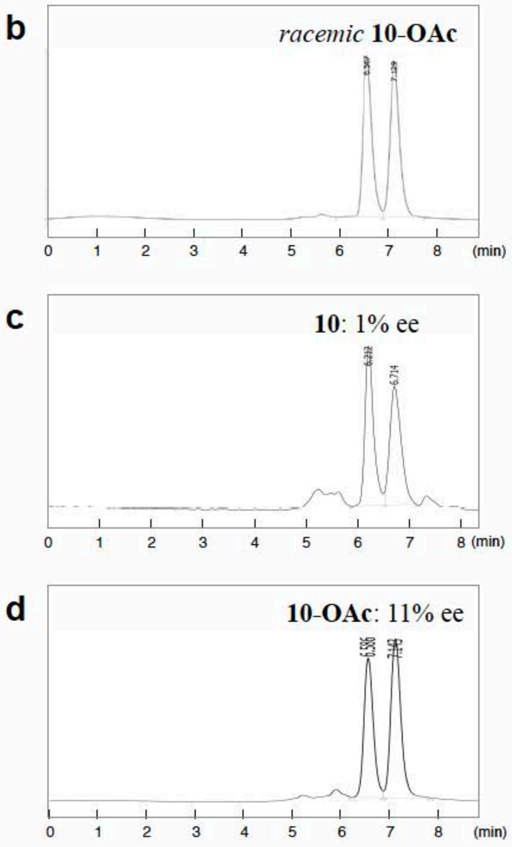

b

Retention time (min)	Area	% Area
6.567	15496078	49.37
7.129	15888699	50.63

c

Retention time (min)	Area	% Area
6.212	94705	50.68
6.714	92180	49.32

d

Retention time (min)	Area	% Area
6.586	2505506	44.72
7.413	3097666	55.28

Supplementary Figure 5. Analysis of kinetic resolution of *racemic* **10**. (**a**) Reaction scheme. Chiral HPLC chromatograms of (**b**) *racemic* **10-OAc**, (**c**) enantioenriched **10** (ee of **10** was determined after converting to **10-OAc**), and (**d**) enantioenriched **10-OAc**. Conditions for HPLC: CHIRALPAK ID column (4.6×250 mm); eluent EtOH/$CHCl_3$ 1/540; flow rate 0.7 ml/min; temperature 20 °C.

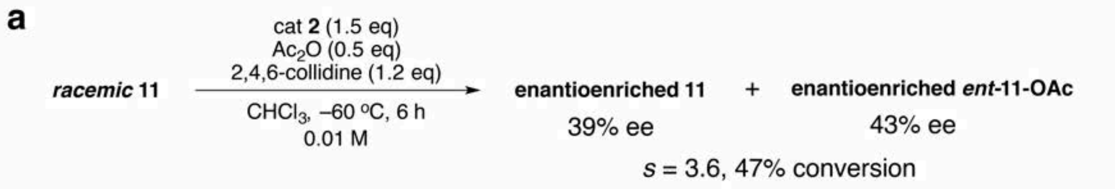

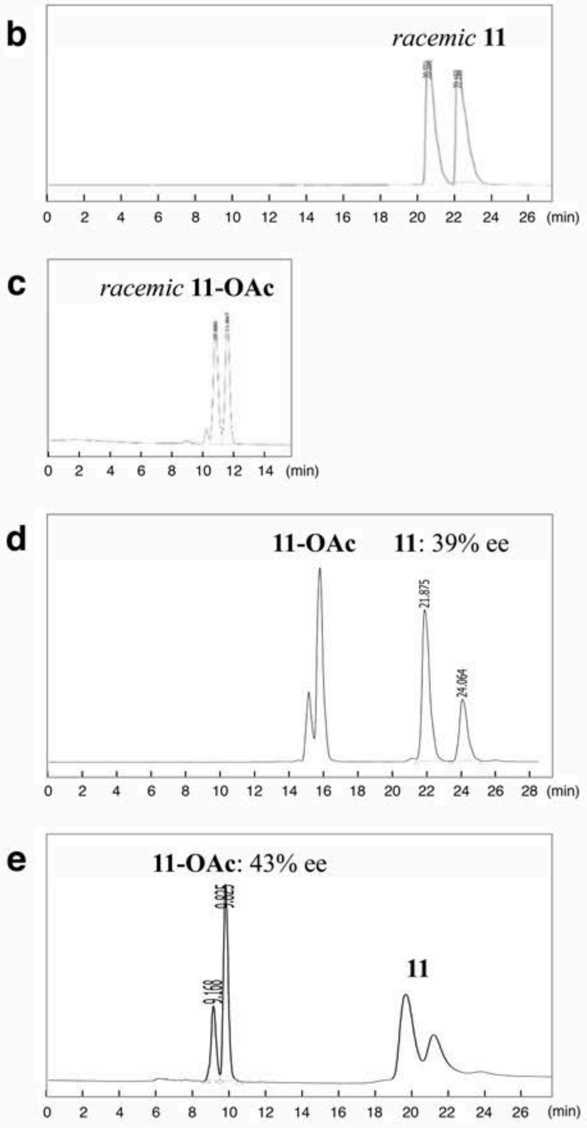

Retention time (min)	Area	% Area
20.551	108720926	49.17
22.193	112404403	50.83

Retention time (min)	Area	% Area
10.850	5923032	50.26
11.617	5862701	49.74

Retention time (min)	Area	% Area
21.875	30492692	69.54
24.064	13354707	30.46

Retention time (min)	Area	% Area
9.168	686190	28.46
9.825	1725002	71.54

Supplementary Figure 6. Analysis of kinetic resolution of *racemic* **11**. (**a**) Reaction scheme. Chiral HPLC chromatograms of (**b**) *racemic* **11**, (**c**) *racemic* **11-OAc**, and (**d**, **e**) crude product of the kinetic resolution. Conditions for HPLC (**b**, **d**) : CHIRALPAK ID column (4.6×250 mm); eluent EtOH/CH_2Cl_2 1/40; flow rate 0.5 ml/min; temperature 20 °C. Conditions for HPLC (**c**, **e**): CHIRALPAK ID column (4.6×250 mm); eluent EtOH/CH_2Cl_2 1/80; flow rate 1.0 ml/min; temperature 20 °C.

4. Computational Details

A model for the transition state assembly was generated by molecular mechanics (MacroModel in Material Science Suite 2019-4) and DFT calculation (gaussian 16). The conformational search for the combined structure (**I** + **II**) (Supplementary Figure 7a) by molecular mechanics simulation with the constraint of (2.7 ± 0.3) Å for the distance between C^{I} and O^{II} atoms and the constraint of (2.1 ± 0.3) Å for the distance between O^{I} and H^{II} atoms. Geometry optimization of the most stable conformer (**I** + **II**) with acetate anion was conducted by ONIOM(M06-2X/6-31+G**:PM6) method (Supplementary Figure 7b). The optimization of the transition state structure **A** was performed at the same level of theory and characterized by frequency calculation (only one imaginary frequency). Note that the obtained transition state structure **A** is one of the possible transition state structures, and it does not mean the most stable one. Computational time was generously provided by the Supercomputer Laboratory in the Institute for Chemical Research of Kyoto University.

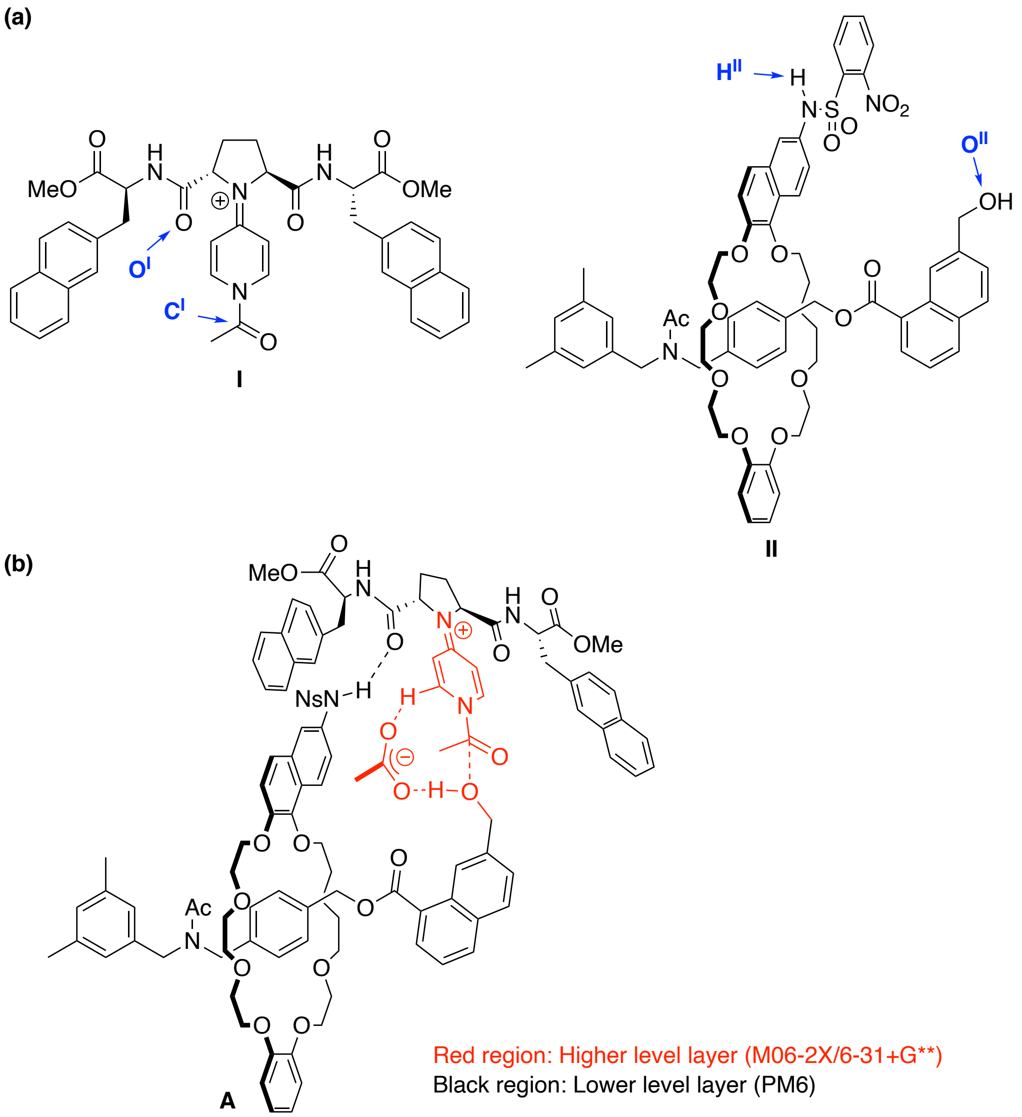

Supplementary Figure 7. Chemical Models for Calculations. (a) Acylpyridinium Cation **I** and Substrate **II**. (b) ONIOM Partitioning Scheme for TS Calculation.

XYZ coordinates

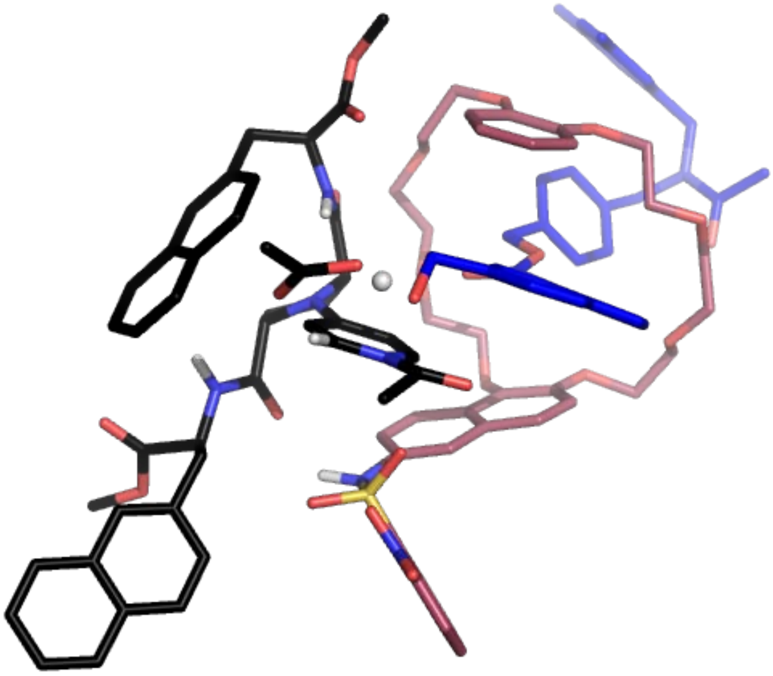

N	-3.07388900	-0.01650800	2.36908100
C	-3.77573700	0.99516800	1.82731700
C	-3.44249400	1.49088100	0.58798200
C	-2.32549900	0.97923400	-0.09547600
C	-1.62109200	-0.06970000	0.49525300
C	-2.03560900	-0.56685900	1.71984700
N	-1.91429600	1.61392600	-1.27597200
C	-0.50336200	1.54120200	-1.72739900
C	-0.57050800	1.11884000	-3.20548800
C	-1.92842900	1.64314000	-3.70103500
C	-2.82501000	1.76969800	-2.44033300
C	0.10771700	2.96123900	-1.61989600
C	-3.93740200	0.69603600	-2.50271000
O	0.94145900	3.37160400	-2.41834300
O	-3.74062400	-0.45485800	-2.86763400
N	-5.20972700	1.14513400	-2.17814300
N	-0.28401500	3.75792300	-0.54477700
C	0.06212900	5.18993700	-0.51171000
C	0.59486600	5.51679700	0.89064200
O	1.68989000	6.33313400	0.73019600
O	0.18463100	5.18902900	1.97561300
C	-6.39507100	0.26469200	-2.27766300
C	-7.27241200	0.83128400	-3.40809100
O	-7.70229600	1.95161300	-3.55085700
O	-7.50290600	-0.17404900	-4.30706800
C	-8.40074300	0.11439100	-5.41443700
C	2.32390900	6.83975700	1.93829800
C	-1.13213800	6.11760900	-0.83394700
C	-7.18470700	0.24449700	-0.95642700
C	-2.47878300	5.58510700	-0.41949000
C	-8.52544500	-0.41803700	-1.12057600
C	-8.61950300	-1.83501300	-0.94818000
C	-9.82459200	-2.47207400	-1.08762100

C	-11.00783300	-1.73210500	-1.40822900
C	-10.91607000	-0.32629900	-1.57987000
C	-9.64792000	0.31651800	-1.42951900
C	-12.27850000	-2.37240200	-1.55933400
C	-13.39555700	-1.63672200	-1.86753200
C	-13.30359300	-0.22448300	-2.04073500
C	-12.09769000	0.41561000	-1.90120400
C	-3.45071500	5.34893800	-1.36699000
C	-4.73382400	4.84788200	-0.98284300
C	-4.99249500	4.58435200	0.38701600
C	-3.96623700	4.83883900	1.35168900
C	-2.74835600	5.33071300	0.96173300
C	-5.76186700	4.58803400	-1.94403000
C	-6.97885900	4.09442800	-1.54194800
C	-7.23479500	3.82761800	-0.16479600
C	-6.26548200	4.06102200	0.77789700
C	-3.34389900	-0.53078300	3.80661900
O	-2.60828200	-1.42137500	4.16988900
C	-4.78658500	-0.41295600	4.22225800
H	-4.53752000	1.46320500	2.43979600
H	-3.99040900	2.33721300	0.19570200
H	-0.74810600	-0.51251000	0.01412400
H	-1.56146100	-1.39493600	2.23911000
H	0.13407500	0.84300000	-1.10887200
H	0.28056800	1.52170800	-3.79004700
H	-0.51096300	0.01593200	-3.28848200
H	-1.81652000	2.62639800	-4.19194500
H	-2.36673500	0.95966800	-4.45140800
H	-3.27251500	2.80004800	-2.37602300
H	-5.38510900	2.09750800	-1.83718000
H	-1.02978400	3.46449400	0.09459800
H	0.88557200	5.38591900	-1.26660500
H	-6.07325100	-0.78894000	-2.53713300
H	-8.20996400	1.11532500	-5.81502000
H	-8.15666000	-0.67187100	-6.13599700
H	-9.42878200	0.02658000	-5.04738600
H	2.82178100	6.01198100	2.45723800
H	3.04094100	7.56712600	1.54418700
H	1.57575600	7.30871100	2.58489100
H	-0.97299600	7.11460700	-0.36600400
H	-1.12165200	6.31698600	-1.92845500
H	-6.57865100	-0.31675700	-0.19853100
H	-7.30251200	1.26900900	-0.54541600
H	-7.70560500	-2.38715600	-0.70068500
H	-9.90399500	-3.54961600	-0.95613200
H	-9.59032900	1.39598600	-1.57093200
H	-12.33912300	-3.45065100	-1.42317600
H	-14.36621900	-2.11531700	-1.98276600
H	-14.20709500	0.33231100	-2.28300800
H	-12.01765200	1.49401500	-2.02947600
H	-3.26917400	5.54993800	-2.42053600
H	-4.18086400	4.61291200	2.40405900
H	-1.97025100	5.52560800	1.70465600
H	-5.57068100	4.80655900	-2.99282400
H	-7.76269500	3.88098400	-2.27584800
H	-8.21375200	3.44628900	0.12269600
H	-6.43004200	3.84424200	1.84316500
H	-4.83264300	-0.68377700	5.27485700

H	-5.21586800	0.57552600	4.07464400
H	-5.35348200	-1.15478500	3.63977700
C	9.13480400	-1.70474100	-2.37269200
N	10.09643200	-1.76553300	-1.23178900
C	11.18967600	-0.76075400	-1.23357500
C	5.19576400	-0.44038500	-1.11654700
C	5.54114000	-1.79754100	-1.12751600
C	6.81201200	-2.18991100	-1.54659300
C	7.76247500	-1.23965300	-1.94018200
C	7.42032300	0.11855500	-1.92109400
C	6.14898700	0.51523600	-1.50018200
C	3.80228600	-0.02090100	-0.75112900
C	9.80794200	3.32115500	-1.28892000
C	10.59998800	2.83185400	-2.33792100
C	11.03858000	1.50033400	-2.31893800
C	10.69070200	0.66863100	-1.24535600
C	9.91260200	1.15705900	-0.19363000
C	9.45618600	2.48806400	-0.21724400
C	10.96154300	3.73359200	-3.47437800
C	8.57950100	2.99465200	0.87368300
O	3.51584700	-0.68831100	0.52499200
C	2.25130900	-0.56364300	1.04769500
O	1.38687300	0.05505400	0.45545100
C	2.23275300	-2.72926200	4.75625300
C	2.19427700	-1.29471700	2.33665500
C	3.00195200	-2.40833200	2.48629200
C	3.02590100	-3.12767200	3.70667100
C	1.36533700	-1.60444400	4.62345400
C	1.33340800	-0.86866700	3.40495600
C	0.42166200	0.22776400	3.28969600
C	-0.44710800	0.52940100	4.31261600
C	-0.39411400	-0.19712400	5.54436300
C	0.49476900	-1.22560900	5.69803400
C	-1.51095500	1.60675600	4.16497800
O	-2.77274800	1.14153000	4.54149600
H	9.54445100	-1.03290600	-3.15963600
H	9.06895600	-2.72732900	-2.81866700
H	11.85676700	-0.90157200	-0.35243400
H	11.83103100	-0.95958400	-2.12715700
C	9.87645700	-2.67421400	-0.19555300
H	7.07948300	-3.25614600	-1.53477400
H	8.14828300	0.87504800	-2.21712900
H	9.46016600	4.35073000	-1.30933700
H	11.64513000	1.11926300	-3.13467000
H	9.62834000	0.50354200	0.62661400
H	11.46505100	4.64331200	-3.11720300
H	11.63210100	3.25831900	-4.20094900
H	10.06430600	4.05491600	-4.02351200
H	7.52659100	2.66310300	0.70930300
H	8.55371000	4.08783200	0.93866100
H	8.86257300	2.61200500	1.86140800
H	4.81856300	-2.55286900	-0.79693400
H	5.89984900	1.58099100	-1.46591800
H	3.04989100	-0.34817100	-1.50031500
H	3.69662100	1.07339500	-0.57969700
H	2.24645500	-3.27135300	5.70251000
H	3.64344900	-2.74809600	1.65937400
H	3.69189400	-3.98821500	3.79156400

H	0.41612800	0.79946300	2.35755300
H	-1.10887900	0.07015900	6.32628400
H	0.53760800	-1.79144800	6.62740200
H	-1.51898400	1.98433900	3.11562300
H	-1.23145900	2.46878500	4.80159100
O	8.98545700	-3.51447900	-0.28956100
C	10.77798800	-2.62579200	1.00784300
H	10.51578400	-3.44222100	1.70086700
H	10.66880300	-1.68601300	1.56682000
H	11.83704800	-2.75537800	0.75327200
C	2.34523700	-4.46561400	-0.08215300
C	2.87671600	-4.23016000	-1.38379800
C	2.07733400	-3.66581800	-2.37423400
C	0.69673000	-3.38935100	-2.11251700
C	0.15729000	-3.69959000	-0.83723600
C	1.02450100	-4.20944400	0.18135600
C	-0.16150800	-2.83070800	-3.11276800
C	-1.49163600	-2.63706800	-2.87024600
C	-2.04925700	-3.01500200	-1.59748700
C	-1.23698500	-3.50281900	-0.59327000
O	4.19311200	-4.50228600	-1.71661500
O	2.52997700	-3.46620900	-3.67188000
C	4.81986600	-5.62859500	-1.03488200
C	5.90065300	-5.14489100	-0.07238700
O	5.21260400	-4.57689200	1.06982000
C	6.07426600	-4.29438100	2.19253600
C	7.02805100	-3.12339500	1.93160500
O	6.80855700	-2.11130100	2.93312800
C	5.93061100	-1.02978600	2.55272000
C	6.78881500	0.23845800	2.60057300
O	6.23141800	1.29595500	1.77514800
C	3.40777000	-2.31786900	-3.86290000
C	2.57229100	-1.19200400	-4.48268100
O	2.73051200	0.04463600	-3.76715800
C	3.88827000	0.83532600	-4.15528600
C	3.47989600	2.28911100	-3.90685000
O	4.40760100	2.86038300	-2.95513400
C	3.78524400	3.32041800	-1.72777900
C	4.92668100	3.93341800	-0.92168100
O	5.37996600	3.04735500	0.13758000
C	5.04256700	1.87657500	2.15783900
C	4.55363300	2.85065100	1.22942200
C	4.32489400	1.60498500	3.31718400
C	3.10855900	2.27142800	3.55113100
C	2.62721200	3.20383100	2.64214000
C	3.35315000	3.50429400	1.47460400
N	-3.46761300	-2.92635600	-1.47612600
S	-4.32069300	-2.95747000	0.01911700
O	-3.44844800	-2.42488900	1.04070000
O	-5.57900700	-2.32890000	-0.33212600
C	-4.57462800	-4.66671500	0.34664500
C	-5.24383600	-5.07058900	1.51435600
C	-5.44336300	-6.41567400	1.82313200
C	-4.96508800	-7.39337300	0.93632700
C	-4.30540800	-7.01074200	-0.22824400
C	-4.11005700	-5.64773200	-0.52213300
N	-5.76660400	-4.04050100	2.46234900
O	-6.30973100	-4.41787900	3.48000100

O	-5.61039000	-2.87551300	2.12213200
H	3.02160400	-4.83234100	0.69912600
H	0.61613500	-4.38239700	1.17804100
H	0.29144400	-2.56069800	-4.07509600
H	-2.14823900	-2.18470800	-3.61731200
H	-1.61165200	-3.72049900	0.40651700
H	5.27242200	-6.17760500	-1.88595000
H	4.07123300	-6.26529700	-0.53527700
H	6.55090800	-4.36193900	-0.52710700
H	6.53605200	-5.98597000	0.26042700
H	6.61302500	-5.21274100	2.47829200
H	5.33647900	-4.03848600	2.98380000
H	8.08655400	-3.40211700	2.09106100
H	6.92422100	-2.71611700	0.90903900
H	5.13239700	-1.03793200	3.31754200
H	5.47477900	-1.17142200	1.55124000
H	6.95089300	0.61190300	3.62324000
H	7.76310200	0.08236800	2.09552500
H	4.17129200	-2.72430200	-4.54887200
H	3.88611000	-2.01278900	-2.91691100
H	2.78817100	-1.04929100	-5.55373700
H	1.48563900	-1.40311600	-4.35625800
H	4.14448100	0.64774600	-5.20933800
H	4.73298300	0.53167800	-3.50912100
H	3.60502900	2.91535100	-4.80749200
H	2.42732300	2.35588900	-3.55551000
H	2.99323300	4.05050900	-1.98237900
H	3.33066700	2.44863500	-1.20556700
H	5.85463500	4.00655100	-1.53158900
H	4.67343000	4.91781800	-0.49754200
H	4.68061500	0.87606100	4.04168000
H	2.53984700	2.03571400	4.45092200
H	1.67386800	3.70663600	2.82232300
H	-3.90781600	-2.34946000	-2.23606200
H	-5.96346100	-6.70080200	2.74570200
H	-5.11360300	-8.45074500	1.16840800
H	-3.93036100	-7.76700000	-0.92231600
H	-3.58075500	-5.38324200	-1.44786600
H	2.96284100	4.23919000	0.77408900
H	-3.58293800	2.02958600	5.04287600
C	-5.13010600	3.32464600	4.77445100
C	-5.98130100	4.36659900	5.46657300
H	-6.67937800	4.81731300	4.76295700
H	-6.53089300	3.89169800	6.28425400
H	-5.33855300	5.13037200	5.91086000
O	-5.30630300	3.04086200	3.58548200
O	-4.23468900	2.78662700	5.54249800

5. Synthesis of Compounds

Purification of catalyst 2

Catalyst **2** was prepared according to the reported method[1], then further purification was performed in the following way. After extraction and evaporation of the reaction mixture, the residue was subjected to column chromatography (SiO_2, AcOEt/Hexane 2/1 to MeOH/$CHCl_3$ 1/99 to 1/50), affording a pale yellow residue containing **2**. The pale yellow residue was dissolved in MeOH, and the mixture was evaporated to dryness under a reduced pressure. Then the resulting residue was dissolved in AcOEt, and the mixture was evaporated to dryness under a reduced pressure. The pale yellow residue was recrystallized from hot AcOEt by cooling and adding a small amount of hexane, affording a slightly pale yellow residue. To the slightly pale yellow residue was added a small amount of MeOH, which was filtered to afford completely pure catalyst **2** (7.02 g) as white solid.

Synthesis of ring component 14

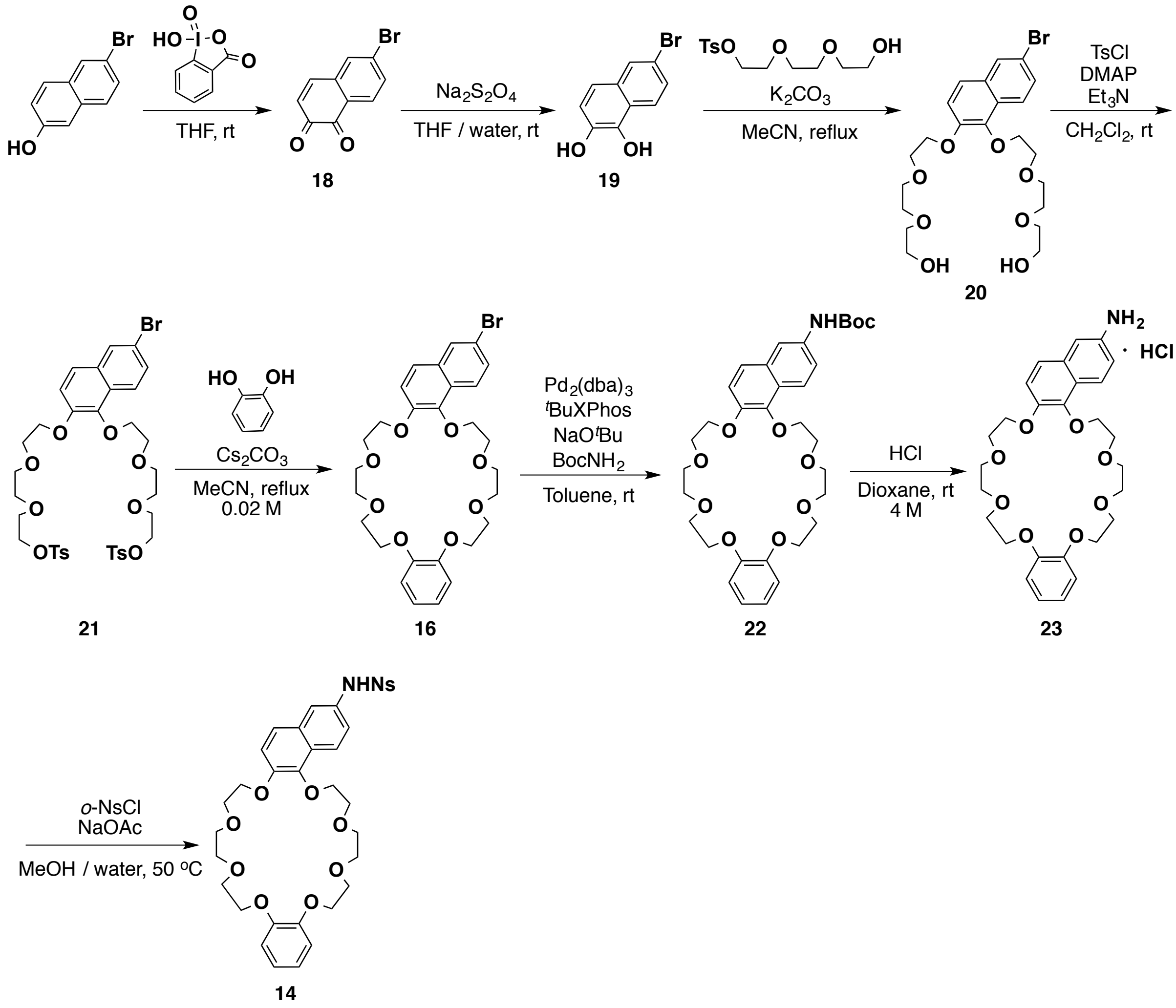

6-Bromonaphthalene-1,2-dione (18): To a THF solution (960 ml) of 6-bromo-2-naphthol (35.6 g, 160 mmol) was added 2-iodoxybenzoic acid (50 g, 178 mmol) at room temperature, which changed its color to bright orange within 1 h. The reaction mixture was stirred at room temperature for 16 h in the dark. The reaction mixture was evaporated to dryness under a reduced pressure. The residue was diluted with $CHCl_3$. The mixture was washed successively with saturated aqueous solution of $NaHCO_3$ and brine, and an organic phase separated was dried over Na_2SO_4, filtered and evaporated to dryness under a reduced pressure. The residue was obtained as bright orange solid, which was directly used for the next step.

6-Bromonaphthalene-1,2-diol (19): The following steps were carried out under Ar atmosphere. All solvents and reaction vessels were degassed by flushing with Ar gas. The bright orange solid containing 6-bromonaphthalene-1,2-dione (**18**) was dissolved in degassed THF (245 ml) at room temperature under Ar. To the solution was added a degassed aqueous solution (222 ml) of $Na_2S_2O_4$ (145 g, 835 mmol), then the mixture changed its color to pale orange within a few minutes. The reaction mixture was stirred under Ar atmosphere at room temperature for 15 min. The mixture was evaporated to dryness under a reduced pressure under Ar atmosphere. The residue was directly used for the next step.

Compound 20: The following steps were carried out under Ar atmosphere. All solvents and reaction vessels were degassed by flushing with Ar gas. To a degassed MeCN solution (50 ml) of a mixture of triethylene glycol monotosylate (52 g, 171 mmol) and powdered K_2CO_3 (66.4 g, 480 mmol) was added a degassed MeCN solution (300 ml) of 6-bromonaphthalene-1,2-diol (**19**) at reflux temperature under Ar atmosphere. The reaction mixture was refluxed under Ar atmosphere for 37 h. The reaction mixture was cooled to room temperature, filtered through a celite pad, and the filtrate was evaporated to dryness under a reduced pressure. The residue was subjected to column chromatography (SiO_2, Hexane/Acetone 1/1) to allow isolation of **20** (20.9 g, 26% yield, 3 steps) as pale yellow oil. ^{1}H NMR (600 MHz, $CDCl_3$): δ 8.09 (d, J = 8.9 Hz, 1H), 7.90 (d, J = 1.4 Hz, 1H), 7.50 (dd, J = 8.9, 2.0 Hz, 1H), 7.45 (d, J = 8.9 Hz, 1H), 7.27 (d, J = 9.6 Hz, 1H), 4.35 (t, J = 4.8 Hz, 2H), 4.29 (t, J = 4.8 Hz, 2H), 3.88 (t, J = 4.8 Hz, 2H), 3.86 (t, J = 4.8 Hz, 2H), 3.74–3.67 (m, 12H), 3.61–3.58 (m, 4H), 2.95 (br, 2H). ^{13}C NMR (150 MHz, $CDCl_3$): δ 147.38, 142.57, 130.86, 129.24, 129.15, 127.88, 123.71, 123.05, 118.25, 118.18, 72.52, 72.49, 72.45, 70.64, 70.47, 70.44, 70.32, 70.28, 69.80, 69.43, 61.54. IR (neat): 3384, 2873, 1587, 1496, 1456, 1348, 1272, 1105, 1070 cm^{-1}. HRMS (EI): m/z calcd for $C_{22}H_{31}{}^{79}BrO_8$ $[M]^+$: 502.1202, found: 502.1206; calcd for $C_{22}H_{31}{}^{81}BrO_8$ $[M]^+$: 504.1185, found: 504.1186.

Compound 21: To a mixture of a CH_2Cl_2 solution (100 ml) of **20** (20.1 g, 40 mmol), triethylamine (56 ml, 400 mmol), and a CH_2Cl_2 solution (100 ml) of *N*,*N*-dimethyl-4-aminopyridine (970 mg, 8.0 mmol) was dropwisely added a CH_2Cl_2 solution (250 ml) of *p*-toluenesulfonyl chloride (33.6 g, 176 mmol) over a period of 2 h at room temperature. The reaction mixture was stirred at room temperature for 26 h, which changed its color to reddish

brown. Then 5 M aqueous HCl solution was carefully added to the reaction mixture, and the mixture was extracted with CH_2Cl_2. The combined organic extract was washed successively with 2 M aqueous HCl solution, water and brine, and an organic phase separated was dried over Na_2SO_4, filtered, and evaporated to dryness under a reduced pressure. The residue was subjected to column chromatography (SiO_2, Hexane/AcOEt 1/1 to 0/1) to allow isolation of **21** (22.0 g, 68% yield) as orange oil. ^1H NMR (600 MHz, $CDCl_3$): δ 8.08 (d, *J* = 8.9 Hz, 1H), 7.89 (d, *J* = 1.4 Hz, 1H), 7.79–7.75 (m, 4H), 7.47–7.44 (m, 2H), 7.32–7.29 (m, 4H), 7.27 (d, *J* = 8.9 Hz, 1H), 4.31 (t, *J* = 4.8 Hz, 2H), 4.27 (t, *J* = 4.8 Hz, 2H), 4.15–4.12 (m, 4H), 3.84 (t, *J* = 4.8 Hz, 2H), 3.77 (t, *J* = 4.8 Hz, 2H), 3.69–3.59 (m, 12H), 2.41 (s, 3H), 2.40 (s, 3H). ^{13}C NMR (150 MHz, $CDCl_3$): δ 147.42, 144.70, 144.67, 142.46, 132.72, 130.76, 129.69, 129.14, 128.99, 127.91, 127.76, 123.80, 122.94, 118.28, 118.07, 72.31, 70.61, 70.50, 70.36, 70.30, 69.84, 69.39, 69.16, 69.13, 68.56, 21.45. IR (neat): 2875, 1589, 1496, 1453, 1354, 1176, 1127, 1098 cm^{-1}. HRMS (FAB): *m*/*z* calcd for $C_{36}H_{43}{}^{79}BrO_{12}S_2$ $[M]^+$: 810.1379, found: 810.1408; calcd for $C_{36}H_{43}{}^{81}BrO_{12}S_2$ $[M]^+$: 812.1365, found: 812.1371.

Compound 16: A MeCN solution (1000 ml) of a mixture of catechol (3.0 g, 27 mmol) and Cs_2CO_3 (44.0 g, 135 mmol) was refluxed for 15 min. To the mixture was dropwisely added a MeCN solution (268 ml) of **21** (21.9 g, 27 mmol) over a period of 1 h at reflux temperature. The reaction mixture was refluxed for 7 days. The mixture was evaporated to dryness under a reduced pressure, and the residue was diluted with $CHCl_3$. The mixture was washed successively with saturated aqueous solution of $NaHCO_3$ and brine, and an organic phase separated was dried over Na_2SO_4, filtered and evaporated to dryness under a reduced pressure. The residue was subjected to column chromatography (SiO_2, MeOH/$CHCl_3$ 1/99), affording oil product. The oil product was subjected to column chromatography (SiO_2, $CHCl_3$/Hexane/AcOEt 1/1/4) to allow isolation of **16** (10.0 g, 64% yield) as pale yellow solid. M.p. 58–61 °C. ^1H NMR (600 MHz, $CDCl_3$): δ 8.02 (d, *J* = 8.9 Hz, 1H), 7.90 (d, *J* = 1.4 Hz, 1H), 7.49 (dd, *J* = 8.9, 2.1 Hz, 1H), 7.45 (d, *J* = 8.9 Hz, 1H), 7.25 (d, *J* = 8.9 Hz, 1H), 6.90–6.87 (m, 4H), 4.34 (t, *J* = 4.8 Hz, 2H), 4.29 (t, *J* = 4.4 Hz, 2H), 4.16–4.14 (m, 4H), 3.95 (t, *J* = 4.8 Hz, 2H), 3.93–3.90 (m, 6H), 3.86–3.83 (m, 4H), 3.82–3.79 (m, 4H). ^{13}C NMR (150 MHz, $CDCl_3$): δ 148.91, 148.84, 147.60, 142.51, 130.70, 129.35, 129.18, 127.77, 123.58, 123.01, 121.47, 121.39, 118.01, 117.46, 114.35, 114.08, 72.39, 71.09, 71.06, 70.62, 70.58, 69.99, 69.89, 69.83, 69.51, 69.32, 69.10. IR (KBr): 3427, 2922, 2886, 1622, 1589, 1501, 1450, 1360, 1329, 1269, 1206, 1135, 1070, 745 cm^{-1}. HRMS (FAB): *m*/*z* calcd for $C_{28}H_{33}{}^{79}BrO_8$ $[M]^+$: 576.1359, found: 576.1351; calcd for $C_{28}H_{33}{}^{81}BrO_8$ $[M]^+$: 578.1343, found: 578.1341.

Compound 22: To a degassed toluene solution (55 ml) of **16** (7.97 g, 13.8 mmol) was added successively $Pd_2(dba)_3$ (885 mg, 0.97 mmol), ^{t}BuXPhos (1.39 g, 2.9 mmol), NaO^{t}Bu (1.86 g, 19.3 mmol) and *tert*-butyl carbamate (1.94 g, 16.6 mmol) at room temperature. After being stirred at room temperature for 8 h, the reaction mixture was filtered through a celite pad, and evaporated to dryness under a reduced pressure. The residue was subjected to column

chromatography (SiO_2, MeOH/$CHCl_3$ 1/99) to allow isolation of **22** (9.5 g, 99% yield) as red oil. ^{1}H NMR (600 MHz, $CDCl_3$): δ 8.05 (d, *J* = 8.9 Hz, 1H), 7.96 (br, 1H), 7.46 (d, *J* = 8.9 Hz, 1H), 7.24 (dd, *J* = 8.9, 2.0 Hz, 1H), 7.20 (d, *J* = 8.9 Hz, 1H), 6.90–6.87 (m, 4H), 6.64 (br, 1H), 4.32 (t, *J* = 4.8 Hz, 2H), 4.27 (t, *J* = 4.1 Hz, 2H), 4.16–4.14 (m, 4H), 3.95 (t, *J* = 4.8 Hz, 2H), 3.93-3.91 (m, 6H), 3.85–3.84 (m, 4H), 3.82–3.79 (m, 4H), 1.54 (s, 9H). ^{13}C NMR (150 MHz, $CDCl_3$): δ 152.84, 148.88, 148.81, 146.26, 142.48, 134.45, 130.33, 125.66, 123.29, 122.45, 121.41, 121.34, 119.44, 117.34, 114.34, 114.10, 80.34, 72.26, 70.98, 70.57, 70.52, 70.03, 69.80, 69.74, 69.63, 69.27, 69.09, 28.25. IR (neat): 3319, 2929, 2873, 1719, 1604, 1543, 1502, 1454, 1369, 1250, 1158, 1128, 1058, 750 cm^{-1}. HRMS (FAB): *m*/*z* calcd for $C_{33}H_{43}NNaO_{10}$ $[M+Na]^+$: 636.2785, found: 636.2781; calcd for $C_{33}H_{43}NO_{10}$ $[M]^+$: 613.2887, found: 613.2893.

Compound 23: 4 M dioxane solution of HCl (80 ml) was added to a reaction vessel containing **22** (9.9 g, 16.1 mmol) at room temperature. After being stirred at room temperature for 7 h, the mixture was evaporated to dryness under a reduced pressure. The residue was directly used for the next step.

Ring component 14: To a MeOH/water (161 ml/161 ml) solution of a mixture of **23** and NaOAc (7.4 g, 90 mmol) was added 2-nitrobenzenesulfonyl chloride (12.5 g, 56 mmol) at room temperature. The reaction mixture was stirred at 50 °C for 12 h. Then $CHCl_3$ was added to the reaction mixture, and the mixture was extracted with $CHCl_3$. The combined organic extract was washed successively with saturated aqueous solution of $NaHCO_3$ and brine, and an organic phase separated was dried over Na_2SO_4, filtered and evaporated to dryness under a reduced pressure. The residue was subjected to column chromatography (SiO_2, MeOH/AcOEt 1/99) to allow isolation of **14** (9.69 g, 86% yield, 2 steps) as pale yellow amorphous. ^{1}H NMR (600 MHz, $CDCl_3$): δ 8.04 (d, *J* = 8.9 Hz, 1H), 7.85 (d, *J* = 7.6 Hz, 1H), 7.78 (dd, *J* = 7.5, 1.3 Hz, 1H), 7.66–7.62 (m, 1H), 7.57 (d, *J* = 2.0 Hz, 1H), 7.51–7.47 (m, 1H), 7.44 (d, *J* = 8.9 Hz, 1H), 7.36 (br, 1H), 7.25–7.22 (m, 2H), 6.90–6.86 (m, 4H), 4.31 (t, *J* = 4.8 Hz, 2H), 4.27 (t, *J* = 4.8 Hz, 2H), 4.15–4.12 (m, 4H), 3.93 (t, *J* = 5.5 Hz, 2H), 3.91–3.89 (m, 6H), 3.84–3.82 (m, 4H), 3.80–3.77 (m, 4H). ^{13}C NMR (150 MHz, $CDCl_3$): δ 148.84, 148.72, 148.02, 147.46, 142.24, 133.85, 132.48, 131.99, 131.66, 131.36, 129.55, 127.55, 125.09, 123.58, 123.30, 122.26, 121.44, 121.31, 120.77, 117.40, 114.34, 113.96, 72.30, 70.94, 70.51, 70.46, 69.87, 69.78, 69.69, 69.44, 69.24, 68.98. IR (neat): 2927, 2874, 1600, 1543, 1503, 1453, 1370, 1255, 1124, 748 cm^{-1}. HRMS (FAB): *m*/*z* calcd for $C_{34}H_{38}N_2O_{12}SNa$ $[M+Na]^+$: 721.2043, found: 721.2037.

Synthesis of endcap component 15

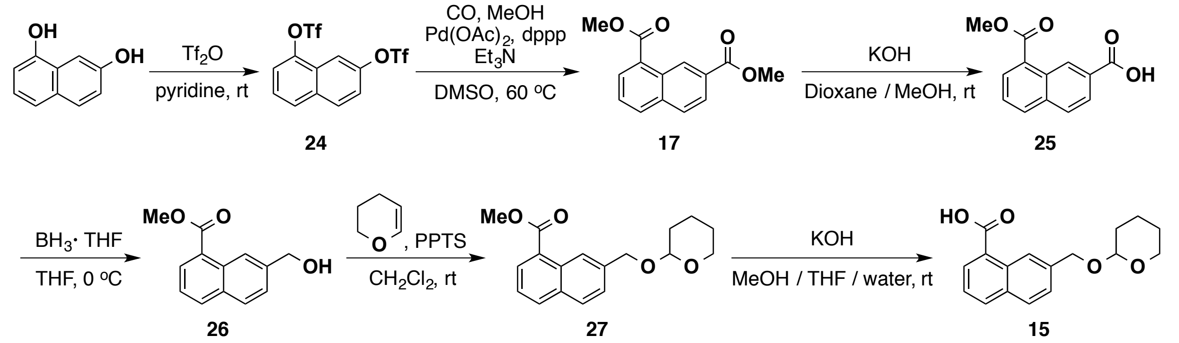

1,7-Naphthylbis(trifluoromethanesulfonate) (24): To a pyridine solution (200 ml) of 1,7-dihydroxynaphthalene (23.4 g, 146 mmol) was dropwisely added trifluoromethanesulfonic anhydride (49 ml, 292 mmol) at 0 °C. The reaction mixture was allowed to warm to room temperature, and stirred for 3 h. Water was added to the reaction mixture, and the mixture was extracted with AcOEt. The combined organic extract was washed successively with 1 M aqueous HCl solution, water and brine, and an organic phase separated was dried over Na_2SO_4, filtered and evaporated to dryness under a reduced pressure. The residue was subjected to column chromatography (SiO_2, Hexane/AcOEt 5/1) to allow isolation of 1,7-naphthylbis(trifluoromethanesulfonate) (**24**) (61.3 g, 99% yield) as colorless oil. ^{1}H NMR (600 MHz, $CDCl_3$): δ 8.04 (d, J = 8.9 Hz, 1H), 7.94–7.97 (m, 2H), 7.63–7.60 (m, 2H), 7.53 (dd, J = 8.9, 2.7 Hz, 1H). ^{13}C NMR (150 MHz, $CDCl_3$): δ 148.43, 145.12, 133.59, 131.07, 128.33, 126.65, 126.42, 121.38, 119.61, 118.85 (q, J = 318.8 Hz), 118.76 (q, J = 318.8 Hz), 112.89. IR (neat): 1606, 1510, 1428, 1215, 1138, 1204 cm^{-1}. HRMS (EI): m/z calcd for $C_{12}H_6F_6O_6S_2$ $[M]^+$: 423.9510, found: 423.9494.

Dimethyl naphthalene-1,7-dicarboxylate (17): To a DMSO/MeOH (153 ml/153 ml) solution of 1,7-naphthylbis(trifluoromethanesulfonate) (**24**) (18.6 g, 43.8 mmol) was added triethylamine (92 ml, 660 mmol) followed by $Pd(OAc)_2$ (1.1 g, 4.38 mmol) and 1,3-Bis(diphenylphosphino)propane (1.8 g, 4.38 mmol) at room temperature. A stream of CO was passed into the solution for 2–3 min, then the reaction vessel and contents were placed in a 55–65 °C oil bath with a CO balloon. After being stirred for 36 h, the reaction mixture was filtered through a celite pad, and a part of solvent was evaporated under a reduced pressure. The mixture was diluted with AcOEt, and extracted with AcOEt. The combined organic extract was washed successively with 1 M aqueous HCl solution, water and brine, and an organic phase separated was dried over Na_2SO_4, filtered and evaporated to dryness under a reduced pressure. The residue was subjected to column chromatography (SiO_2, Hexane/AcOEt 5/1) to allow isolation of dimethyl naphthalene-1,7-dicarboxylate (**17**) (3.47 g, 32% yield) as yellow solid. M.p. 83–85 °C. ^{1}H NMR (600 MHz, $CDCl_3$): δ 9.65 (s, 1H), 8.23 (d, J = 6.2 Hz, 1H), 8.13 (dd, J = 8.9, 1.4 Hz, 1H), 8.04 (d, J = 8.3 Hz, 1H), 7.92 (d, J = 8.3 Hz, 1H), 7.60 (t, J = 8.3 Hz, 1H), 4.03 (s, 3H), 3.99 (s, 3H). ^{13}C NMR (150 MHz, $CDCl_3$): δ 167.27, 167.08, 135.56, 132.87,

130.69, 130.23, 128.84, 128.60, 128.00, 126.58, 125.52, 52.21. IR (KBr): 2949, 1717, 1457, 1435, 1272, 1242, 1196, 1146 cm^{-1}. HRMS (EI): *m*/*z* calcd for $C_{14}H_{12}O_4$ $[M]^+$: 244.0736, found: 244.0740.

8-(Methoxycarbonyl)-2-naphthoic acid (25): To a dioxane solution (360 ml) of dimethyl naphthalene-1,7-dicarboxylate (**17**) (20.0 g, 81.9 mmol) was added a MeOH solution (17 ml) of KOH (4.73 g, 84.3 mmol) at room temperature. The reaction mixture was stirred at room temperature for 2 h. Water was added to the reaction mixture, and the mixture was extracted with AcOEt. The combined organic extract containing dimethyl naphthalene-1,7-dicarboxylate (**17**) was washed with water, and evaporated under a reduced pressure. This recovered dimethyl naphthalene-1,7-dicarboxylate (**17**) was again used for the above reaction. After repeating above reaction three times, the combined aqueous extract was acidified with 1 M aqueous HCl solution to pH 3. The aqueous solution was extracted with AcOEt. The combined organic extract was washed with brine, and an organic phase separated was dried over dried over Na_2SO_4, filtered and evaporated to dryness under a reduced pressure. The residue was subjected to column chromatography (SiO_2, MeOH/$CHCl_3$ 1/99), affording a mixture of 8-(methoxycarbonyl)-2-naphthoic acid (**25**) and 7-(methoxycarbonyl)-1-naphthoic acid. The mixture was recrystallized from $CHCl_3$ to give 8-(methoxycarbonyl)-2-naphthoic acid (**25**) (5.9 g, 31% yield) as white solid. M.p. 229–231 °C. ^{1}H NMR (600 MHz, $CDCl_3$): δ 9.77 (s, 1H), 8.27 (dd, *J* = 6.8, 1.4 Hz, 1H), 8.18 (dd, *J* = 8.9, 1.4 Hz, 1H), 8.09 (d, *J* = 8.3 Hz, 1H), 7.98 (d, *J* = 8.9 Hz, 1H), 7.65 (t, *J* = 6.8 Hz, 1H), 4.06 (s, 3H). ^{13}C NMR (150 MHz, $CDCl_3$): δ 170.15, 167.46, 136.16, 133.03, 130.95, 130.38, 129.99, 128.95, 128.59, 127.97, 127.19, 125.87, 52.47. IR (neat): 3411, 2950, 1713, 1510, 1454, 1436, 1281, 1251, 1226, 1136 cm^{-1}. HRMS (EI): *m*/*z* calcd for $C_{13}H_{10}O_4$ $[M]^+$: 230.0579, found: 230.0579.

Methyl 7-(hydroxymethyl)-1-naphthoate (26): To a THF solution (50 ml) of 8-(methoxycarbonyl)-2-naphthoic acid (**25**) (5.8 g, 25.3 mmol) was added a 1.0 M THF solution of borane tetrahydrofuran complex at 0 °C. The reaction mixture was stirred at 0 °C for 6 h. 1 M aqueous HCl solution was dropwisely added to the reaction mixture, and the mixture was extracted with AcOEt. The combined organic extract was washed successively with water and brine, and an organic phase separated was dried over Na_2SO_4, filtered and evaporated to dryness under a reduced pressure. The residue was subjected to column chromatography (SiO_2, MeOH/$CHCl_3$ 1/50 to 1/20) to allow isolation of methyl 7-(hydroxymethyl)-1-naphthoate (**26**) (4.6 g, 83% yield) as colorless oil. ^{1}H NMR (600 MHz, $CDCl_3$): δ 8.86 (s, 1H), 8.17 (d, *J* = 7.6 Hz, 1H), 8.00 (d, *J* = 8.3 Hz, 1H), 7.87 (d, *J* = 8.2 Hz, 1H), 7.56 (d, *J* = 8.2 Hz, 1H), 7.48 (t, *J* = 8.2 Hz, 1H), 4.88 (s, 2H), 3.99 (s, 3H), 2.12 (br, 1H). ^{13}C NMR (150 MHz, $CDCl_3$): δ 167.98, 140.41, 133.07, 131.10, 130.29, 128.74, 126.66, 125.42, 124.24, 123.05, 65.39, 52.08. IR (KBr): 3411, 3056, 2950, 1711, 1685, 1622, 1461, 1300, 1274, 1249, 1196, 1157, 1111 cm^{-1}. HRMS (EI): *m*/*z* calcd for $C_{13}H_{12}O_3$ $[M]^+$: 216.0786, found: 216.0785.

Compound 27: To a CH_2Cl_2 solution (200 ml) of methyl 7-(hydroxymethyl)-1-naphthoate (**26**) (4.54 g, 21 mmol) and 3,4-dihydro-2*H*-pyran (14.0 ml, 154 mmol) was added pyridinium *p*-toluenesulfonate (503 mg, 2.0 mmol) at room temperature. The reaction mixture was stirred at room temperature for 3 h. Saturated aqueous solution of $NaHCO_3$ was added to the reaction mixture, and the mixture was extracted with CH_2Cl_2. The combined organic extract was washed successively with water and brine, and an organic phase separated was dried over Na_2SO_4, filtered and evaporated to dryness under a reduced pressure. The residue was subjected to column chromatography (SiO_2, Hexane/AcOEt 5/1) to allow isolation of **27** (6.3 g, 99% yield) as colorless oil. ^{1}H NMR (600 MHz, $CDCl_3$): δ 8.88 (s, 1H), 8.18 (dd, *J* = 7.6, 1.4 Hz, 1H), 8.01 (d, *J* = 8.2 Hz, 1H), 7.88 (d, *J* = 8.3 Hz, 1H), 7.59 (dd, *J* = 8.3, 1.4 Hz, 1H), 7.48 (t, *J* = 7.6 Hz, 1H), 4.99 (d, *J* = 12.4 Hz, 1H), 4.78 (t, *J* = 3.4 Hz, 1H), 4.72 (d, *J* = 12.4 Hz, 1H), 4.00 (s, 3H), 4.00–3.95 (m, 1H), 3.61–3.56 (m, 1H), 1.93–1.87 (m, 1H), 1.80–1.75 (m, 1H), 1.73–1.68 (m, 1H), 1.64–1.53 (m, 3H). ^{13}C NMR (150 MHz, $CDCl_3$): δ 167.81, 137.78, 133.18, 132.99, 131.11, 130.25, 128.61, 126.80, 126.09, 124.25, 97.73, 69.05, 62.01, 51.96, 30.46, 25.35, 19.68. IR (neat): 2947, 1717, 1510, 1455, 1437, 1281, 1251, 1135, 1034 cm^{-1}. HRMS (EI): *m*/*z* calcd for $C_{18}H_{20}O_4$ [M]$^+$: 300.1362, found: 300.1366.

Endcap component 15: To a THF/MeOH (210 ml/84 ml) solution of **27** (6.3 g, 20.8 mmol) was added an aqueous solution (42 ml) of KOH (5.3 g, 94 mmol) at room temperature. The reaction mixture was stirred at room temperature for 12 h. A part of solvent was evaporated under a reduced pressure, and the mixture was diluted with $CHCl_3$. The mixture was partitioned between water and $CHCl_3$. The aqueous phase separated was neutralized with an aqueous solution of citric acid, and the mixture was extracted with $CHCl_3$. The combined organic extract was washed successively with water and brine, and an organic phase separated was dried over Na_2SO_4, filtered and evaporated to dryness under a reduced pressure. The residue was subjected to column chromatography (SiO_2, MeOH/AcOEt 1/99) to allow isolation of **15** (5.0 g, 84% yield) as white solid. M.p. 86–89 °C. ^{1}H NMR (600 MHz, $CDCl_3$): δ 9.06 (s, 1H), 8.39 (dd, *J* = 8.2, 1.4 Hz, 1H), 8.07 (d, *J* = 8.4 Hz, 1H), 7.90 (d, *J* = 8.3 Hz, 1H), 7.61 (dd, *J* = 8.9, 1.4 Hz, 1H), 7.53 (t, *J* = 7.6 Hz, 1H), 5.02 (d, *J* = 12.4 Hz, 1H), 4.81 (t, *J* = 3.4 Hz, 1H), 4.76 (d, *J* = 12.4 Hz, 1H), 3.99 (ddd, *J* = 11.0, 8.9, 3.4 Hz, 1H), 3.59 (dt, *J* = 11.0, 4.8 Hz, 1H), 1.95–1.89 (m, 1H), 1.81–1.76 (m, 1H), 1.75–1.70 (m, 1H), 1.65–1.54 (m, 3H). ^{13}C NMR (150 MHz, $CDCl_3$): δ 172.91, 138.27, 134.31, 133.40, 131.95, 131.56, 128.89, 126.26, 125.57, 124.48, 124.44, 97.84, 69.16, 62.17, 30.56, 25.47, 19.28. IR (KBr): 2934, 2870, 2630, 1689, 1593, 1571, 1511, 1458, 1286, 1256, 1124, 1066, 1039 cm^{-1}. HRMS (EI): *m*/*z* calcd for $C_{17}H_{18}O_4$ [M]$^+$: 286.1205, found: 286.1199.

Synthesis of *racemic* rotaxane 1a

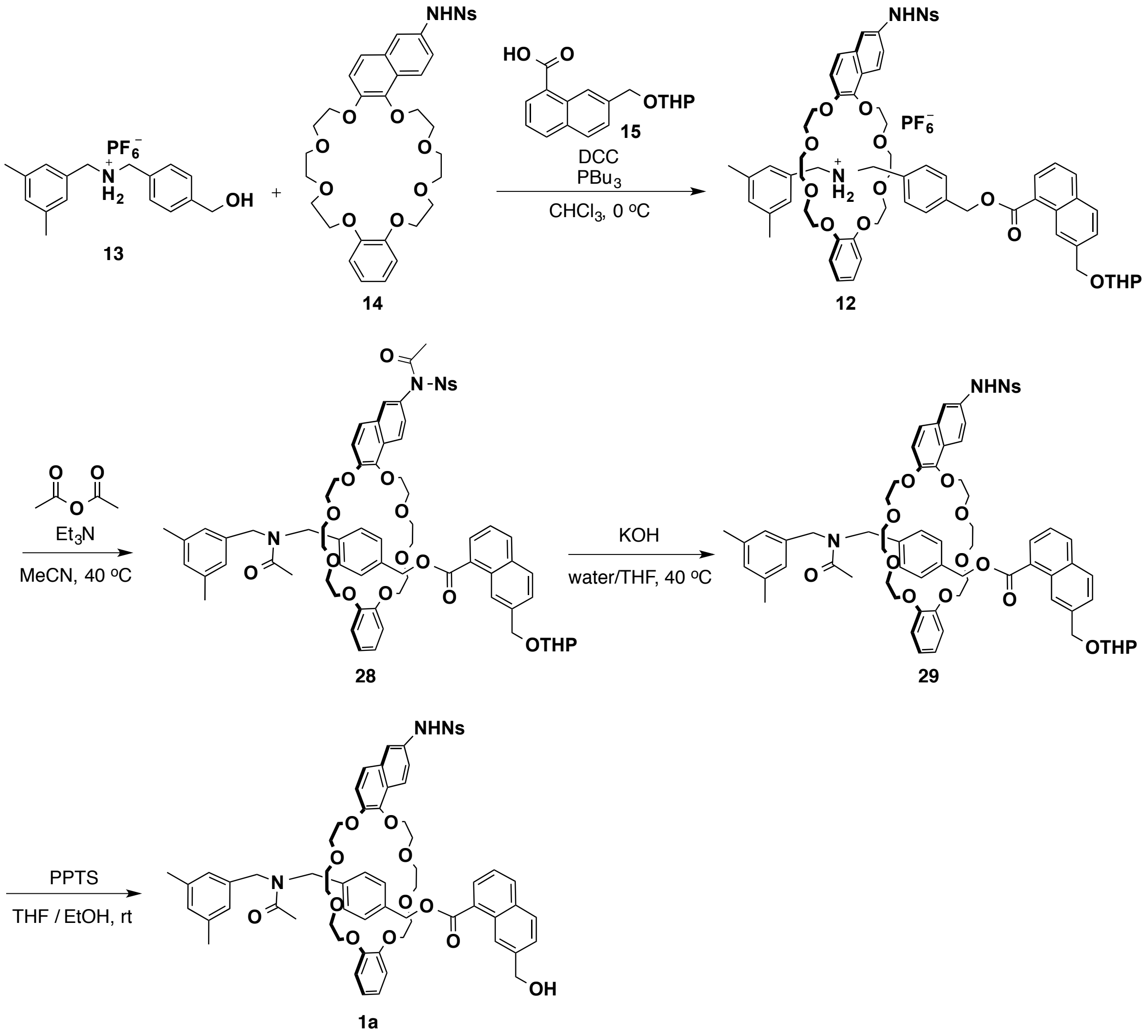

***Racemic* rotaxane 12**: To a $CHCl_3$ solution (20 ml) of ring component **14** (7.2 g, 10.3 mmol) was added axis component **13** (2.8 g, 7.0 mmol). The mixture was stirred at 0 °C for 45 min, which turned to a transparent solution. Endcap component **15** (2.8 g, 9.8 mmol), *N,N'*-dicyclohexylcarbodiimide (5.8 g, 27.9 mmol) and tributylphosphine (345 μl, 1.4 mmol) were successively added to the mixture. The reaction mixture was stirred at 0 °C for 15 h. Then water was added to the reaction mixture, and the mixture was extracted with $CHCl_3$. The combined organic extract was washed successively with water and brine, and an organic phase separated was dried over Na_2SO_4, filtered and evaporated to dryness under a reduced pressure. The residue was subjected to short column chromatography (SiO_2, MeOH/$CHCl_3$ 1/99 to 1/20), affording a mixture of rotaxane **12** and recovered ring component **14**, which was directly used for the next step.

***Racemic* rotaxane 28** (mixture of conformers): To a MeCN solution (70 ml) of the mixture containing rotaxane **12** was added triethylamine (19.4 ml, 139 mmol) and acetic anhydride (6.6

ml, 70 mmol) at room temperature. The reaction mixture was stirred at 40 °C for 3 h. The mixture was evaporated to dryness under a reduced pressure, and the residue was diluted with AcOEt. The mixture washed successively with water and brine, and an organic phase separated was dried over Na_2SO_4, filtered and evaporated to dryness under a reduced pressure. The residue was subjected to column chromatography (SiO_2, hexane/AcOEt 1/2 to MeOH/AcOEt 1/199), affording **28** (4.8 g, 53% yield, 2 steps) as yellow amorphous, which is a mixture of diastereomers derived from a mechanically planar chirality and a chirality of THP group. ^{1}H NMR (600 MHz, $CDCl_3$): δ 9.01 (s, 1H), 8.86–8.83 (m, 1H), 8.43–8.42 (m, 1H), 8.05–8.03 (m, 2H), 7.97–7.95 (m, 2H), 7.78–7.70 (m, 5H), 7.66–7.65 (m, 1H), 7.52–7.50 (m, 1H), 7.46–7.43 (m, 1H), 7.35–7.34 (m, 1H), 7.08–7.02 (m, 2H), 6.99–6.98 (m, 1H), 6.87–6.85 (m, 1H), 6.79–6.77 (m, 3H), 6.74–6.69 (m, 3H), 6.27–6.22 (m, 2H), 4.97–4.93 (m, 1H), 4.78–4.76 (m, 1H), 4.70–4.61 (m, 2H), 4.49 (s, 1H), 4.42–4.37 (m, 2H), 4.28–4.26 (m, 2H), 4.22 (s, 1H), 4.19–4.15 (m, 1H), 4.10–4.02 (m, 4H), 3.99–3.90 (m, 2H), 3.89–3.85 (m, 1H), 3.83–3.79 (m, 1H), 3.71–3.62 (m, 4H), 3.59–3.37 (m, 7H), 3.35–3.31 (m, 1H), 3.28–3.24 (m, 1H), 2.25–2.22 (m, 6H), 2.13–2.12 (m, 3H), 1.99–1.98 (m, 3H), 1.92–1.85 (m, 1H), 1.79–1.66 (m, 2H), 1.63–1.59 (m, 1H), 1.57–1.52 (m, 2H). ^{13}C NMR (mixture of conformers) (150 MHz, $CDCl_3$): δ 170.89, 170.83, 170.81, 167.81, 149.58, 149.55, 148.28, 148.25, 148.22, 148.18, 147.99, 141.70, 141.66, 138.34, 137.88, 137.80, 137.43, 137.20, 136.98, 136.96, 136.89, 136.86, 136.39, 134.86, 134.65, 134.61, 133.92, 132.90, 132.87, 132.54, 131.84, 131.75, 131.60, 131.23, 130.06, 130.02, 129.95, 129.76, 129.61, 129.53, 129.00, 128.97, 128.92, 128.79, 128.40, 128.36, 127.71, 127.16, 126.90, 126.85, 125.89, 125.56, 125.52, 125.33, 124.85, 124.81, 124.15, 123.99, 123.93, 123.48, 122.61, 120.19, 118.00, 115.91, 115.86, 111.30, 111.11, 97.78, 97.72, 71.85, 71.55, 70.98, 70.85, 70.69, 70.25, 70.16, 69.72, 69.63, 69.56, 69.50, 69.35, 69.32, 69.21, 68.15, 68.13, 67.75, 67.66, 62.03, 61.97, 50.45, 49.97, 47.47, 47.34, 30.53, 30.51, 25.39, 24.68, 21.67, 21.15, 21.11, 19.30, 19.26. IR (neat): 3009, 2925, 2874, 1708, 1639, 1597, 1545, 1504, 1452, 1419, 1366, 1252, 1172, 1126, 1061, 1035, 755 cm^{-1}. HRMS (ESI): *m*/*z* calcd for $C_{72}H_{79}N_3O_{18}SNa$ [M+Na]$^+$: 1328.4972, found: 1328.4988.

***Racemic* rotaxane 29**: To a THF solution (85 ml) of rotaxane **28** (4.6 g, 3.5 mmol) was added an aqueous solution (25 ml) of KOH (0.48 g, 8.4 mmol) at room temperature. The reaction mixture was stirred at 40 °C for 4 h. A part of solvent was evaporated under a reduced pressure, and the mixture was extracted with $CHCl_3$. The combined organic extract was washed successively with water and brine, and an organic phase separated was dried over Na_2SO_4, filtered and evaporated to dryness under a reduced pressure. The residue was directly used for the next step.

***Racemic* rotaxane 1a** (mixture of conformers) : To a EtOH/THF (35 ml/35 ml) solution of rotaxane **29** was added pyridinium *p*-toluenesulfonate (17.6 g, 70 mmol) at 30 °C. The reaction mixture was stirred at 30 °C for 19 h. The reaction mixture was evaporated to dryness under a reduced pressure, and the residue was diluted with $CHCl_3$. The mixture was washed

successively with water and brine, and an organic phase separated was dried over Na_2SO_4, filtered and evaporated to dryness under a reduced pressure. The residue was subjected to short column chromatography (SiO_2, hexane/AcOEt 1/5 to 0/1), then subjected to preparative TLC (SiO_2, MeOH/$CHCl_3$ 1/19) to allow isolation of **1a** (1.53 g, 37% yield, 2 steps) as yellow amorphous. HPLC conditions: CHIRALPAK IC column, eluent EtOH/CH_2Cl_2 1/60, flow rate 0.7 ml/min, temperature 20 °C, 18.8 min (minor), 24.5 min (major). $[\alpha]_D^{23}$ –23.4 (major enantiomer, 99% ee, *c* 0.72, $CHCl_3$). ^{1}H NMR (600 MHz, $CDCl_3$): δ 8.83–8.80 (m, 1H), 8.70–8.66 (m, 1H), 8.03 (d, J = 7.9 Hz, 1H), 7.96 (d, J = 7.9 Hz, 1H), 7.83–7,81 (m, 2H), 7.77–7.74 (m, 1H), 7.71–7.64 (m, 2H), 7.62–7.59 (m, 1H), 7.55 (s, 1H), 7.46–7.42 (m, 4H), 7.22–7.18 (m, 2H), 7.10–7.03 (m, 2H), 6.97 (d, J = 8.2 Hz, 1H), 6.88–6.85 (m, 1H), 6.77–6.74 (m, 3H), 6.66–6.63 (m, 3H), 6.25–6.20 (m, 2H), 4.80–4.78 (m, 2H), 4.55–4.44 (m, 2H), 4.37–4.35 (m, 1H), 4.33–4.29 (m, 1H), 4.26–4.24 (m, 1H), 4.22–4.18 (m, 2H), 4.14–4.08 (m, 1H), 4.02–3.96 (m, 4H), 3.90–3.76 (m, 3H), 3.72–3.68 (m, 1H), 3.63–3.59 (m, 3H), 3.54–3.43 (m, 5H), 3.40–3.33 (m, 2H), 3.29–3.26 (m, 1H), 3.21–3.17 (m, 1H), 2.25–2.22 (m, 6H), 2.11–2.07 (m, 3H), 1.74 (br, 1H). ^{13}C NMR (mixture of conformers) (150 MHz, $CDCl_3$): δ 171.06, 170.96, 168.24, 148.30, 148.26, 148.17, 148.15, 148.10, 141.59, 141.58, 139.56, 139.49, 138.47, 137.98, 137.76, 137.37, 137.19, 136.37, 134.91, 133.98, 133.81, 132.79, 132.75, 132.49, 132.19, 131.70, 131.57, 131.42, 131.18, 131.14, 131.11, 131.10, 131.07, 130.06, 129.61, 129.28, 129.25, 129.04, 128.90, 128.50, 128.46, 128.33, 128.24, 127.68, 127.64, 127.12, 125.93, 125.32, 125.12, 124.98, 124.95, 124.26, 123.94, 123.89, 123.77, 123.73, 123.08, 122.47, 122.44, 121.02, 120.22, 115.87, 115.82, 111.30, 111.17, 71.79, 70.87, 70.63, 70.30, 70.21, 69.87, 69.83, 69.79, 69.73, 69.68, 69.63, 69.17, 68.07, 67.89, 67.77, 65.64, 50.52, 50.03, 47.58, 47.46, 21.66, 21.64, 21.22, 21.17. IR (neat): 3011, 2922, 2876, 1705, 1626, 1603, 1543, 1505, 1451, 1369, 1251, 1127, 753 cm^{-1}. HRMS (ESI): *m*/*z* calcd for $C_{65}H_{69}N_3O_{16}SNa$ [M+Na]$^+$: 1202.4291, found: 1202.4322.

Synthesis of ring component 30

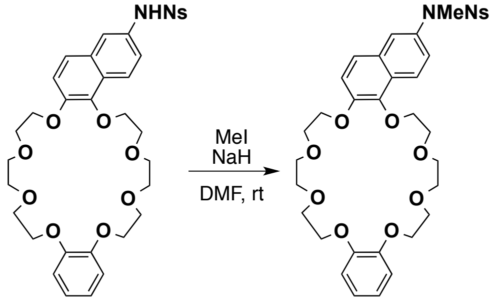

Ring component 30: To a DMF solution (0.66 ml) of ring component **14** (132 mg, 0.189 mmol) was added NaH (9.5 mg, 0.395 mmol), and the mixture was stirred at room temperature for 15 min. To the resulting mixture was added methyl iodide (25 μl, 0.395 mmol), and the reaction mixture was stirred at room temperature for 1 h. Then water was added to the reaction mixture, and the mixture was extracted with AcOEt. The combined organic extract was washed

with water, and an organic phase separated was dried over Na_2SO_4, filtered and evaporated to dryness under a reduced pressure. The residue was subjected to column chromatography (SiO_2, MeOH/AcOEt/Hexane 1/25/25 to MeOH/$CHCl_3$ 1/50) to allow isolation of **30** (130 mg, 96% yield) as yellow oil. ^1H NMR (400 MHz, $CDCl_3$): δ 8.09 (d, J = 9.2 Hz, 1H), 7.66–7.58 (m, 3H), 7.48–7.34 (m, 3H), 7.27–7.24 (m, 2H), 6.89–6.86 (m, 4H), 4.35–4.33 (m, 2H), 4.31–4.29 (m, 2H), 4.16–4.14 (m, 4H), 3.96–3.90 (m, 8H), 3.85–3.79 (m, 8H), 3.45 (s, 3H). ^{13}C NMR (100 MHz, $CDCl_3$): δ 148.83, 148.73, 148.18, 148.04, 142.22, 136.04, 133.60, 131.55, 130.95, 130.91, 129.39, 128.32, 126.08, 124.99, 123.99, 123.59, 123.14, 121.42, 121.31, 117.19, 114.32, 113.98, 72.32, 70.96, 70.54, 70.48, 69.87, 69.80, 69.72, 69.41, 69.21, 68.98, 39.40.IR (neat): 2925, 2874, 1597, 1545, 1502, 1454, 1353, 1256, 1123, 752 cm^{-1}. HRMS (FAB): m/z calcd for $C_{35}H_{40}N_2O_{12}S$ $[M]^+$: 712.2302, found: 712.2302.

Synthesis of *racemic* rotaxane 10

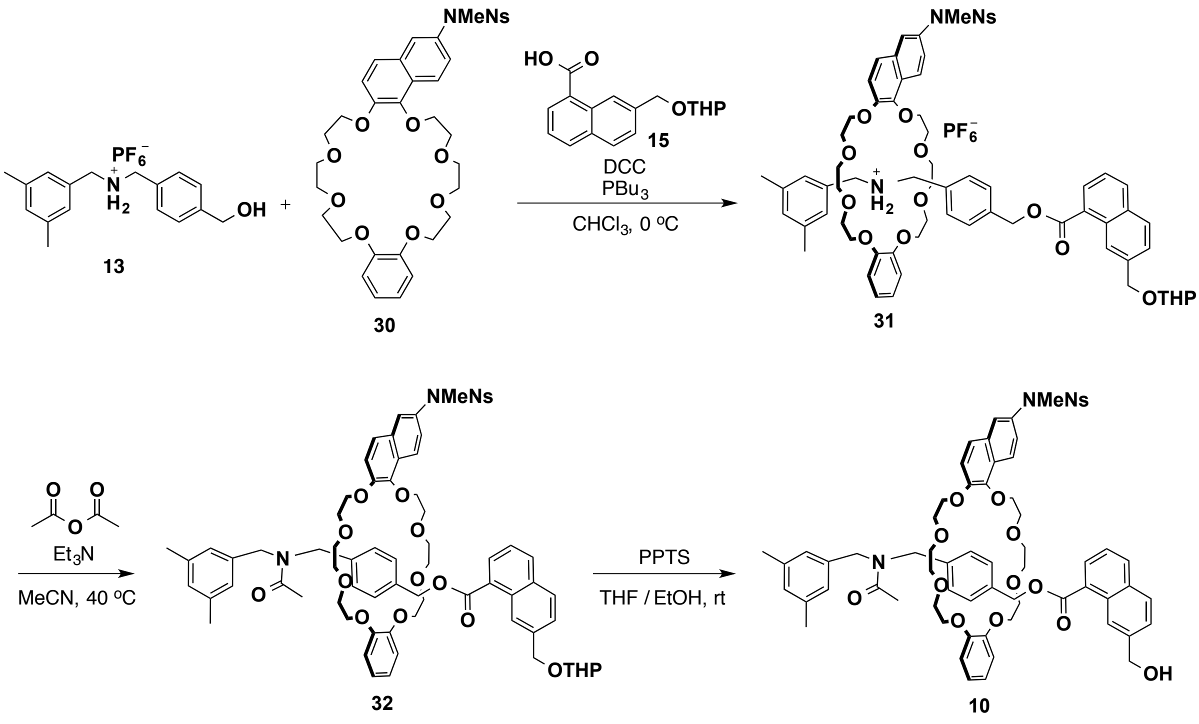

Racemic **rotaxane 31** (mixture of conformers): To a $CHCl_3$ solution (5.2 ml) of ring component **30** (120 mg, 0.168 mmol) was added axis component **13** (94.3 mg, 0.235 mmol). The mixture was stirred at 0 °C for 5 h. To the mixture was successively added endcap component **15** (67.3 mg, 0.235 mmol), tributylphosphine (83 μl, 0.336 mmol) and *N*,*N'*-dicyclohexylcarbodiimide (139 mg, 0.672 mmol). The reaction mixture was stirred at 0 °C for 14 h. The reaction mixture was filtered, and evaporated to dryness under a reduced pressure. The residue was subjected to a preparative TLC (SiO_2, MeOH/$CHCl_3$ 1/19) in order to separate rotaxane **31** from ring component **30**. The resulting product containing **31** was directly used for the next step.

***Racemic* rotaxane 32** (mixture of conformers): To a MeCN solution (940 µl) of **31** was added triethylamine (940 µl, 6.72 mmol) and acetic anhydride (320 µl, 3.36 mmol) at room temperature. The reaction mixture was stirred at 40 °C for 1 h. The mixture was evaporated to dryness under a reduced pressure. The residue was subjected to preparative TLC (SiO_2, MeOH/$CHCl_3$ 1/19), then subjected to preparative TLC (SiO_2, MeOH/AcOEt/Hexane 1/50/50), affording **32** (67.6 mg, 34% yield, 2 steps) as pale yellow amorphous, which is a mixture of diastereomers derived from a mechanically planar chirality and a chirality of THP group. ^{1}H NMR (400 MHz, $CDCl_3$): δ 9.01–8.99 (m, 1H), 8.88–8.85 (m, 1H), 8.02 (d, *J* = 8.3 Hz, 1H), 7.95 (d, *J* = 7.8 Hz, 1H), 7.88–7.85 (m, 1H), 7.76–7.71 (m, 2H), 7.63–7.56 (m, 3H), 7.53–7.49 (m, 2H), 7.44–7.42 (m, 1H), 7.39–7.35 (m, 1H), 7.30–7.27 (m, 1H), 7.19–7.17 (m, 1H), 7.11–7.02 (m, 2H), 6.97–6.95 (m, 1H), 6.88–6.85 (m, 1H), 6.79–6.76 (m, 2H), 6.73–6.68 (m, 3H), 6.66 (s, 1H), 6.23–6.22 (m, 2H), 4.96–4.91 (m, 1H), 4.78–4.75 (m, 1H), 4.69–4.64 (m, 1H), 4.59–4.45 (m, 2H), 4.40–4.33 (m, 2H), 4.25–4.13 (m, 4H), 4.08–3.77 (m, 8H), 3.71–3.22 (m, 17H), 2.25–2.22 (m, 6H), 2.12–2.09 (m, 3H), 1.92–1.83 (m, 1H), 1.78–1.51 (m, 5H). ^{13}C NMR (mixture of conformers) (100 MHz, $CDCl_3$): δ 170.99, 170.89, 167.86, 148.90, 148.27, 141.61, 138.44, 137.96, 137.78, 137.40, 137.21, 137.07, 137.00, 136.41, 135.87, 135.82, 134.90, 133.96, 133.56, 132.99, 132.95, 131.95, 131.83, 131.64, 131.34, 131.08, 130.99, 130.11, 129.66, 129.18, 129.14, 129.02, 128.88, 128.44, 127.67, 127.62, 127,16, 126.31, 125.94, 125.66, 125.60, 125.35, 125.11, 125.08, 124.88, 124.40, 124.34, 124.26, 123.96, 123.63, 122.93, 120.26, 115.73, 115.67, 111.34, 111.19, 97.88, 97.81, 71.79, 70.89, 70.70, 70.33, 70.23, 69.78, 69.72, 69.43, 69.40, 69.26, 68.20, 67.82, 67.76, 62.12, 62.05, 50.47, 50.02, 47.52, 47.36, 39.43, 30.59, 25.45, 21.68, 21.21, 21.17, 19.36, 19.32. IR (neat): 3008, 2924, 2875, 1704, 1641, 1598, 1547, 1504, 1452, 1367, 1252, 1063, 755 cm^{-1}. HRMS (ESI): *m*/*z* calcd for $C_{71}H_{79}N_3O_{17}SNa$ [M+Na]$^+$: 1300.5022, found: 1300.5046.

Rotaxane 10 (mixture of conformers): By a procedure similar to that for *racemic* rotaxane **1a**, preparation of *racemic* rotaxane **10** was performed. Pale yellow amorphous (42 mg, 96% yield). HPLC conditions: After the kinetic resolution of *racemic* **10**, the recovered rotaxane **10** was purified by HPLC and converted to **10-OAc** by *N*,*N*-dimethyl-4-aminopyridine catalyst for ease of chiral HPLC analysis. $[\alpha]_D^{20}$ –20.12 (major enantiomer, 68% ee, *c* 0.43, $CHCl_3$). ^{1}H NMR (400 MHz, $CDCl_3$): δ 8.85–8.82 (m, 1H), 8.72–8.67 (m, 1H), 8.04 (d, *J* = 7.8 Hz, 1H), 7.97 (d, *J* = 8.2 Hz, 1H), 7.89–7.86 (m, 1H), 7.74–7.67 (m, 2H), 7.63–7.57 (m, 3H), 7.52–7.36 (m, 4H), 7.26–7.18 (m, 2H), 7.12–7.04 (m, 2H), 7.00–6.98 (m, 1H), 6.88–6.85 (m, 1H), 6.77–6.74 (m, 3H), 6.67–6.63 (m, 3H), 6.27–6.24 (m, 2H), 4.81–4.79 (m, 2H), 4.61–4.53 (m, 1H), 4.50–4.30 (m, 3H), 4.27–4.18 (m, 3H), 4.15–4.07 (m, 1H), 4.04–3.94 (m, 4H), 3.91–3.77 (m, 3H), 3.73–3.60 (m, 4H), 3.57–3.33 (m, 10H), 3.30–3.26 (m, 1H), 3.22–3.17 (m, 1H), 2.25–2.21 (m, 6H), 2.12–2.10 (m, 3H). ^{13}C NMR (mixture of conformers) (100 MHz, $CDCl_3$): δ 171.07, 170.93, 168.37, 168.34, 148.87, 148.30, 148.19, 141.62, 139.61, 139.53, 138.49, 138.01, 137.78, 137.37, 137.19, 136.39, 135.92, 135.86, 134.98, 134.05, 133.58, 132.84,

132.79, 131.68, 131.50, 131.16, 131.01, 130.07, 129.63, 129.21, 129.18, 129.07, 128.93, 128.57, 128.53, 128.47, 128.44, 128.39, 128.29, 127.17, 126.34, 125.96, 125.40, 125.18, 125.12, 125.01, 124.97, 124.43, 124.37, 124.31, 123.98, 123.76, 123.72, 123.68, 122.95, 120.26, 115.76, 115.70, 111.33, 111.19, 71.85, 70.91, 70.66, 70.33, 70.24, 69.90, 69.86, 69.70, 69.22, 68.09, 67.96, 67.81, 65.71, 50.52, 50.09, 47.59, 47.43, 39.48, 21.70, 21.24, 21.18. IR (neat): 3418, 3012, 2925, 2877, 1704, 1631, 1600, 1547, 1504, 1453, 1367, 1252, 1128, 1061, 755 cm^{-1}. HRMS (ESI): *m*/*z* calcd for $C_{66}H_{71}N_3O_{16}SNa$ $[M+Na]^+$: 1216.4447, found: 1216.4479.

Synthesis of ring component 35

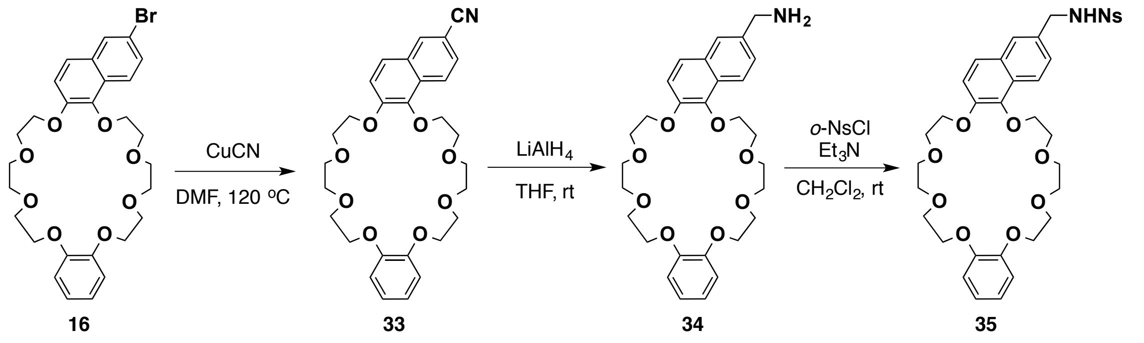

Compound 33: To a DMF solution (30 ml) of **16** (4.1 g, 6.9 mmol) was added CuCN (12.4 g, 138 mmol) at room temperature. The reaction mixture was stirred at 120 °C for 70 h. Water was added to the reaction mixture, and the mixture was extracted with AcOEt. The combined organic extract was washed successively with saturated aqueous solution of $NaHCO_3$, and an organic phase separated was dried over Na_2SO_4, filtered and evaporated to dryness under a reduced pressure. The residue was subjected to short column chromatography (SiO_2, Hexane/AcOEt 1/1 to MeOH/$CHCl_3$ 1/9), affording crude product containing **33** as brown oil, which was directly used for the next step.

Compound 34: To a THF solution (12.5 ml) of $LiAlH_4$ (410 mg, 10.8 mmol) was added a THF solution (12.5 ml) of **33** (1.88 g) at 0 °C. The reaction mixture was stirred at room temperature for 10 h. To the reaction mixture was added water, followed by 15% aqueous solution of NaOH and water. The resulting precipitate was filtered through celite, and a part of solvent was evaporated under a reduced pressure. The mixture was extracted with $CHCl_3$. The combined organic extract was washed with brine, and an organic phase separated was dried over Na_2SO_4, filtered and evaporated to dryness under a reduced pressure. The residue was directly used for the next step.

Ring component 35: To a CH_2Cl_2 solution (10 ml) of a mixture of **34** and triethylamine (550 μl, 3.95 mmol) was added 2-nitrobenzenesulfonyl chloride (875 mg, 3.95 mmol) at room temperature. The reaction mixture was stirred at room temperature for 1 h. The reaction mixture

was diluted with $CHCl_3$. The mixture was washed successively with 1 M aqueous HCl solution and brine, and an organic phase separated was dried over Na_2SO_4, filtered and evaporated to dryness under a reduced pressure. The residue was subjected to column chromatography (SiO_2, MeOH/$CHCl_3$ 1/99) to allow isolation of **35** (2.05 g, 42% yield, 3 steps) as reddish brown amorphous. ^{1}H NMR (400 MHz, $CDCl_3$): δ 7.95 (d, J = 8.7 Hz, 1H), 7.89 (dd, J = 7.3, 0.9 Hz, 1H), 7.74 (d, J = 7.8 Hz, 1H), 7.56–7.51 (m, 2H), 7.44–7.39 (m, 2H), 7.23–7.21 (m, 2H), 6.89–6.86 (m, 4H), 5.84 (brt, J = 6.0 Hz, 1H), 4.44 (d, J = 6.0 Hz, 2H), 4.30–4.27 (m, 4H), 4.17–4.13 (m, 4H), 3.95–3.89 (m, 8H), 3.86–3.78 (m, 8H). ^{13}C NMR (100 MHz, $CDCl_3$): δ 148.82, 148.72, 147.64, 147.44, 142.09, 133.75, 133.13, 132.34, 131.16, 130.76, 129.08, 128.53, 126.65, 125.47, 124.92, 123.77, 122.23, 121.43, 121.32, 116.65, 114.29, 113.98, 72.25, 70.96, 70.52, 70.44, 69.92, 69.77, 69.71, 69.32, 69.21, 68.97, 47.85. IR (neat): 3012, 2927, 2875, 1600, 1541, 1502, 1345, 1255, 1125, 751 cm^{-1}. HRMS (FAB): m/z calcd for $C_{35}H_{40}N_2O_{12}S$ $[M]^+$: 712.2302, found: 712.2304.

Synthesis of *racemic* rotaxane 11

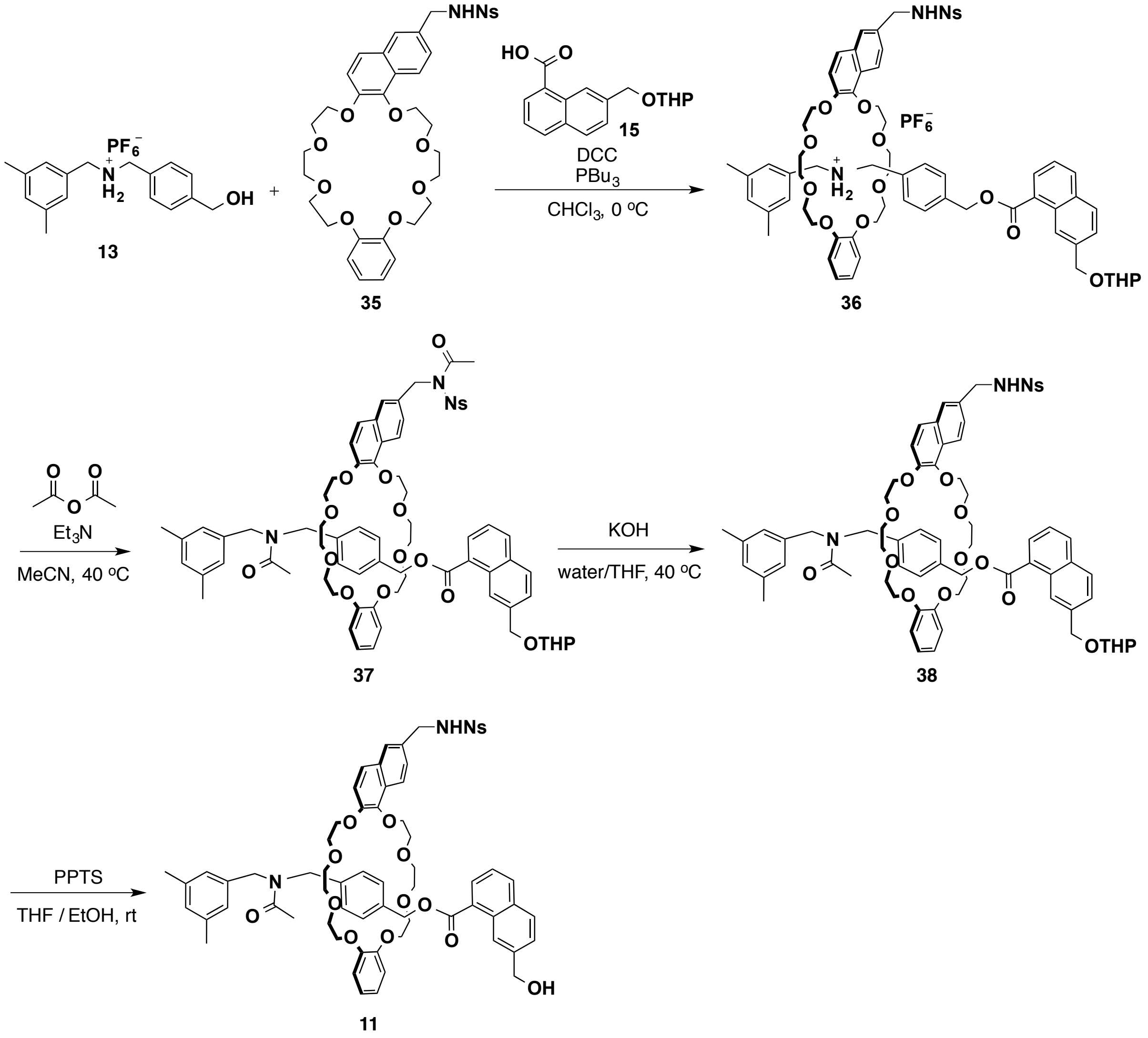

***Racemic* rotaxane 36**: By a procedure similar to that for *racemic* rotaxane **12**, preparation of *racemic* rotaxane **36** was performed.

***Racemic* rotaxane 37** (mixture of conformers): By a procedure similar to that for *racemic* rotaxane **28**, acylative neutralization of **36** was performed. Pale yellow oil (26 mg, 25% yield, 2 steps). ^{1}H NMR (400 MHz, $CDCl_3$): δ 9.01 (s, 1H), 8.89–8.87 (m, 1H), 8.44–8.40 (m, 1H), 8.03 (d, *J* = 7.8 Hz, 1H), 7.97–7.93 (m, 2H), 7.79–7.70 (m, 6H), 7.60–7.58 (m, 1H), 7.52–7.49 (m, 1H), 7.39–7.36 (m, 1H), 7.30–7.27 (m, 1H), 7.11–7.04 (m, 2H), 6.98–6.96 (m, 1H), 6.86–6.84 (m, 1H), 6.80–6.72 (m, 5H), 6.65 (s, 1H), 6.25–6.23 (m, 2H), 5.16 (s, 2H), 4.95–4.92 (m, 1H), 4.77–4.75 (m, 1H), 4.69–4.65 (m, 1H), 4.58–4.47 (m, 2H), 4.41–4.36 (m, 2H), 4.25–4.16 (m, 4H), 4.10–4.03 (m, 4H), 3.99–3.78 (m, 4H), 3.72–3.23 (m, 14H), 2.23–2.22 (m, 6H), 2.15–2.10 (m, 6H), 1.94–1.83 (m, 1H), 1.79–1.51 (m, 4H). ^{13}C NMR (mixture of conformers) (100 MHz, $CDCl_3$): δ 171.05, 170.99, 170.96, 167.88, 148.57, 148.50, 148.37, 148.35, 148.31, 148.28, 147.86, 141.69, 138.46, 137.99, 137.84, 137.46, 137.27, 137.08, 137.01, 136.46, 134.92, 134.60, 134.57, 133.99, 132.97, 132.02, 131.93, 131.50, 131.46, 131.39, 130.07, 129.65, 129.30, 129.27, 129.02, 128.90, 128.79, 128.49, 127.62, 127.25, 126.00, 125.66, 125.63, 125.43, 125.32, 124.93, 124.63, 124.56, 124.33, 123.99, 122.58, 120.30, 115.47, 115.41, 111.39, 111.24, 97.90, 97.82, 71.84, 70.90, 70.79, 70.29, 70.20, 69.82, 69.77, 69.48, 69.46, 69.24, 68.27, 67.86, 67.78, 62.15, 62.08, 51.25, 50.52, 50.00, 47.55, 47.41, 30.62, 30.61, 29.68, 25.49, 24.30, 21.73, 21.23, 21.20, 19.39, 19.34. IR (neat): 3009, 2925, 2875, 1707, 1642, 1543, 1504, 1452, 1366, 1251, 1172, 1126, 1061, 1034, 753 cm^{-1}. HRMS (ESI): *m*/*z* calcd for $C_{73}H_{81}N_3O_{18}SNa$ $[M+Na]^+$: 1342.5128, found: 1342.5146.

***Racemic* rotaxane 38**: By a procedure similar to that for *racemic* rotaxane **29**, hydrolysis of **37** was performed.

Rotaxane 11 (mixture of conformers): By a procedure similar to that for *racemic* rotaxane **1a**, preparation of *racemic* rotaxane **11** was performed. Pale yellow amorphous (8.4 mg, 36% yield, 2 steps). HPLC conditions: CHIRALPAK ID column, eluent EtOH/CH_2Cl_2 1/40, flow rate 0.5 ml/min, temperature 20 °C, 21.9 min (major), 24.1 min (minor). $[\alpha]_D^{20}$ –27.37 (major enantiomer, 63% ee, *c* 1.21, $CHCl_3$). ^{1}H NMR (400 MHz, $CDCl_3$): δ 8.85–8.84 (m, 1H), 8.77–8.73 (m, 1H), 8.02 (d, *J* = 8.3 Hz, 1H), 7.94 (d, *J* = 8.3 Hz, 1H), 7.89–7.85 (m, 1H), 7.74–7.66 (m, 4H), 7.57–7.54 (m, 1H), 7.45–7.30 (m, 4H), 7.22–7.09 (m, 3H), 7.03–6.95 (m, 2H), 6.88–6.85 (m, 1H), 6.79–6.72 (m, 3H), 6.69–6.66 (m, 3H), 6.25–6.22 (m, 2H), 6.03–5.92 (m, 1H), 4.79–4.77 (m, 2H), 4.52–4.33 (m, 6H), 4.26–4.11 (m, 4H), 4.06–3.99 (m, 4H), 3.90–3.80 (m, 3H), 3.73–3.68 (m, 1H), 3.65–3.45 (m, 8H), 3.40–3.21 (m, 4H), 2.25–2.22 (m, 6H), 2.12–2.09 (m, 3H). ^{13}C NMR (mixture of conformers) (100 MHz, $CDCl_3$): δ 171.20, 170.96, 168.17, 148.41, 148.24, 148.20, 148.18, 148.16, 147.58, 141.53, 141.44, 139.65, 139.59, 138.48, 138.00, 137.78, 137.36, 137.17, 136.34, 134.92, 134.00, 133.91, 133.09, 133.06, 132.84,

132.82, 132.34, 132.30, 131.73, 131.61, 131.36, 131.19, 130.96, 130.84, 129.99, 129.59, 129.05, 128.92, 128.88, 128.82, 128.67, 128.64, 128.56, 128.54, 128.20, 128.10, 127.13, 126.95, 126.89, 125.95, 125.62, 125.39, 124.97, 124.93, 124.29, 124.16, 124.07, 123.96, 123.71, 123.67, 122.03, 120.27, 115.14, 111.34, 111.22, 71.75, 70.86, 70.67, 70.26, 70.21, 69.91, 69.77, 69.70, 69.12, 69.10, 68.13, 67.91, 67.78, 65.65, 50.49, 50.04, 47.93, 47.89, 47.53, 47.41, 21.67, 21.23, 21.18. IR (neat): 3441, 3011, 2925, 2877, 1702, 1630, 1541, 1504, 1452, 1344, 1251, 1128, 1061, 753 cm^{-1}. HRMS (ESI): *m*/*z* calcd for $C_{66}H_{71}N_3O_{16}SNa$ $[M+Na]^+$: 1216.4447, found: 1216.4475.

Characterization data for rotaxane 1a-OAc, 10-OAc, 11-OAc

Rotaxane 1a-OAc (mixture of conformers): Yellow amorphous. HPLC conditions: CHIRALPAK IC column, eluent EtOH/CH_2Cl_2 1/60, flow rate 0.7 ml/min, temperature 20 °C, 9.2 min (major), 12.6 min (minor). $[\alpha]_D^{23}$ +9.89 (major enantiomer, 40% ee, *c* 1.16, $CHCl_3$). ^1H NMR (600 MHz, $CDCl_3$): δ 9.02–9.00 (m, 1H), 8.88–8.86 (m, 1H), 8.02 (d, *J* = 8.2 Hz, 1H), 7.94 (d, *J* = 7.9 Hz, 1H), 7.83–7.81 (m, 2H), 7.75–7.69 (m, 3H), 7.63–7.59 (m, 1H), 7.56–7.55 (m, 1H), 7.48–7.42 (m, 3H), 7.35 (br, 1H), 7.25–7.24 (m, 1H), 7.20–7.18 (m, 1H), 7.12–7.07 (m, 1H), 7.03–7.01 (m, 1H), 6.97–6.95 (m, 1H), 6.89–6.86 (m, 1H), 6.76–6.73 (m, 3H), 6.68–6.66 (m, 3H), 6.24–6.20 (m, 2H), 5.26–5.24 (m, 2H), 4.55–4.44 (m, 2H), 4.38–4.33 (m, 2H), 4.25–4.12 (m, 4H), 4.07–4.00 (m, 4H), 3.92–3.77 (m, 3H), 3.71–3.68 (m, 1H), 3.63–3.60 (m, 3H), 3.54–3.36 (m, 6H), 3.33–3.29 (m, 1H), 3.25–3.22 (m, 1H), 2.25–2.22 (m, 6H), 2.13–2.07 (m, 6H). ^{13}C NMR (mixture of conformers) (150 MHz, $CDCl_3$): δ 171.01, 170.89, 167.77, 148.35, 148.27, 148.21, 148.16, 141.68, 138.49, 138.01, 137.78, 137.40, 137.38, 137.26, 136.46, 134.99, 134.45, 134.36, 134.05, 133.83, 133.14, 132.54, 132.23, 132.07, 131.88, 131.79, 131.24, 131.18, 131.14, 130.21, 129.77, 129.33, 129.05, 128.91, 128.67, 128.64, 127.82, 127.78, 127.17, 125.96, 125.85, 125.82, 125.54, 125.51, 125.32, 125.19, 124.75, 123.95, 123.16, 122.49, 121.13, 120.25, 115.93, 115.86, 111.30, 111.15, 71.82, 70.95, 70.69, 70.41, 70.32, 69.83, 69.77, 69.28, 68.20, 67.98, 67.85, 66.94, 50.52, 50.04, 47.58, 47.45, 21.70, 21.25, 21.20, 21.05. IR (neat): 2958, 2926, 2874, 1731, 1631, 1602, 1543, 1506, 1453, 1370, 1252, 1170, 1125, 1065, 753 cm^{-1}. HRMS (ESI): *m*/*z* calcd for $C_{67}H_{71}N_3O_{17}SNa$ $[M+Na]^+$: 1244.4396, found: 1244.4416.

Rotaxane 10-OAc (mixture of conformers): Pale yellow amorphous. HPLC conditions: CHIRALPAK ID column, eluent EtOH/$CHCl_3$ 1/540, flow rate 0.7 ml/min, temperature 20 °C, 6.6 min (minor), 7.1 min (major). $[\alpha]_D^{20}$ +19.07 (major enantiomer, 62% ee, *c* 1.17, $CHCl_3$). ^1H NMR (400 MHz, $CDCl_3$): δ 9.02–9.00 (m, 1H), 8.90–8.86 (m, 1H), 8.03 (d, *J* = 8.2 Hz, 1H), 7.96 (d, *J* = 8.2 Hz, 1H), 7.88–7.86 (m, 1H), 7.77–7.71 (m, 2H), 7.62–7.56 (m, 3H), 7.51–7.49 (m, 1H), 7.45–7.42 (m, 2H), 7.40–7.35 (m, 1H), 7.29–7.26 (m, 1H), 7.20–7.17 (m, 1H), 7.14–7.07 (m, 1H), 7.04 (d, *J* = 7.8 Hz, 1H), 6.98 (d, *J* = 7.8 Hz, 1H), 6.88–6.85 (m, 1H), 6.76–6.73

(m, 3H), 6.70–6.67 (m, 3H), 6.25–6.22 (m, 2H), 5.26–5.24 (m, 2H), 4.60–4.52 (m, 1H), 4.49–4.46 (m, 1H), 4.40–4.34 (m, 2H), 4.27–4.12 (m, 4H), 4.07–4.00 (m, 4H), 3.94–3.78 (m, 3H), 3.72–3.61 (m, 4H), 3.53–3.36 (m, 10H), 3.33–3.29 (m, 1H), 3.26–3.22 (m, 1H), 2.25–2.22 (m, 6H), 2.12–2.09 (m, 6H). ^{13}C NMR (mixture of conformers) (100 MHz, $CDCl_3$): δ 171.06, 170.92, 167.84, 148.93, 148.90, 148.31, 148.26, 148.19, 141.65, 141.63, 138.50, 138.01, 137.79, 137.40, 137.22, 136.42, 135.92, 135.88, 135.00, 134.46, 134.38, 134.06, 133.57, 133.14, 133.12, 132.11, 131.95, 131.83, 131.69, 131.26, 131.16, 131.00, 130.20, 129.76, 129.23, 129.19, 129.07, 128.93, 128.70, 128.67, 128.50, 128.47, 127.81, 127.76, 127.19, 126.35, 125.97, 125.84, 125.81, 125.58, 125.54, 125.39, 125.17, 125.11, 124.77, 124.45, 124.39, 123.99, 123.67, 122.96, 120.27, 115.74, 115.68, 111.31, 111.16, 71.85, 70.96, 70.70, 70.41, 70.31, 69.84, 69.75, 69.29, 68.21, 68.00, 67.85, 66.95, 50.51, 50.08, 47.59, 47.42, 39.48, 21.71, 21.24, 21.19, 21.04. IR (neat): 2925, 2855, 1738, 1706, 1641, 1599, 1547, 1504, 1454, 1369, 1254, 1124, 757 cm^{-1}. HRMS (ESI): *m*/*z* calcd for $C_{68}H_{73}N_3O_{17}SNa$ [M+Na]$^+$: 1258.4553, found: 1258.4569.

Rotaxane 11-OAc (mixture of conformers): Pale yellow amorphous. HPLC conditions: CHIRALPAK ID column, eluent EtOH/CH_2Cl_2 1/80, flow rate 1.0 ml/min, temperature 20 °C, 9.2 min (minor), 9.8 min (major). $[\alpha]_D^{20}$ +19.13 (major enantiomer, 64% ee, *c* 0.79, $CHCl_3$). ^{1}H NMR (600 MHz, $CDCl_3$): δ 9.02 (s, 1H), 8.93–8.90 (m, 1H), 8.01 (d, *J* = 8.3 Hz, 1H), 7.92 (d, *J* = 8.3 Hz, 1H), 7.87–7.82 (m, 1H), 7.78–7.65 (m, 4H), 7.57–7.55 (m, 1H), 7.47–7.21 (m, 5H), 7.17–7.13 (m, 2H), 7.01–6.95 (m, 2H), 6.88–6.85 (m, 1H), 6.77–6.66 (m, 6H), 6.27–6.18, (m, 2H), 6.01–5.88 (m, 1H), 5.26–5.20 (m, 2H), 4.50–4.32 (m, 6H), 4.28–4.00 (m, 8H), 3.92–3.80 (m, 3H), 3.73–3.68 (m, 1H), 3.64–3.26 (m, 12H), 2.25–2.22 (m, 6H), 2.12–2.08 (m, 6H). ^{13}C NMR (mixture of conformers) (150 MHz, $CDCl_3$): δ 171.11, 170.93, 167.74, 167.73, 148.49, 148.37, 148.32, 148.29, 148.26, 147.66, 141.54, 141.51, 138.50, 138.02, 137.82, 137.40, 137.25, 136.44, 135.00, 134.55, 134.48, 134.08, 134.02, 133.18, 133.09, 133.05, 132.33, 132.28, 132.18, 131.96, 131.87, 131.32, 131.00, 130.96, 130.89, 130.14, 129.75, 129.07, 128.93, 128.77, 128.71, 128.69, 128.58, 127.85, 127.79, 127.18, 127.01, 126.94, 126.00, 125.86, 125.80, 125.64, 125.59, 125.41, 124.99, 124.94, 124.80, 124.17, 124.08, 124.00, 121.11, 120.33, 120.30, 120.28, 115.21, 111.37, 111.24, 71.79, 70.95, 70.75, 70.38, 70.34, 69.97, 69.83, 69.79, 69.74, 69.68, 69.24, 69.20, 68.26, 68.01, 67.88, 66.94, 50.54, 50.09, 48.02, 47.97, 47.56, 47.46, 21.69, 21.25, 21.21, 21.05. IR (neat): 3012, 2924, 2878, 1738, 1707, 1633, 1603, 1541, 1452, 1346, 1251, 1128, 1062, 754 cm^{-1}. HRMS (ESI): *m*/*z* calcd for $C_{68}H_{73}N_3O_{17}SNa$ [M+Na]$^+$: 1258.4553, found: 1258.4613.

5. ^{1}H and ^{13}C NMR Spectroscopy

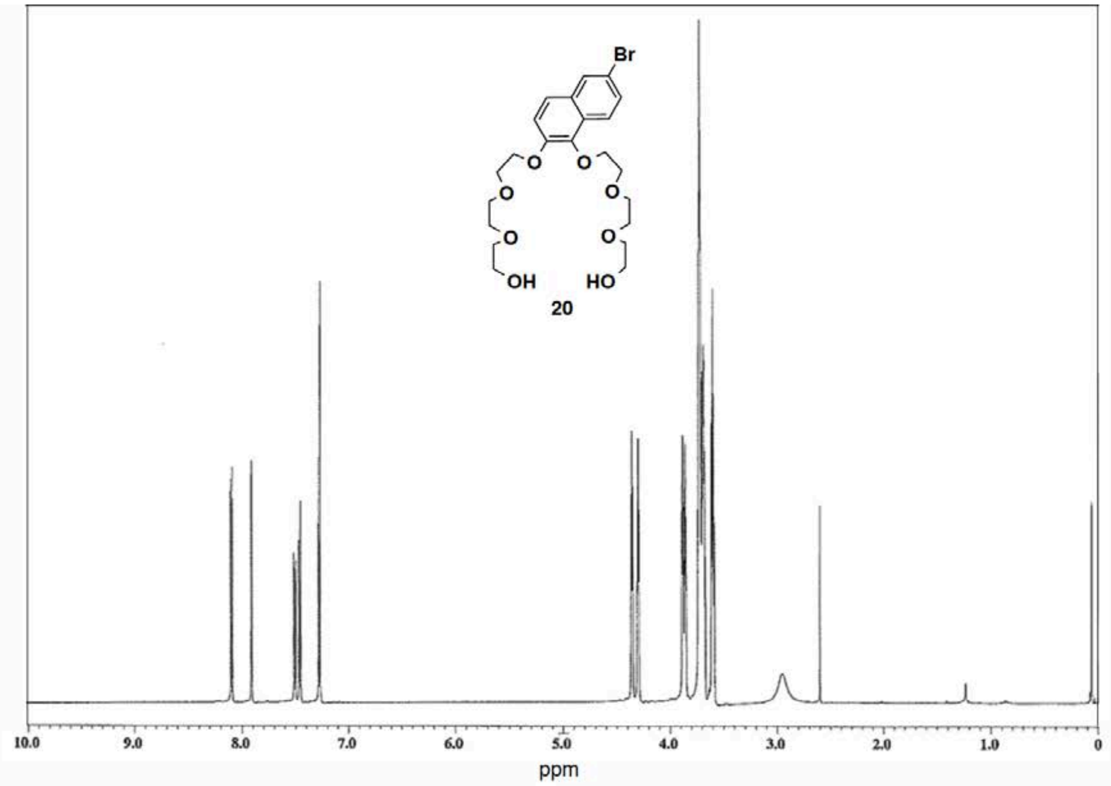

Supplementary Figure 8. ^{1}H NMR spectrum (600 MHz) of **20** in $CDCl_3$.

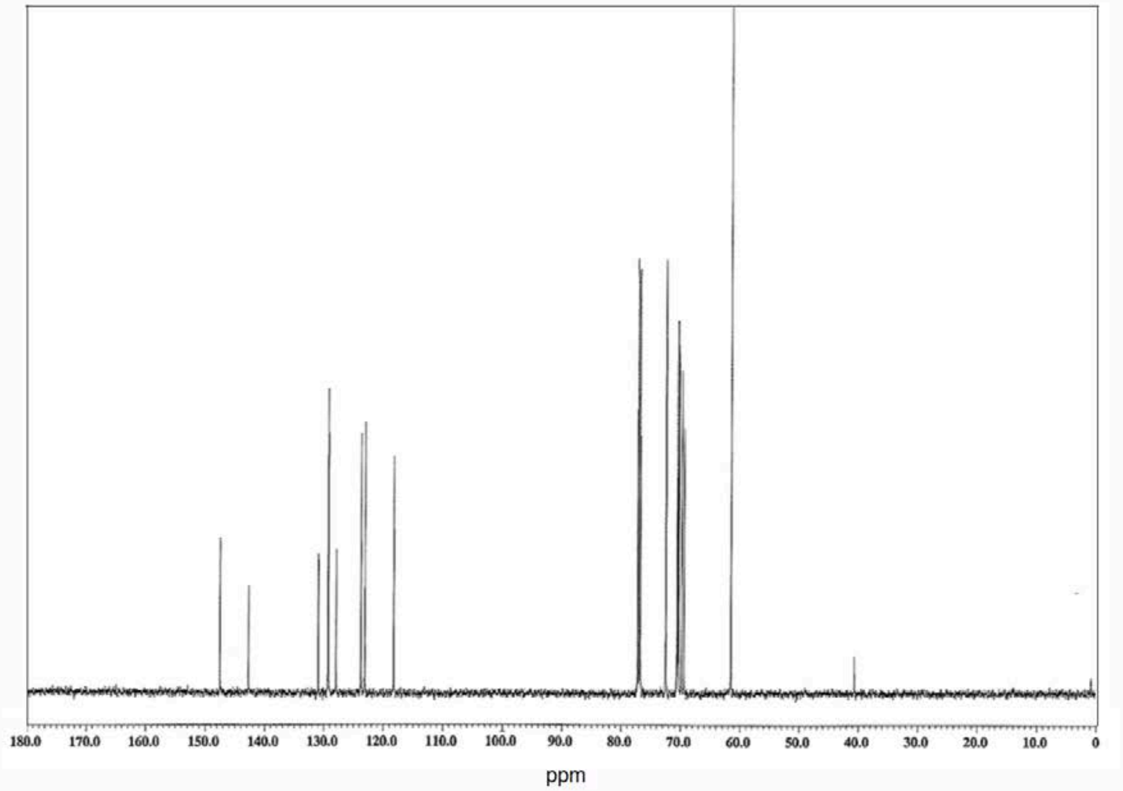

Supplementary Figure 9. ^{13}C NMR spectrum (150 MHz) of **20** in $CDCl_3$.

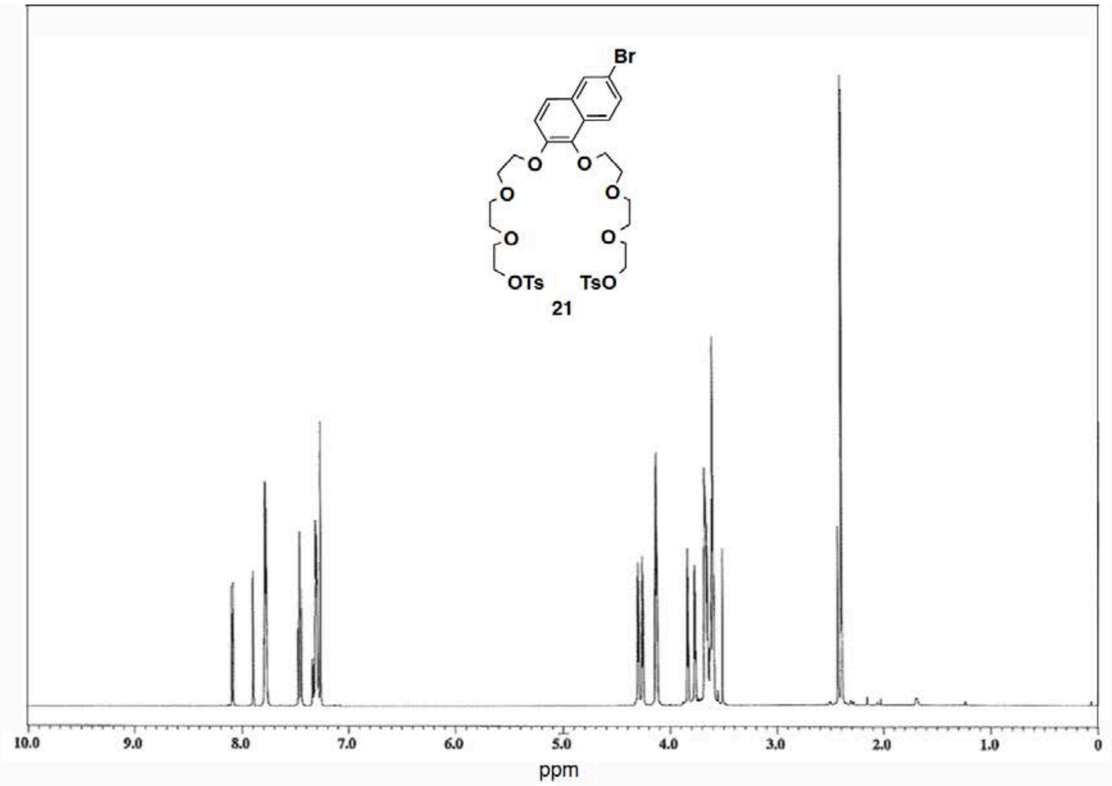

Supplementary Figure 10. ^{1}H NMR spectrum (600 MHz) of **21** in $CDCl_3$.

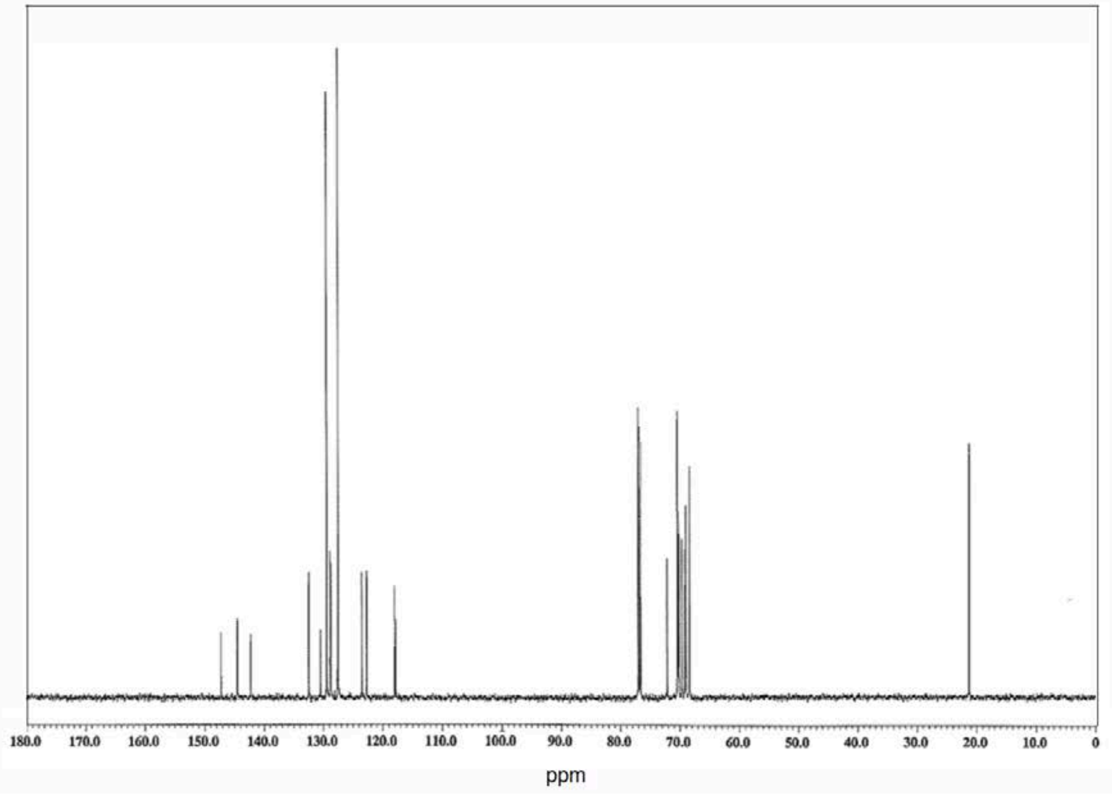

Supplementary Figure 11. ^{13}C NMR spectrum (150 MHz) of **21** in $CDCl_3$.

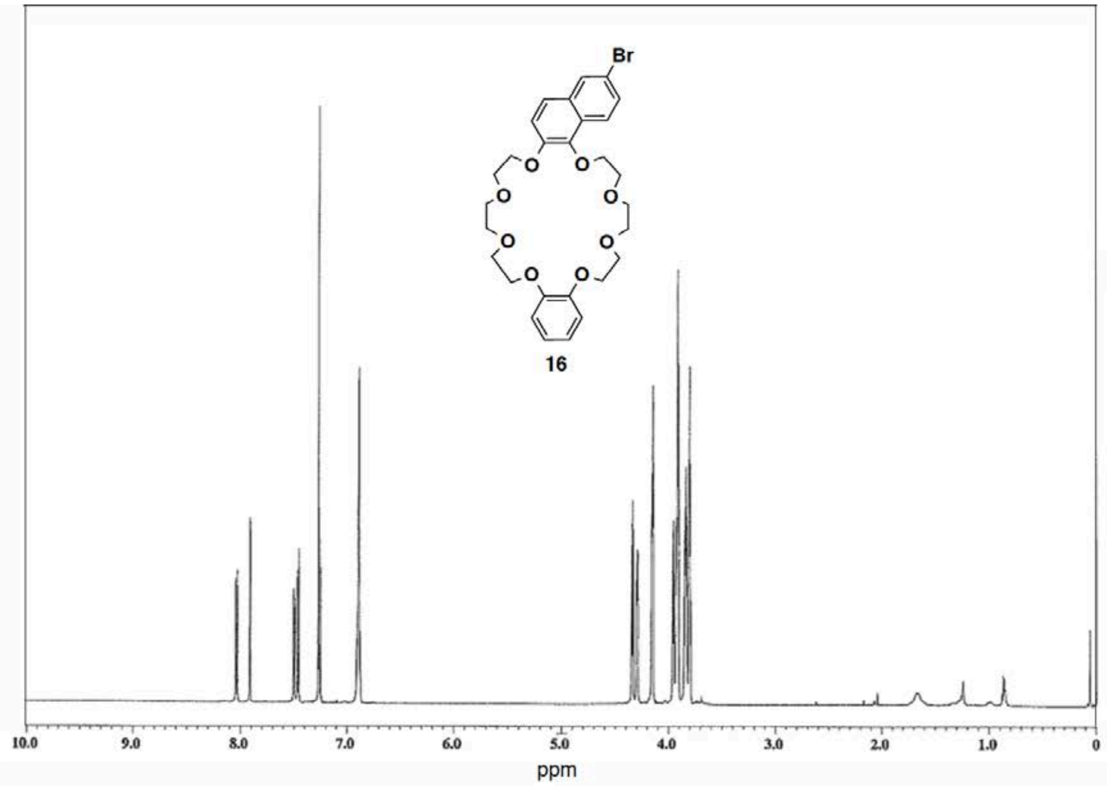

Supplementary Figure 12. ^{1}H NMR spectrum (600 MHz) of **16** in $CDCl_3$.

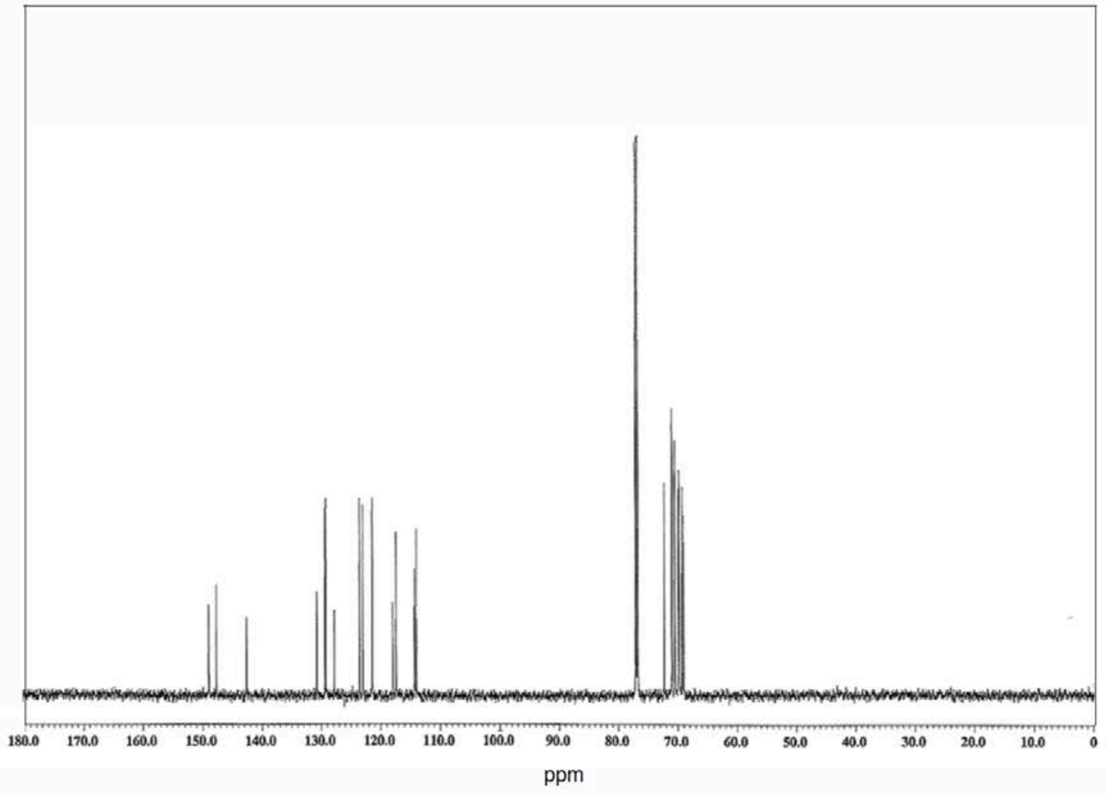

Supplementary Figure 13. ^{13}C NMR spectrum (150 MHz) of **16** in $CDCl_3$.

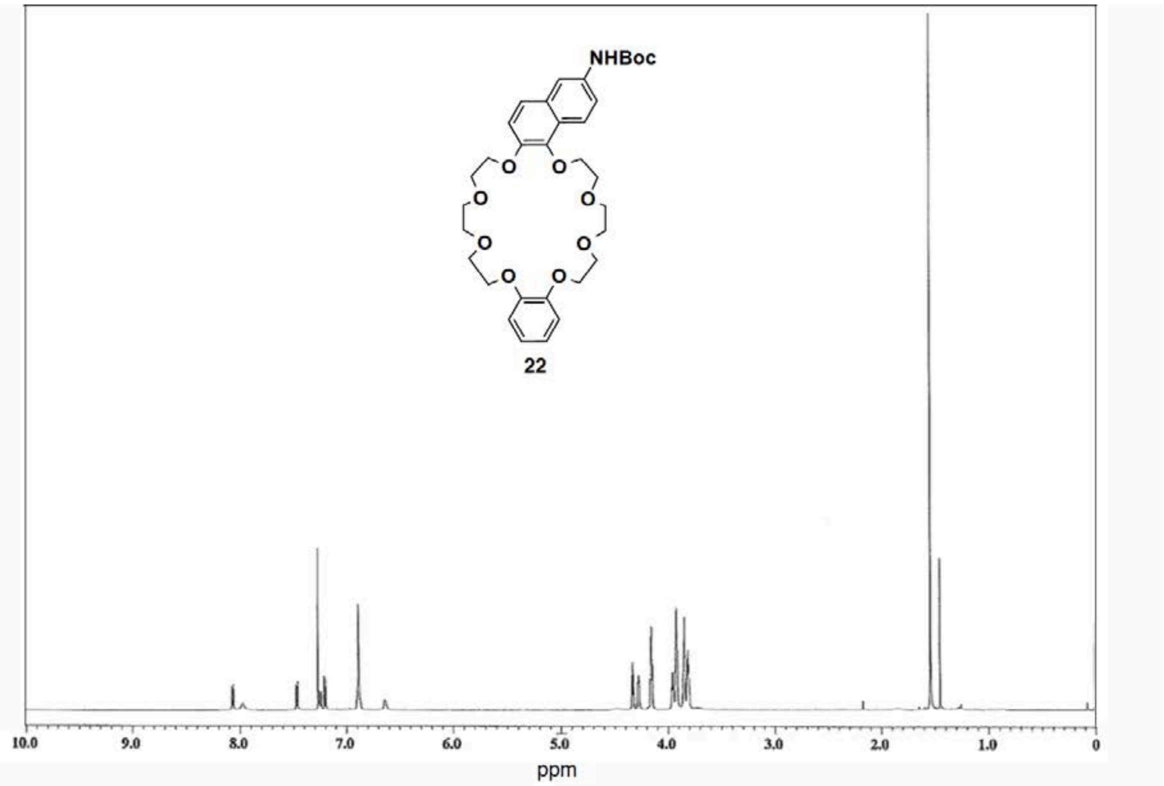

Supplementary Figure 14. ^{1}H NMR spectrum (600 MHz) of **22** in $CDCl_3$.

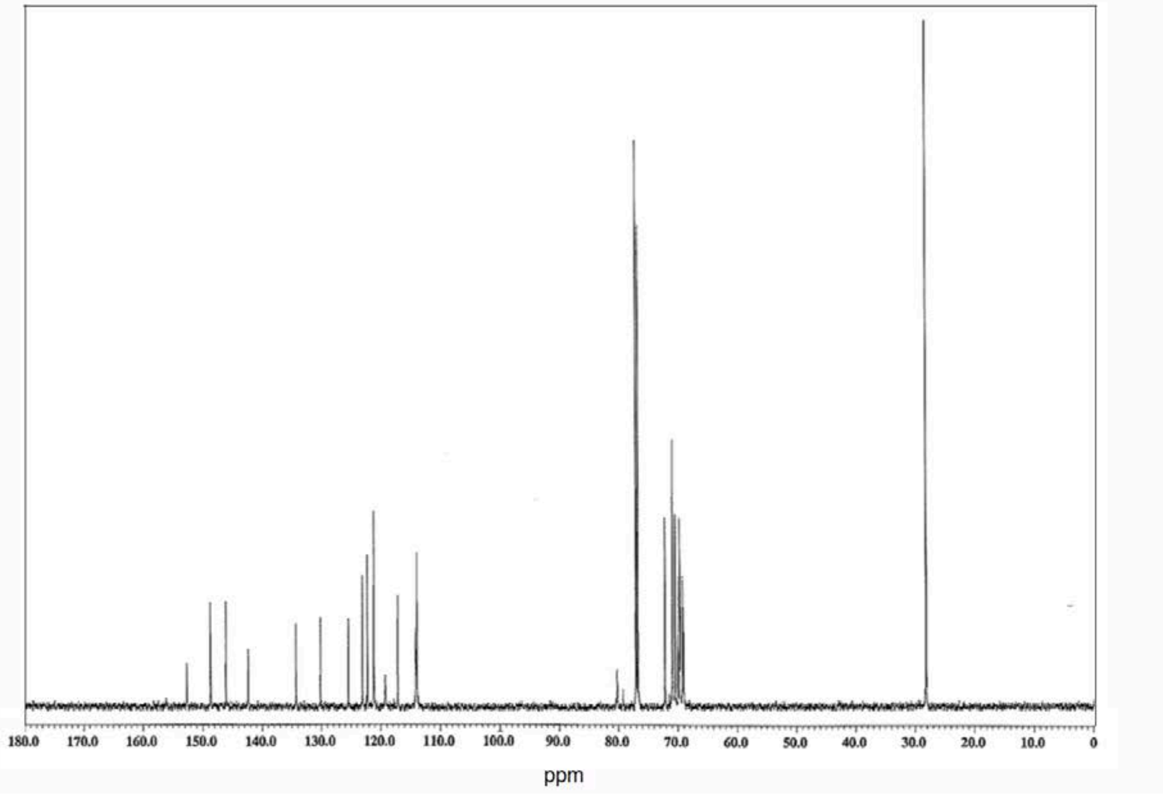

Supplementary Figure 15. ^{13}C NMR spectrum (150 MHz) of **22** in $CDCl_3$.

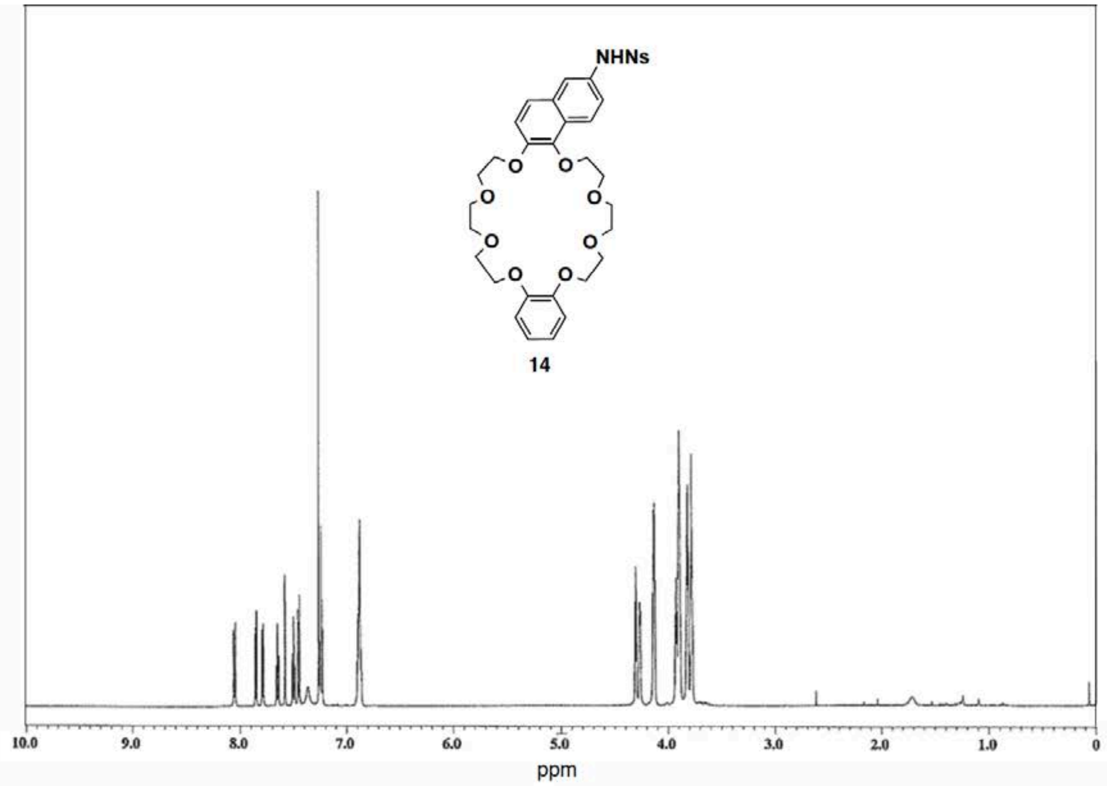

Supplementary Figure 16. ^{1}H NMR spectrum (600 MHz) of **14** in $CDCl_3$.

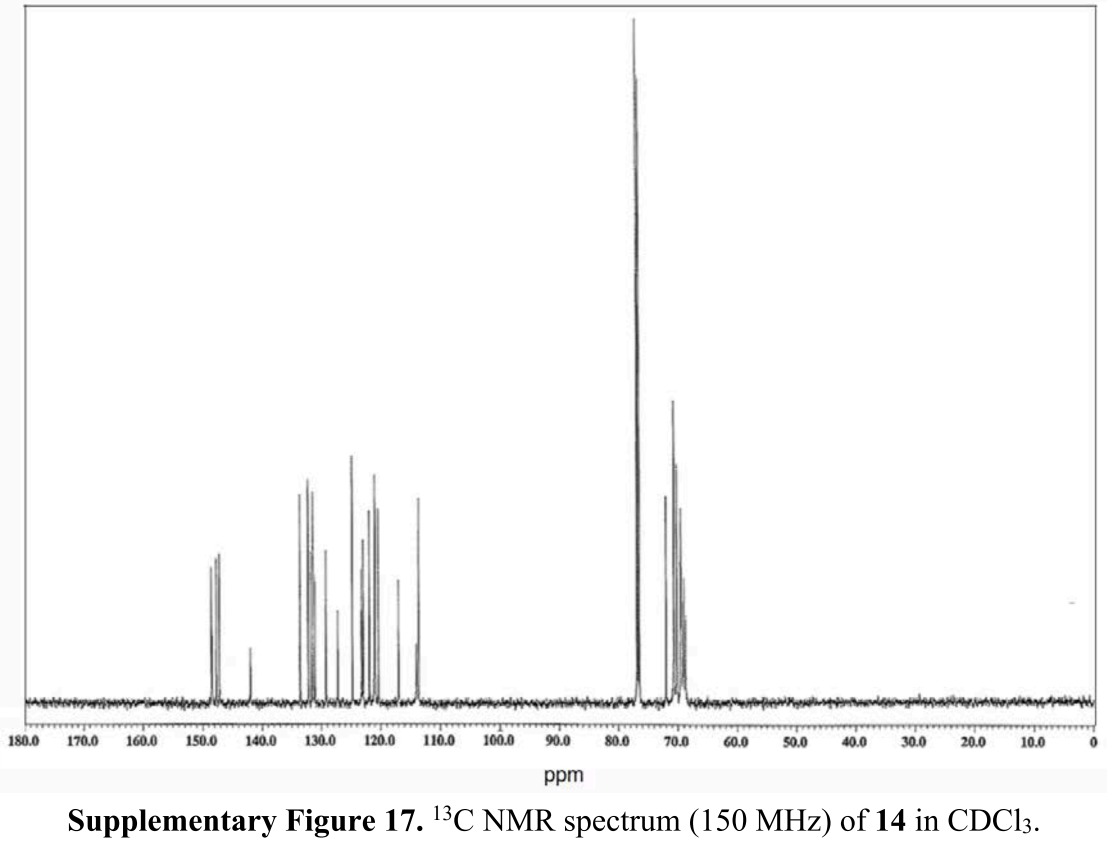

Supplementary Figure 17. ^{13}C NMR spectrum (150 MHz) of **14** in $CDCl_3$.

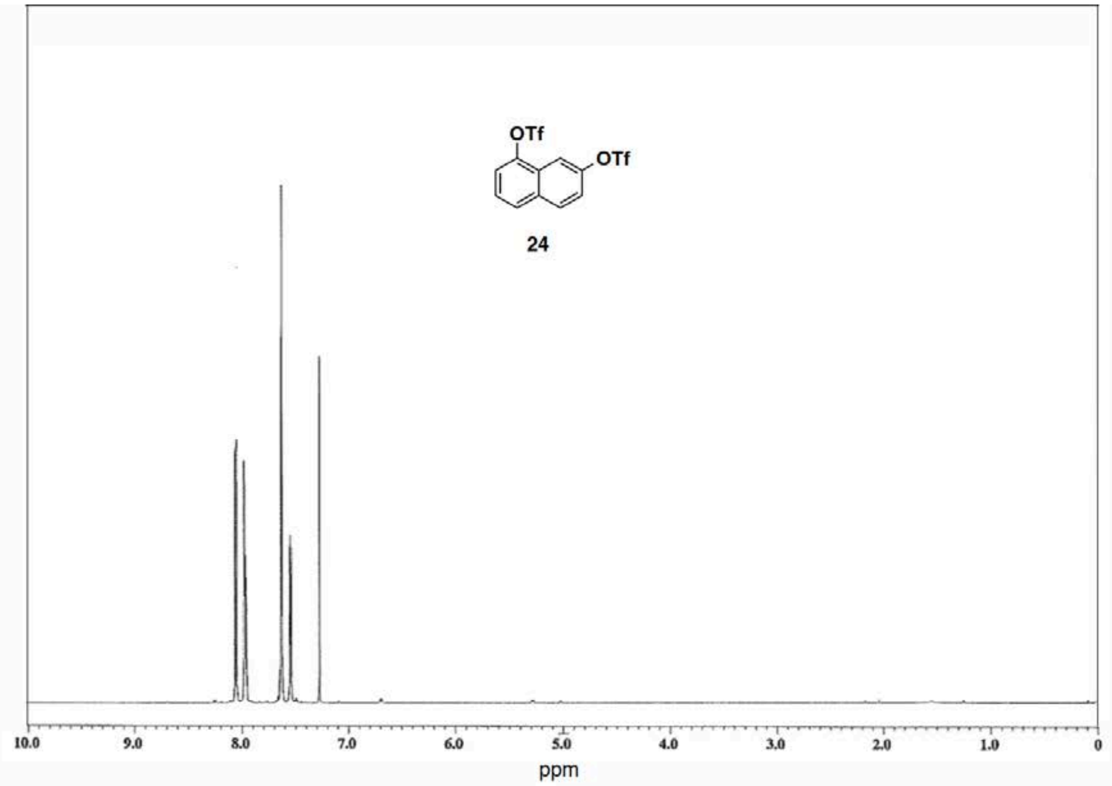

Supplementary Figure 18. ^{1}H NMR spectrum (600 MHz) of **24** in $CDCl_3$.

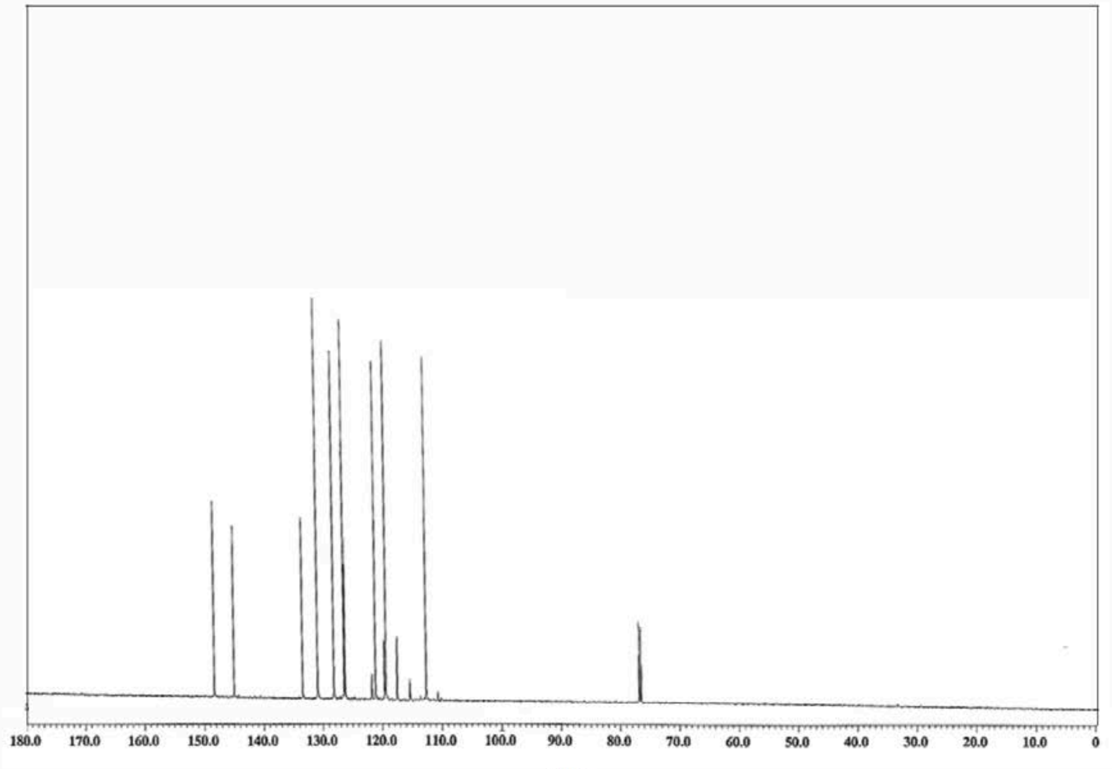

Supplementary Figure 19. ^{13}C NMR spectrum (150 MHz) of **24** in $CDCl_3$.

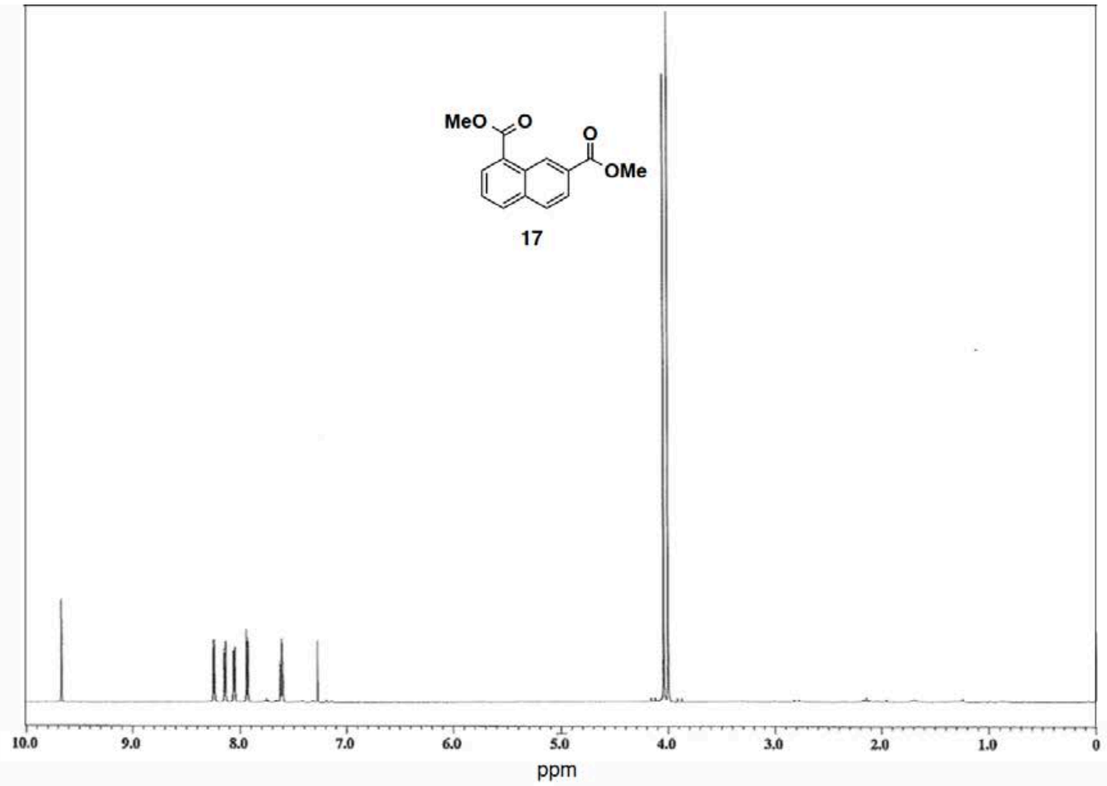

Supplementary Figure 20. ^{1}H NMR spectrum (600 MHz) of **17** in $CDCl_3$.

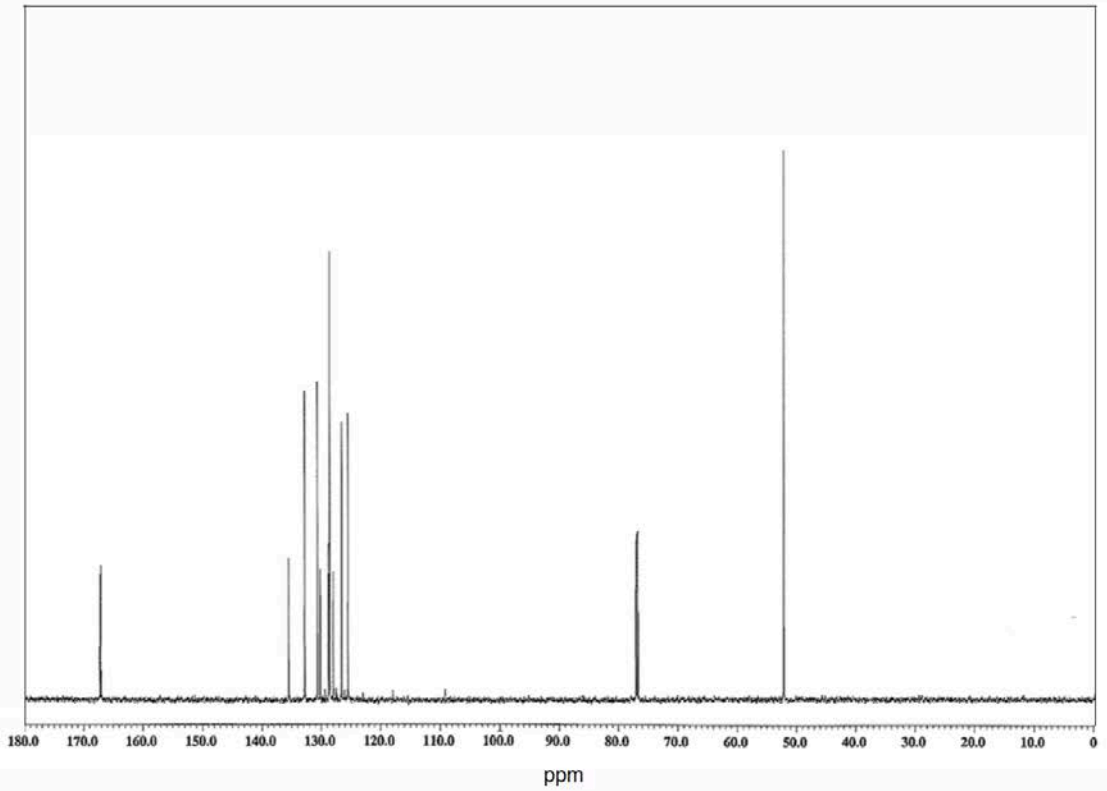

Supplementary Figure 21. ^{13}C NMR spectrum (150 MHz) of **17** in $CDCl_3$.

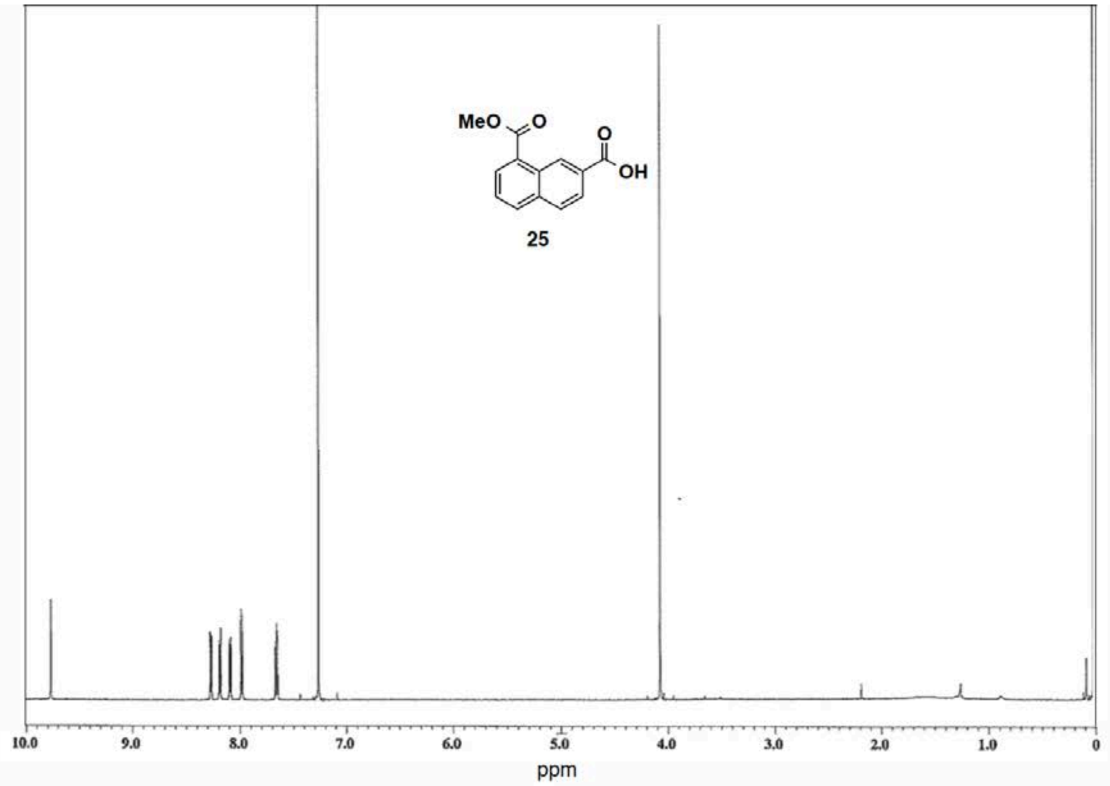

Supplementary Figure 22. ^{1}H NMR spectrum (600 MHz) of **25** in $CDCl_3$.

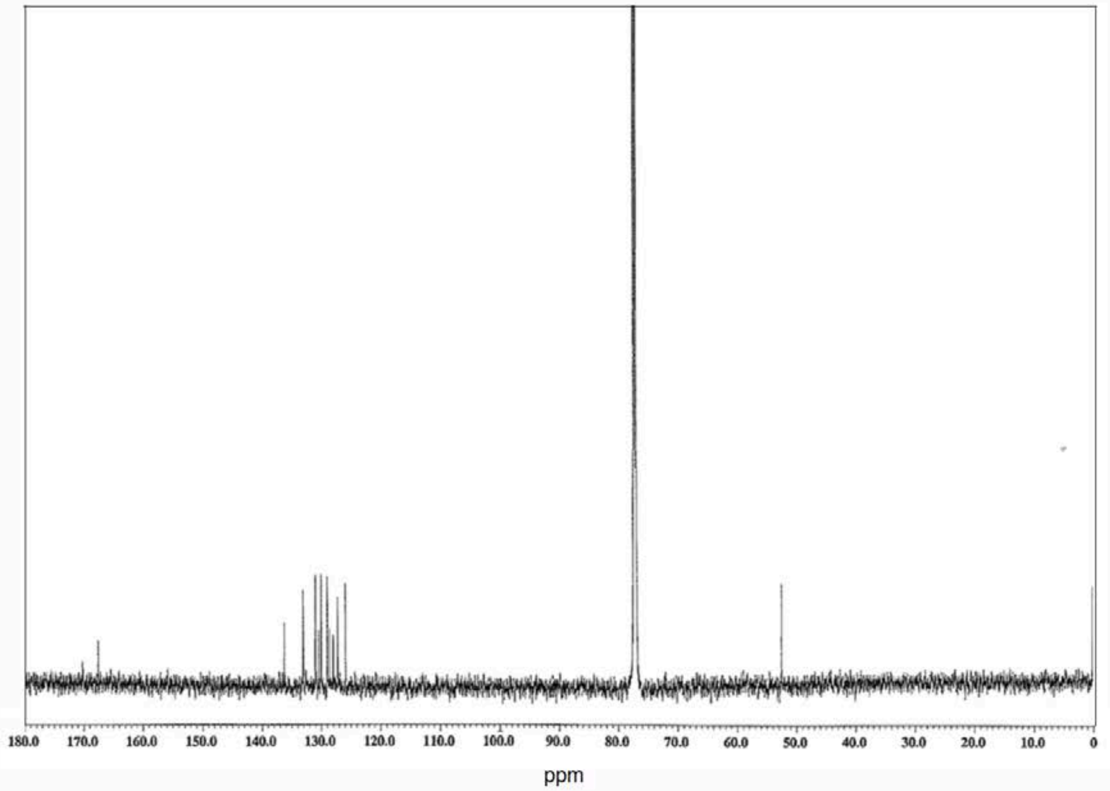

Supplementary Figure 23. ^{13}C NMR spectrum (150 MHz) of **25** in $CDCl_3$.

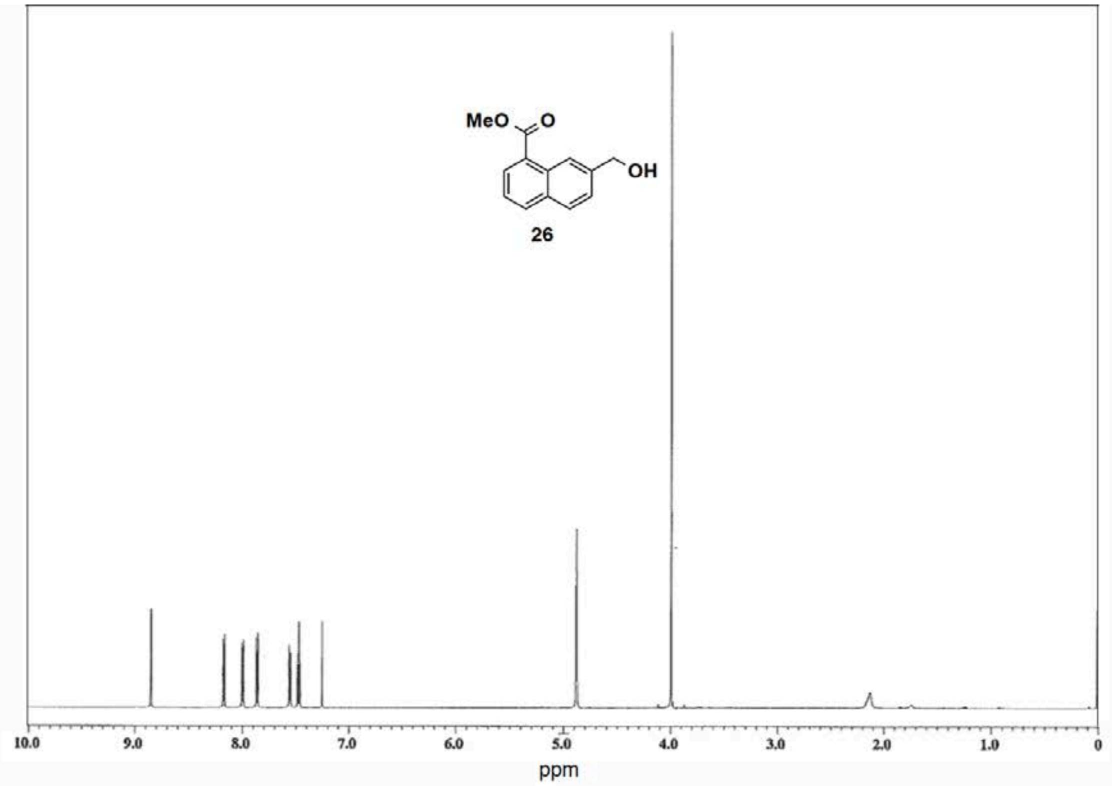

Supplementary Figure 24. ^{1}H NMR spectrum (600 MHz) of **26** in $CDCl_3$.

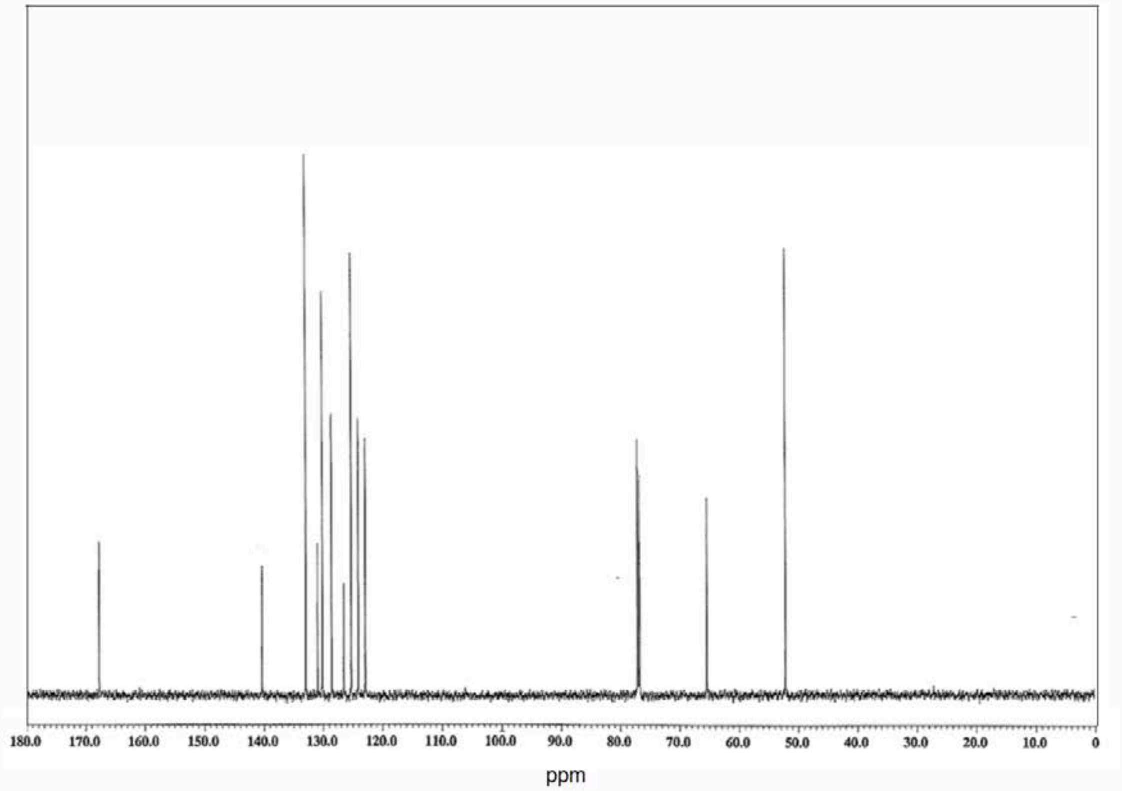

Supplementary Figure 25. ^{13}C NMR spectrum (150 MHz) of **26** in $CDCl_3$.

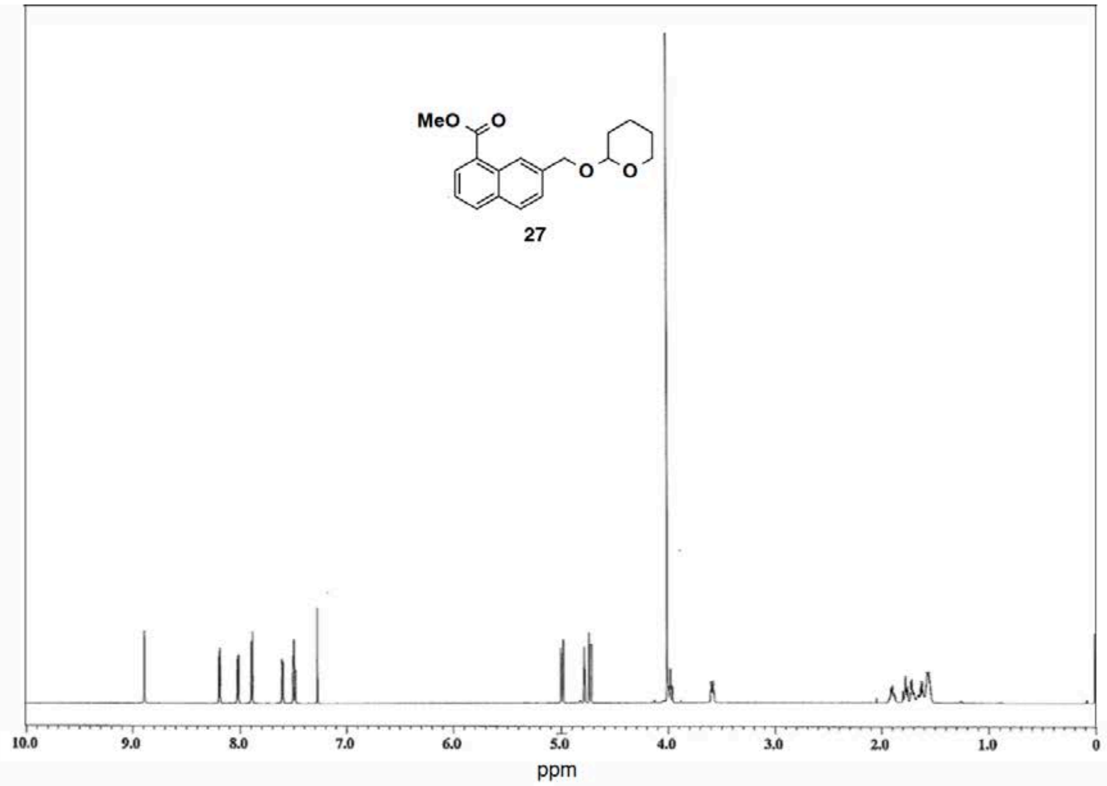

Supplementary Figure 26. ^{1}H NMR spectrum (600 MHz) of **27** in $CDCl_3$.

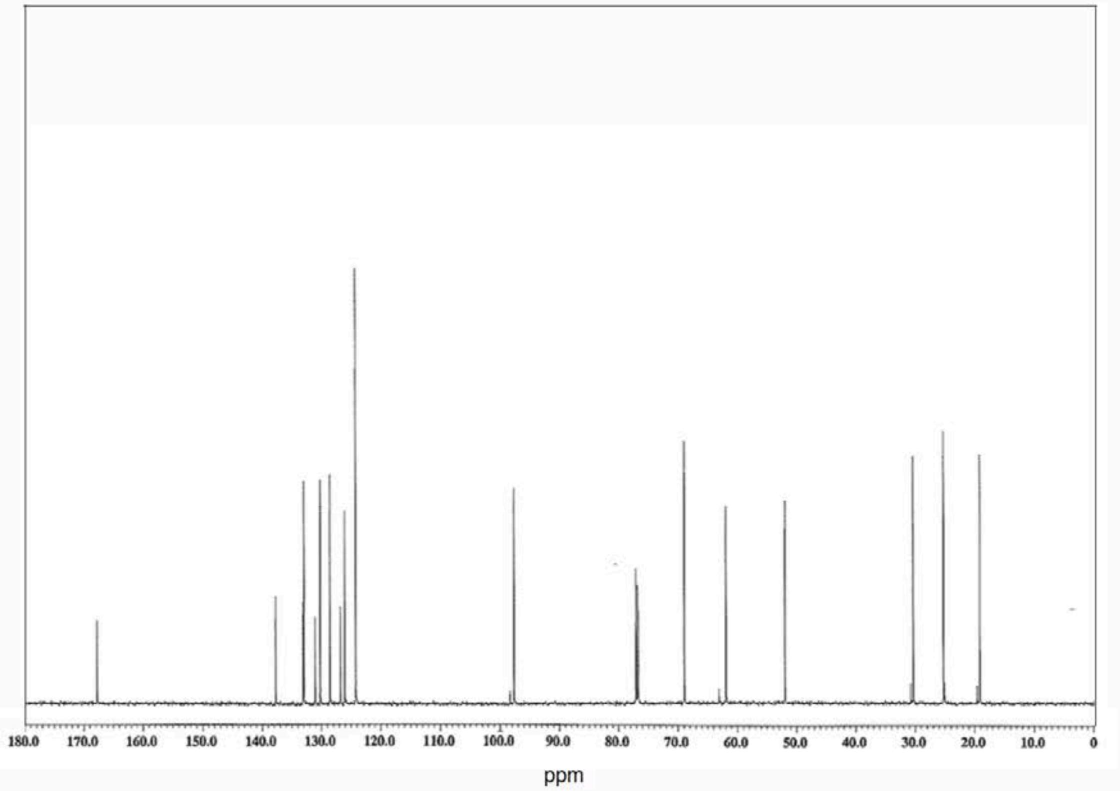

Supplementary Figure 27. ^{13}C NMR spectrum (150 MHz) of **27** in $CDCl_3$.

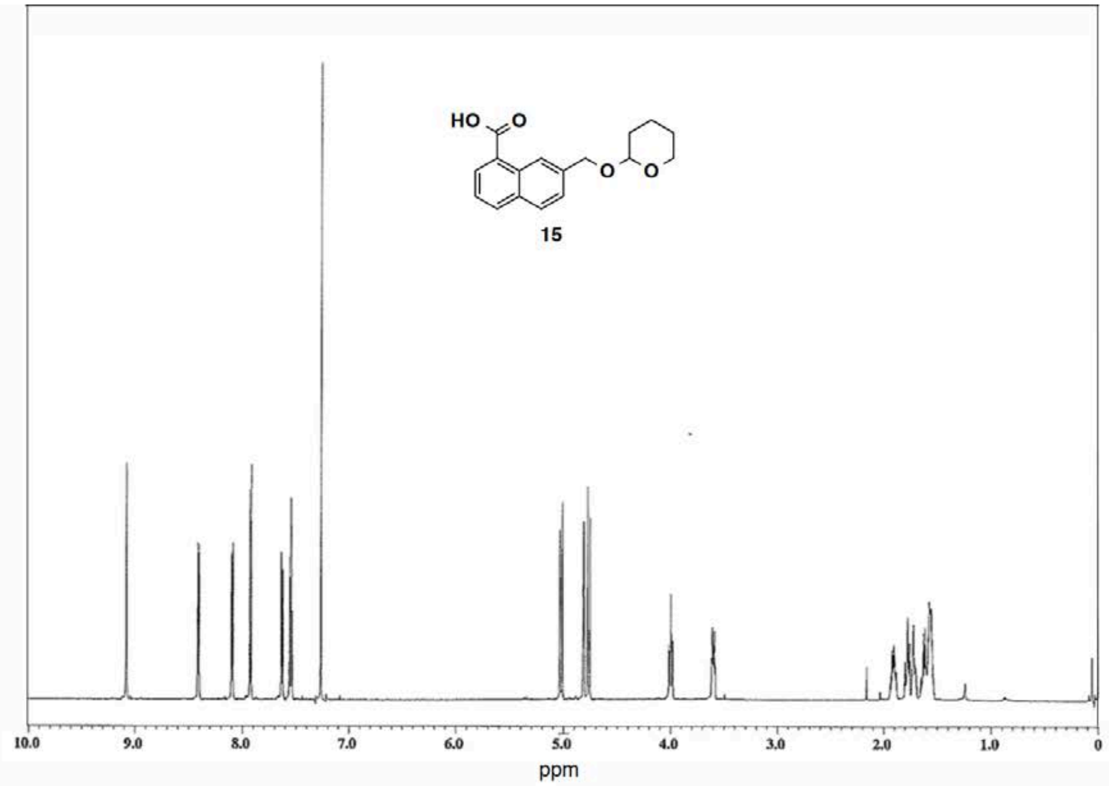

Supplementary Figure 28. ^{1}H NMR spectrum (600 MHz) of **15** in $CDCl_3$.

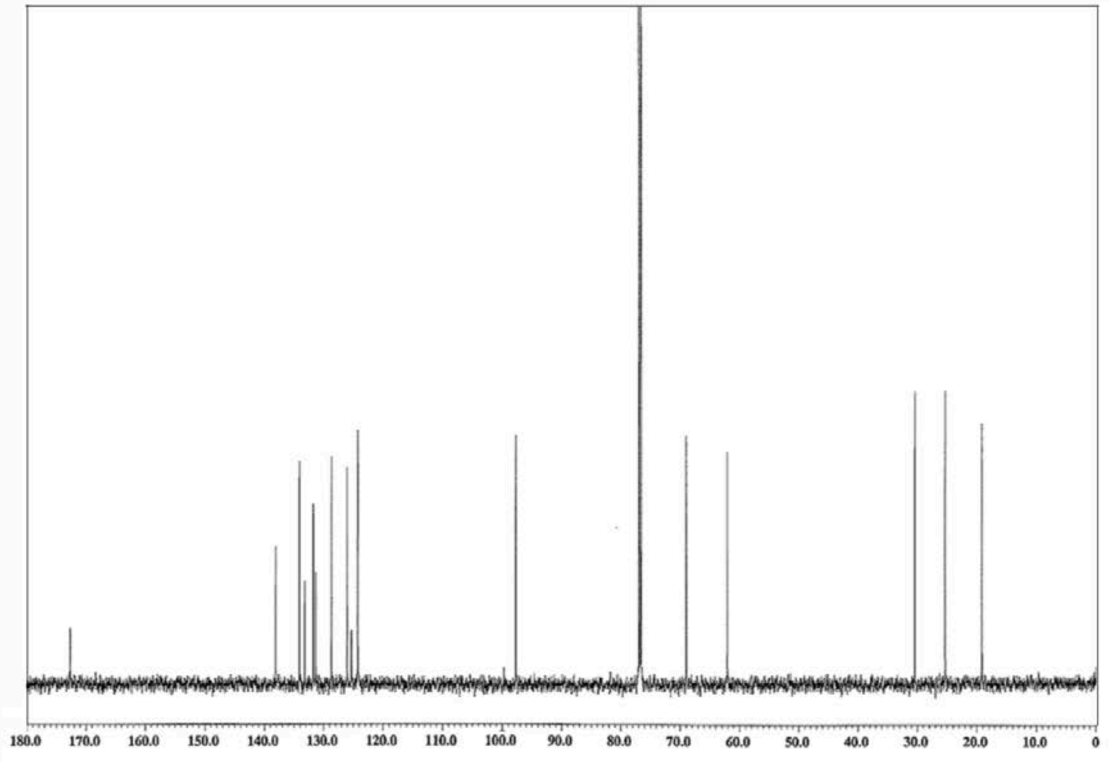

Supplementary Figure 29. ^{13}C NMR spectrum (150 MHz) of **15** in $CDCl_3$.

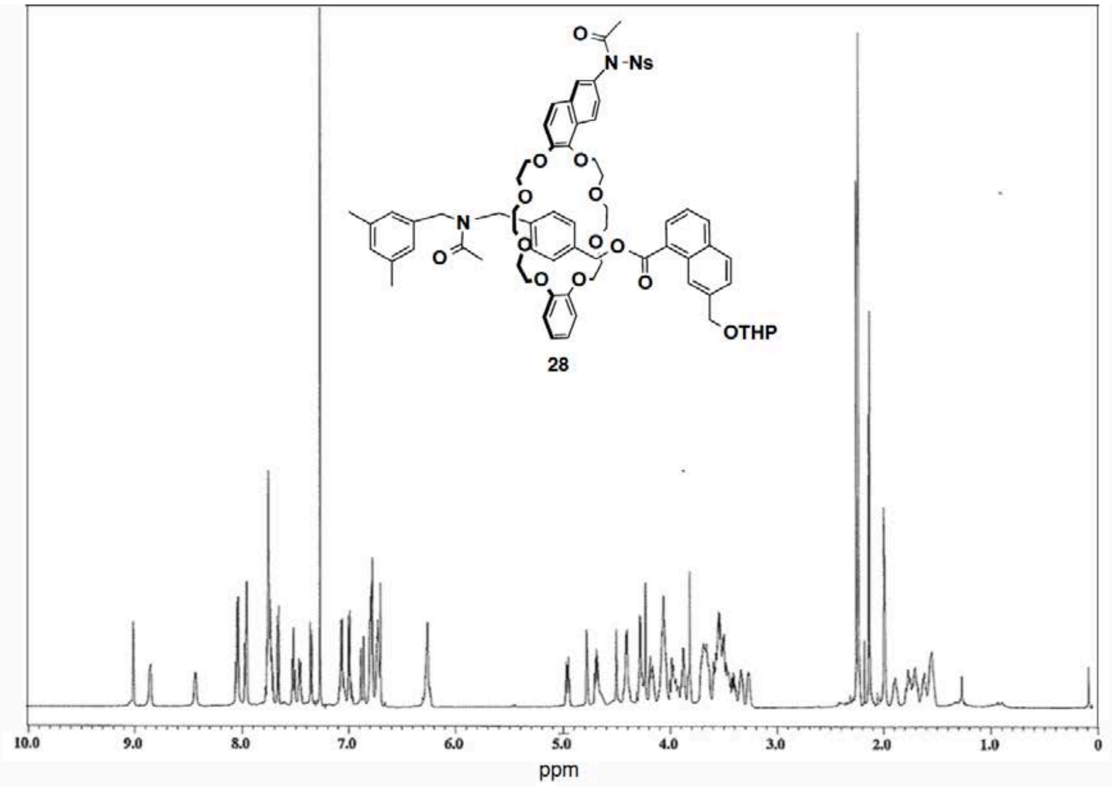

Supplementary Figure 30. ^{1}H NMR spectrum (600 MHz) of **28** in $CDCl_3$.

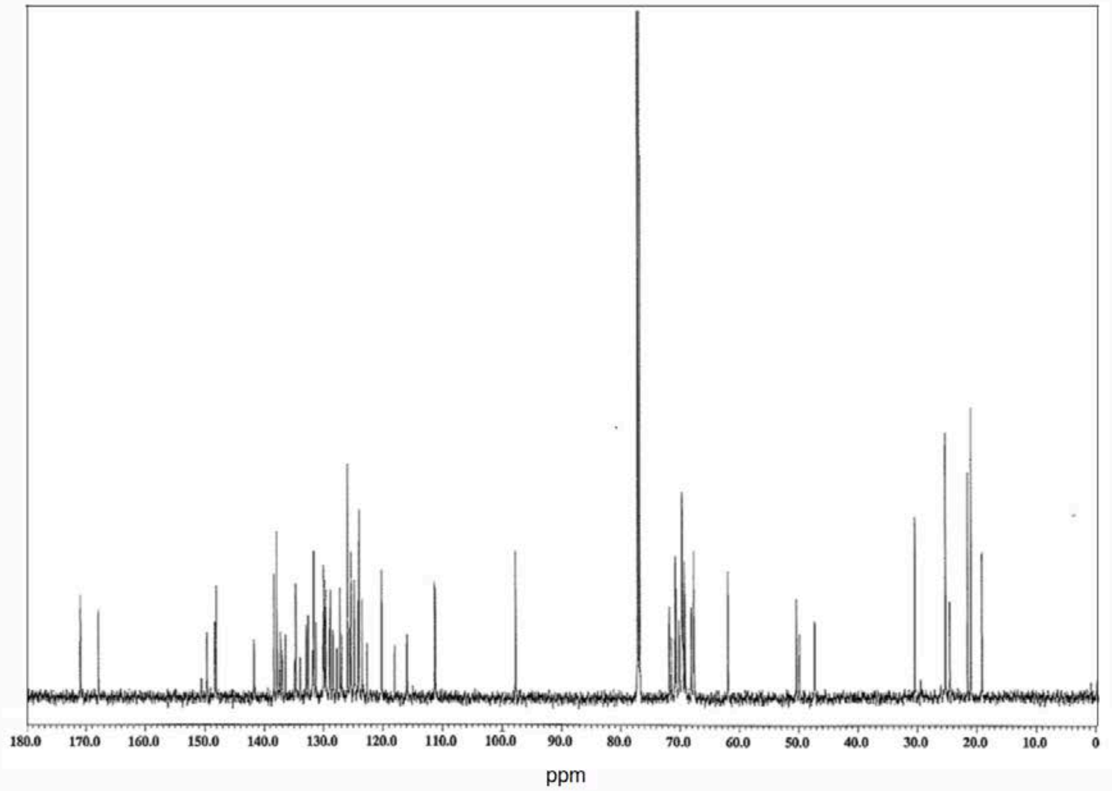

Supplementary Figure 31. ^{13}C NMR spectrum (150 MHz) of **28** in $CDCl_3$.

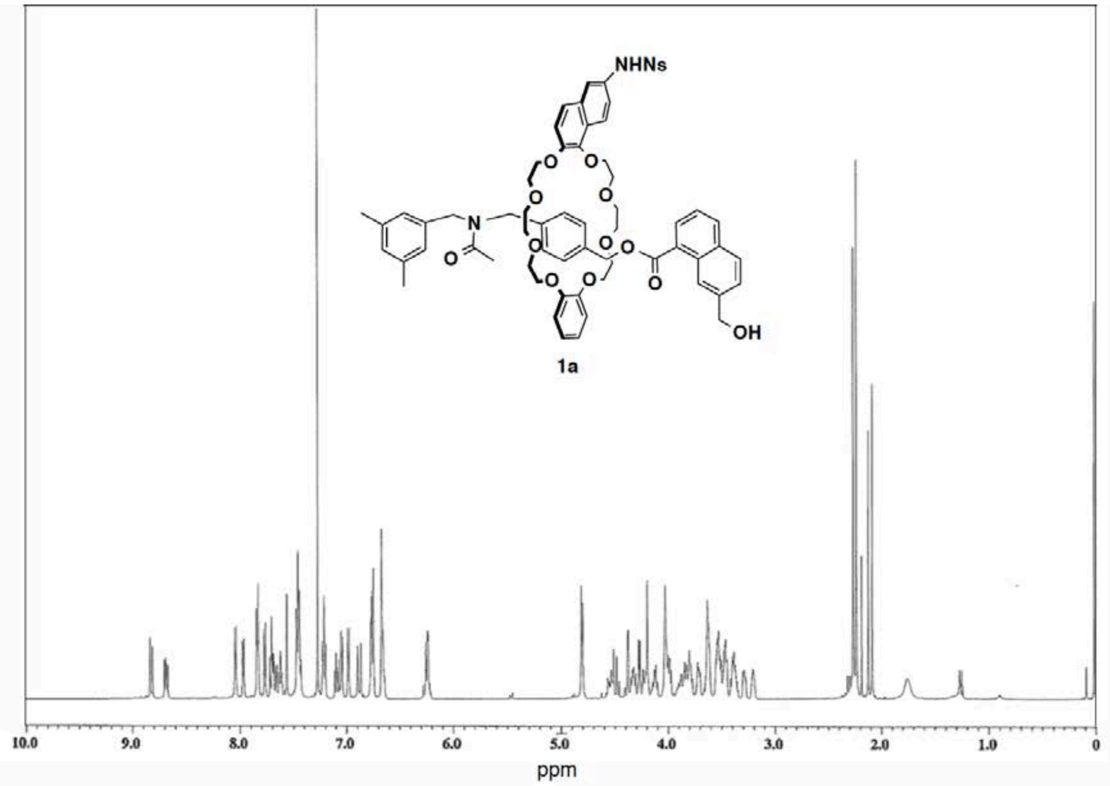

Supplementary Figure 32. ^{1}H NMR spectrum (600 MHz) of **1a** in $CDCl_3$.

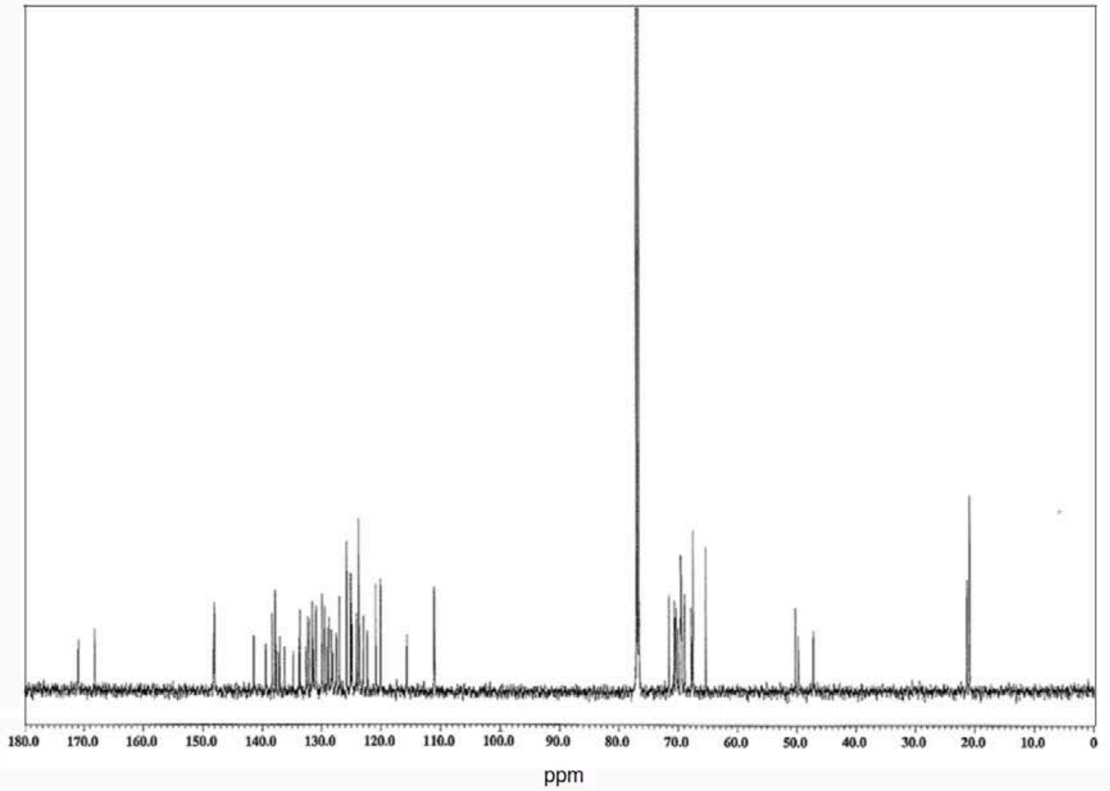

Supplementary Figure 33. ^{13}C NMR spectrum (150 MHz) of **1a** in $CDCl_3$.

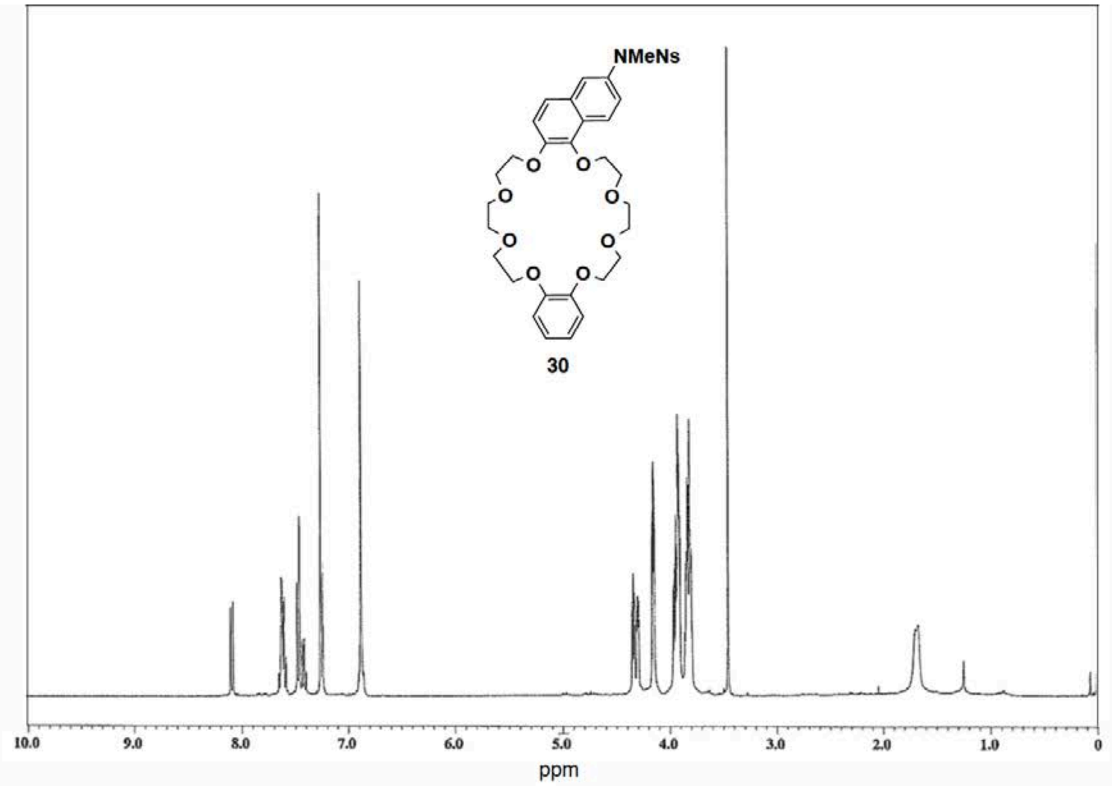

Supplementary Figure 34. ${}^{1}H$ NMR spectrum (400 MHz) of **30** in $CDCl_3$.

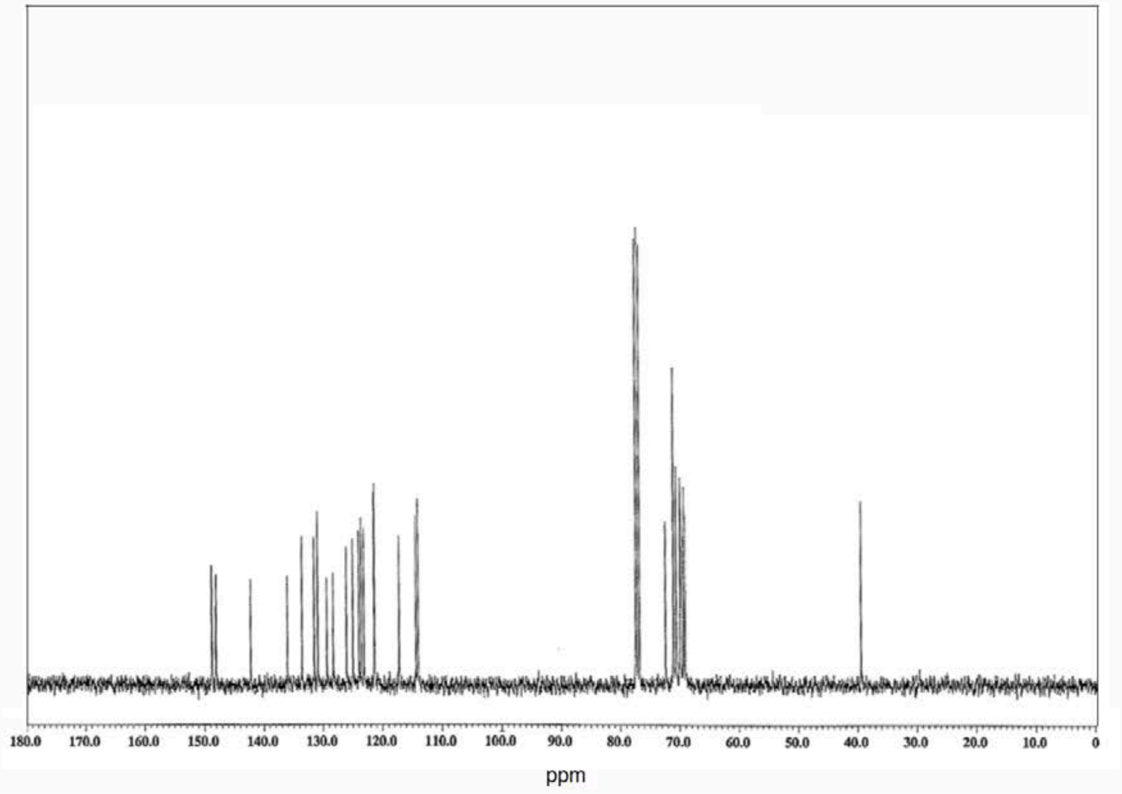

Supplementary Figure 35. ${}^{13}C$ NMR spectrum (100 MHz) of **30** in $CDCl_3$.

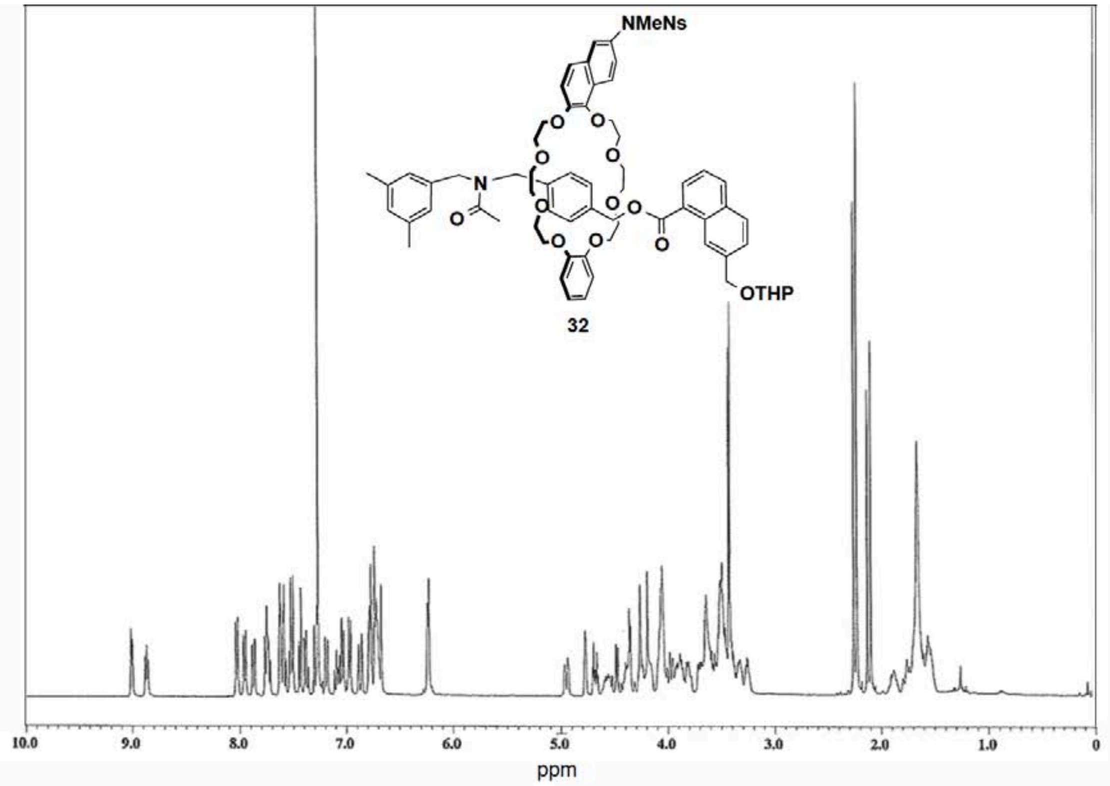

Supplementary Figure 36. ^{1}H NMR spectrum (400 MHz) of **32** in $CDCl_3$.

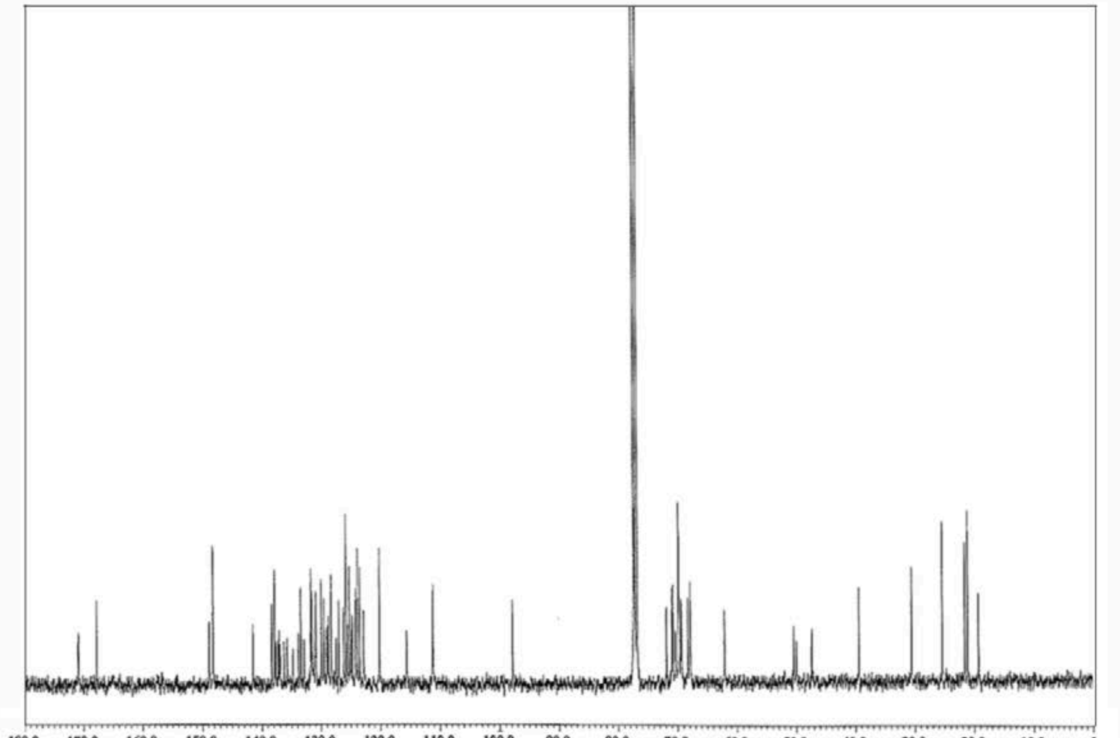

Supplementary Figure 38. ^{13}C NMR spectrum (100 MHz) of **32** in $CDCl_3$.

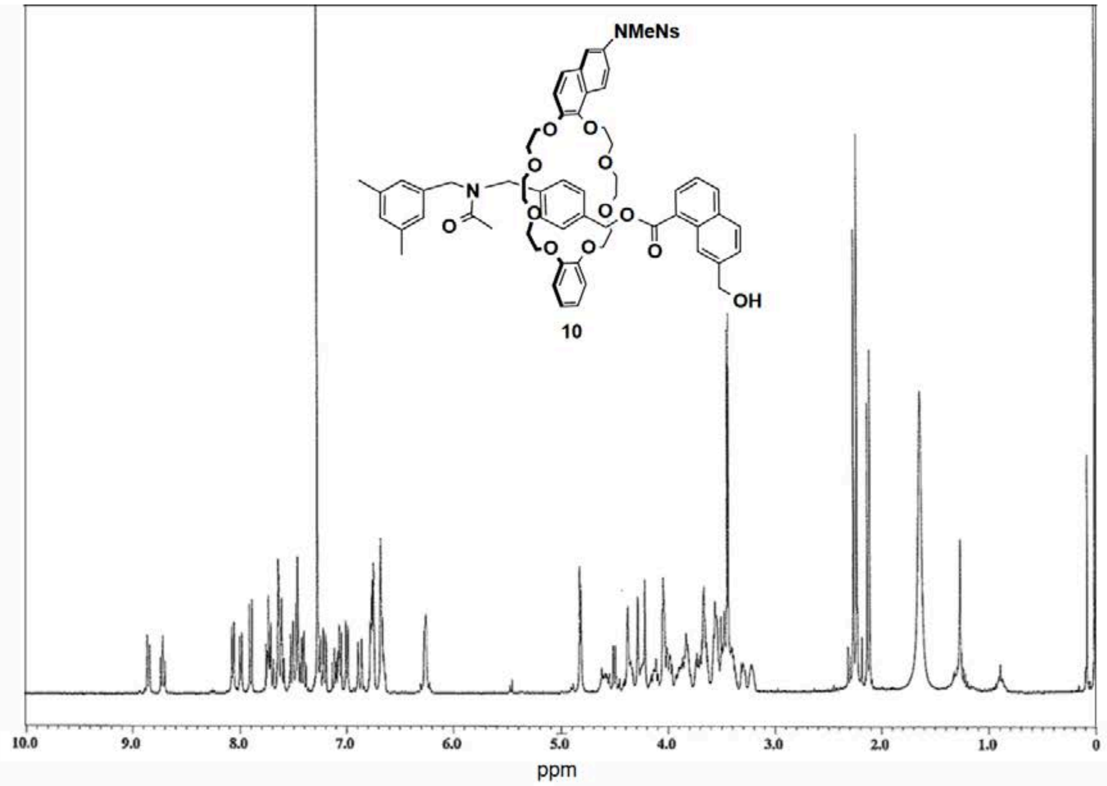

Supplementary Figure 38. ^{1}H NMR spectrum (400 MHz) of **10** in $CDCl_3$.

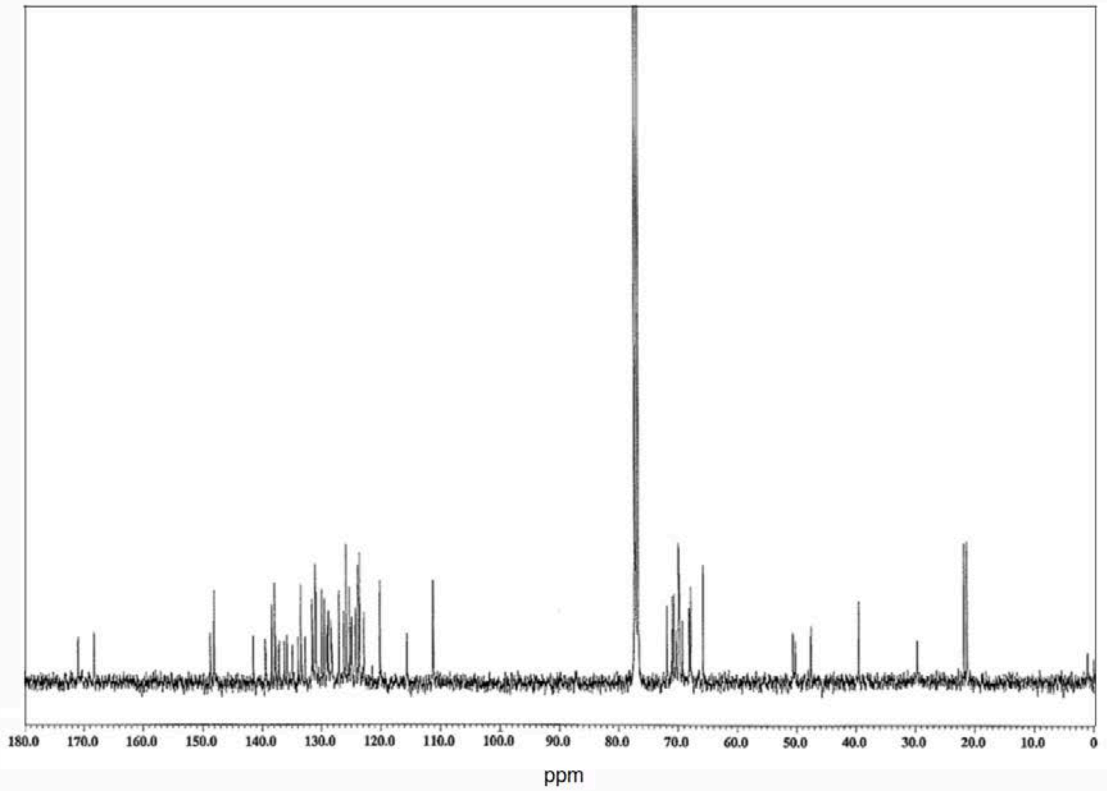

Supplementary Figure 39. ^{13}C NMR spectrum (100 MHz) of **10** in $CDCl_3$.

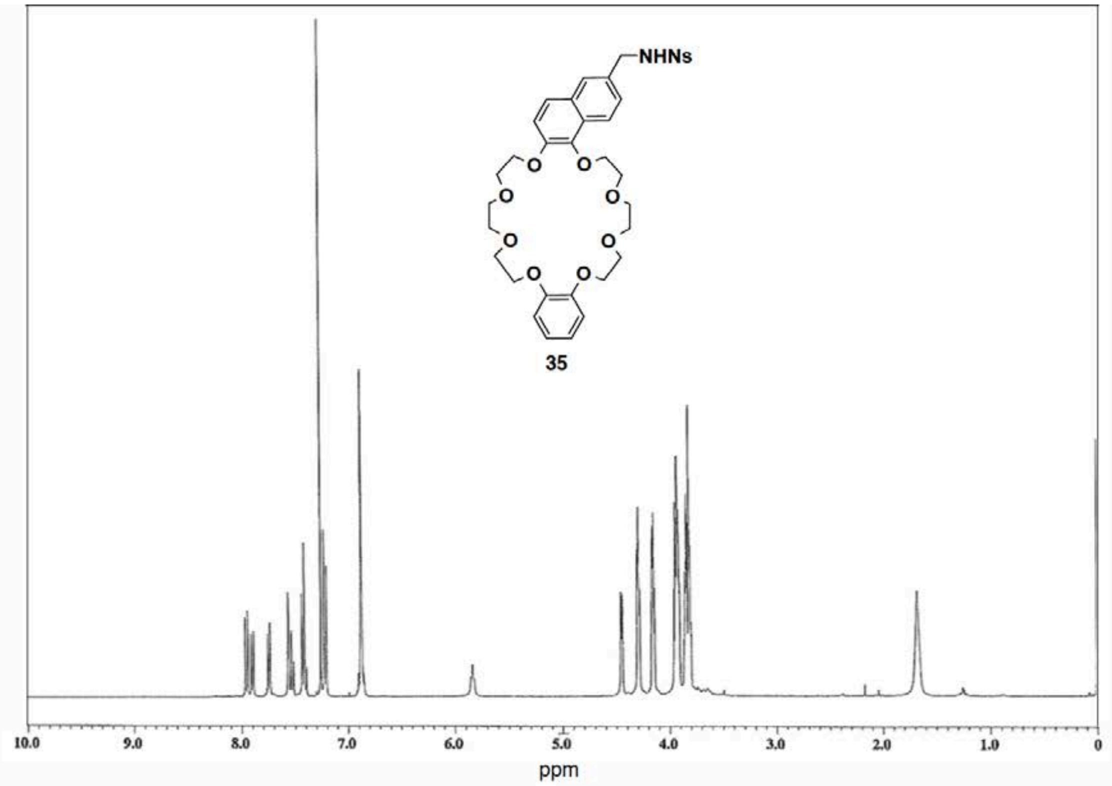

Supplementary Figure 40. ^{1}H NMR spectrum (400 MHz) of **35** in $CDCl_3$.

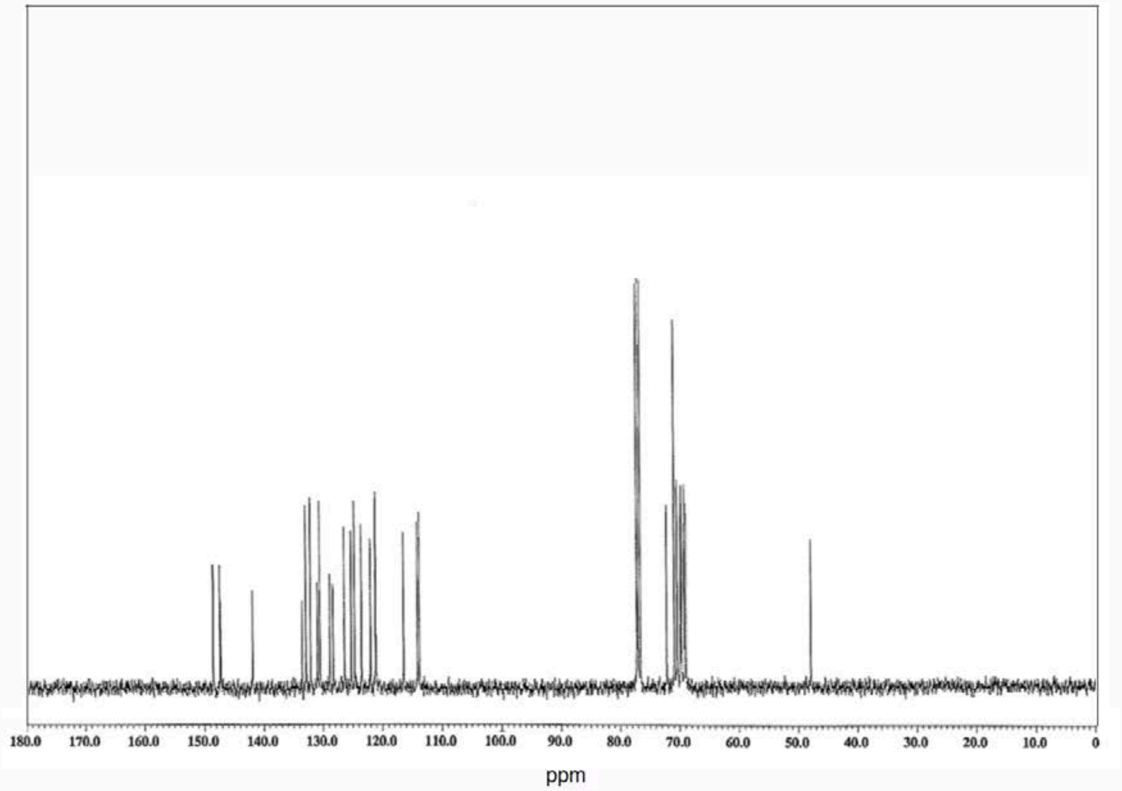

Supplementary Figure 41. ^{13}C NMR spectrum (100 MHz) of **35** in $CDCl_3$.

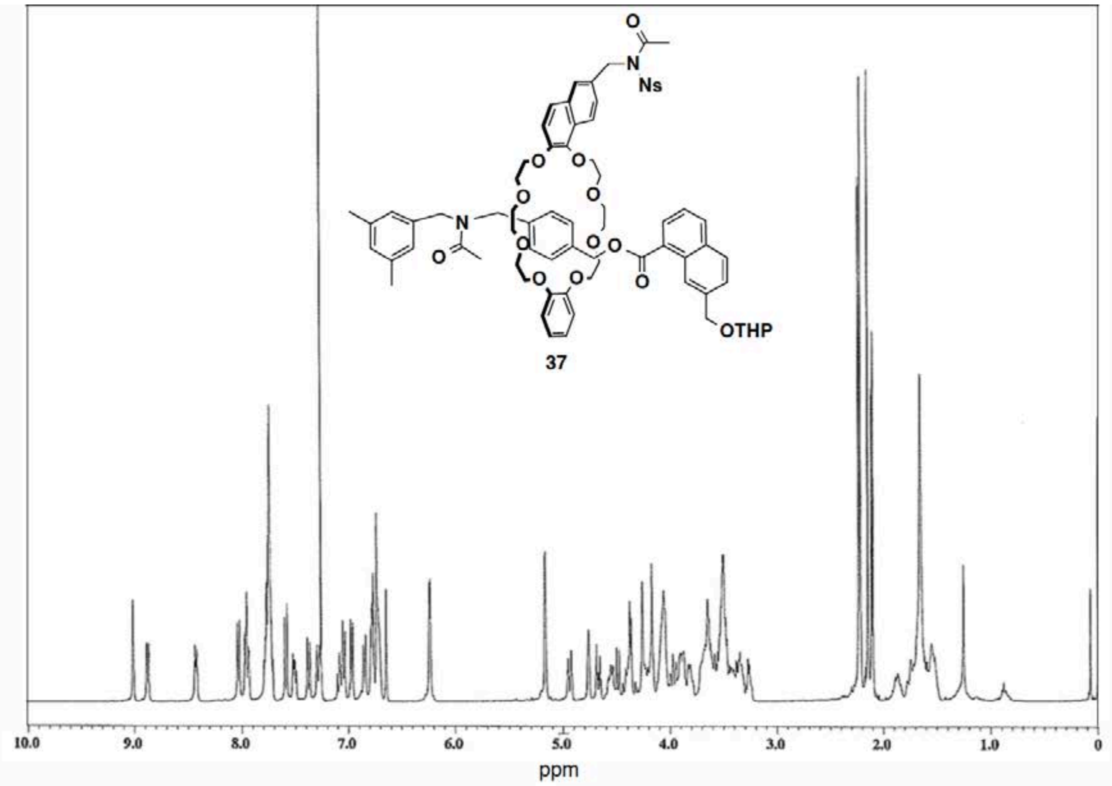

Supplementary Figure 42. ^{1}H NMR spectrum (400 MHz) of **37** in $CDCl_3$.

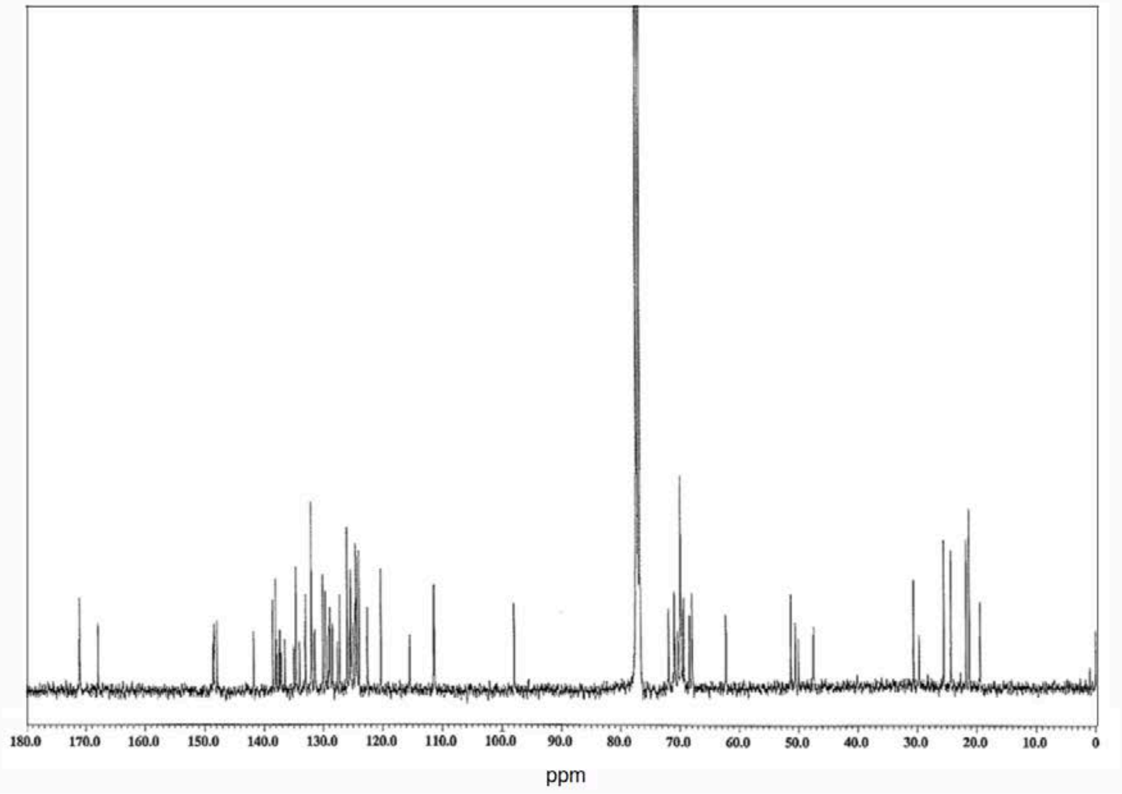

Supplementary Figure 43. ^{13}C NMR spectrum (100 MHz) of **37** in $CDCl_3$.

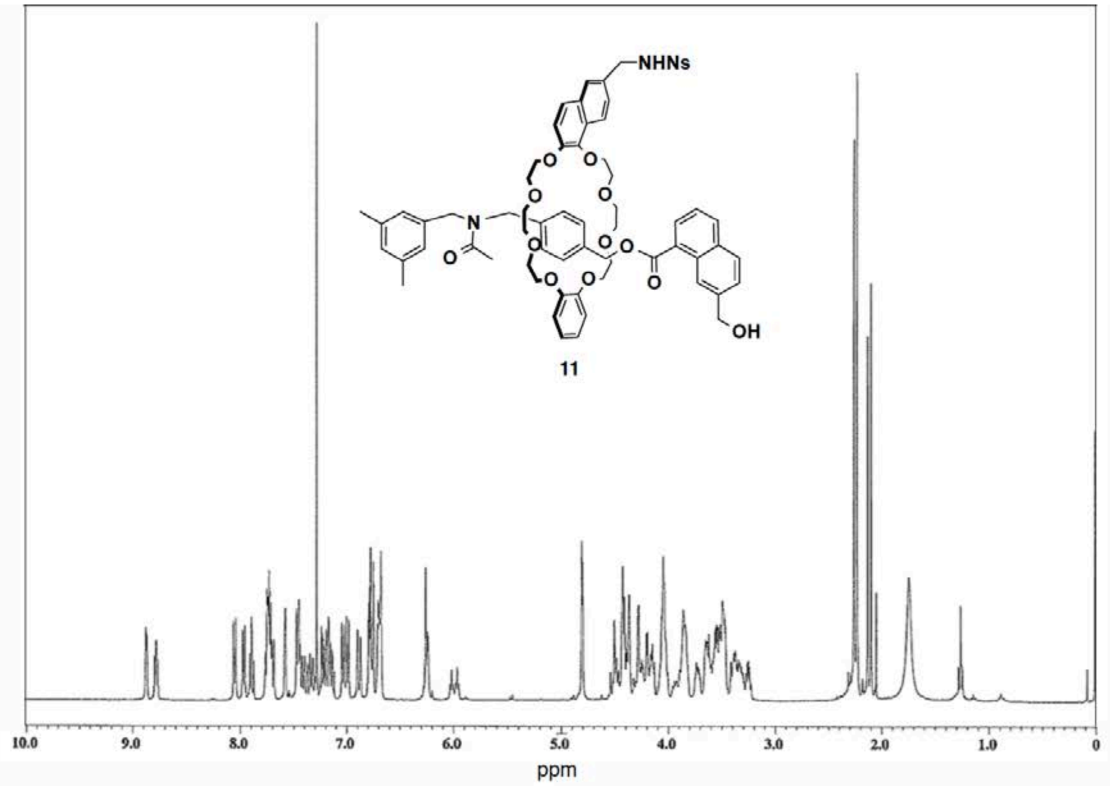

Supplementary Figure 44. ^{1}H NMR spectrum (400 MHz) of **11** in $CDCl_3$.

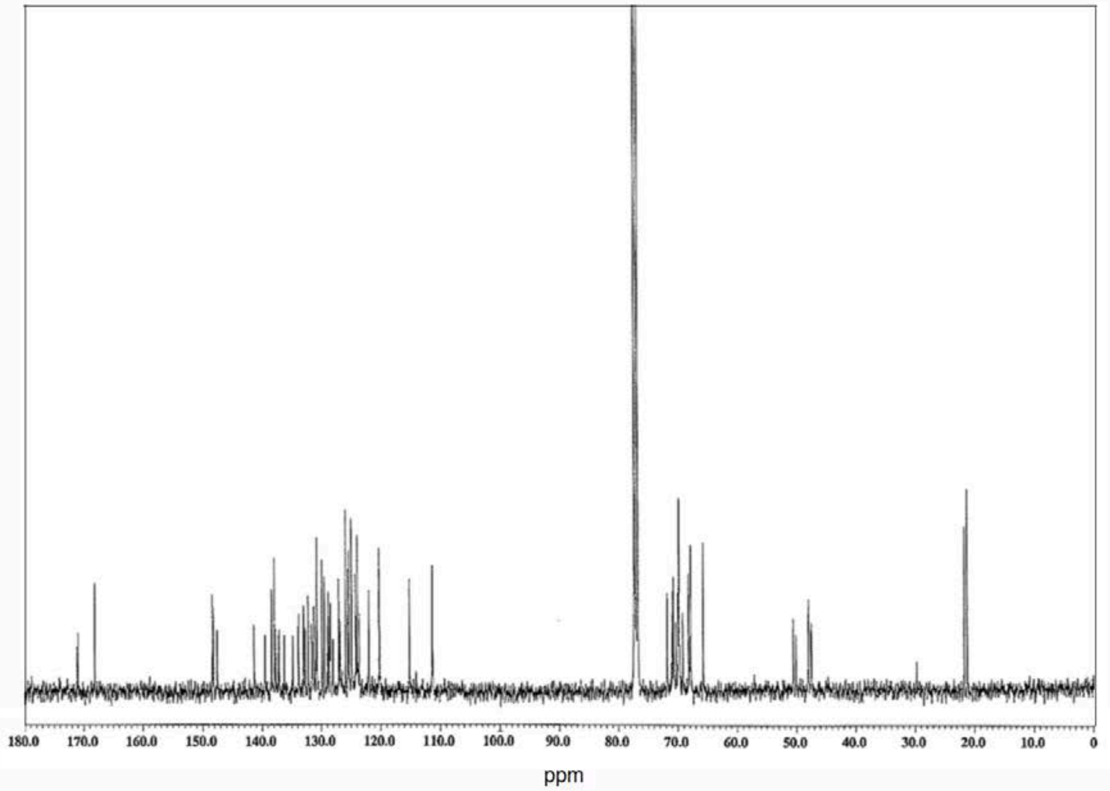

Supplementary Figure 45. ^{13}C NMR spectrum (100 MHz) of **11** in $CDCl_3$.

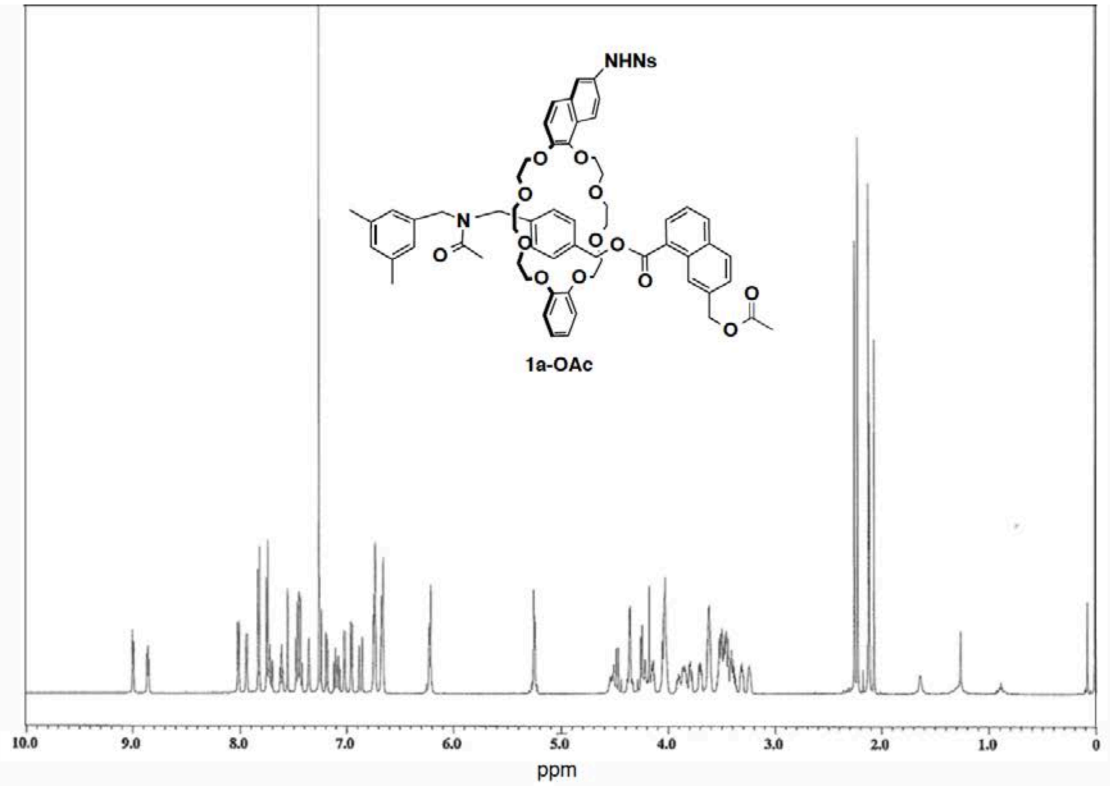

Supplementary Figure 46. ^{1}H NMR spectrum (600 MHz) of **1a-OAc** in $CDCl_3$.

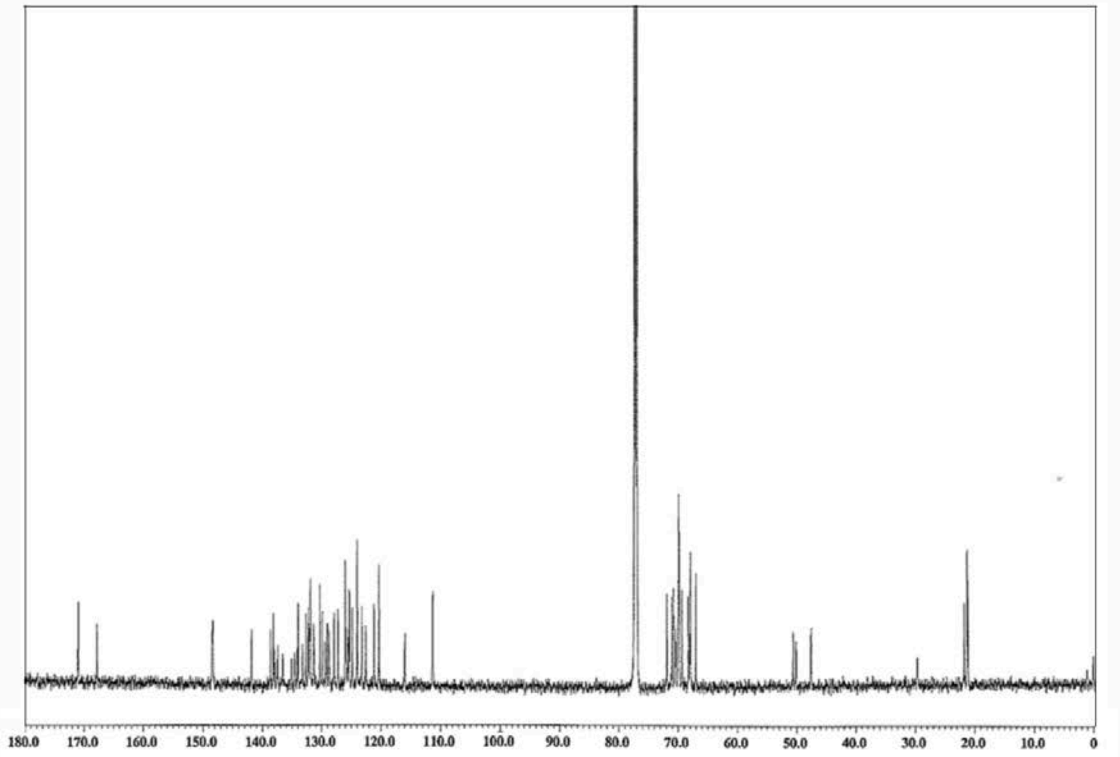

Supplementary Figure 47. ^{13}C NMR spectrum (150 MHz) of **1a-OAc** in $CDCl_3$.

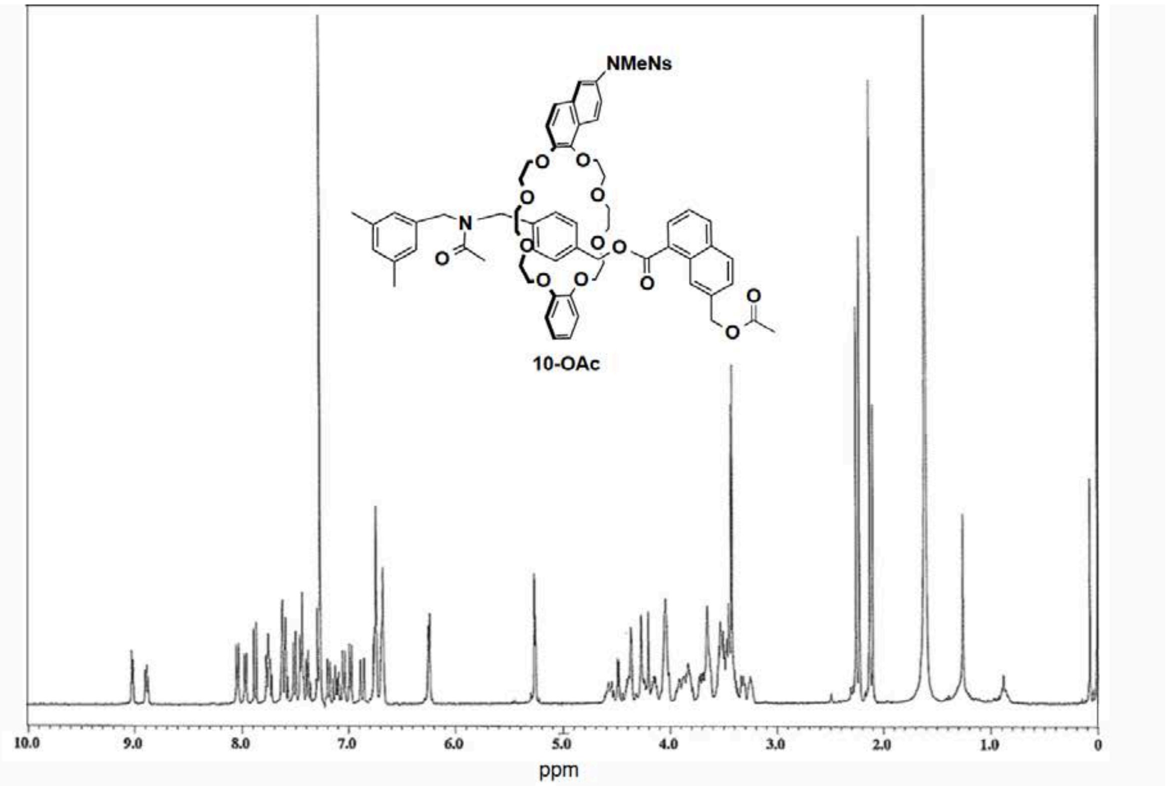

Supplementary Figure 48. ^{1}H NMR spectrum (400 MHz) of **10-OAc** in $CDCl_3$.

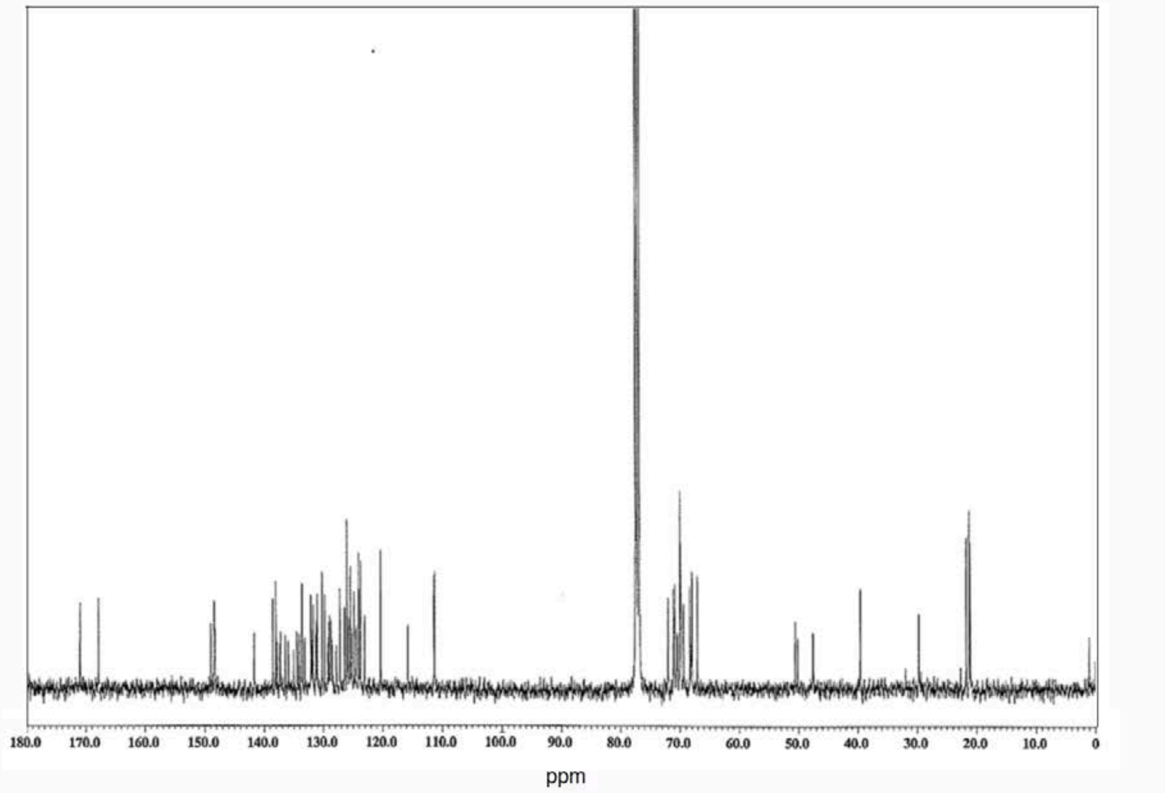

Supplementary Figure 49. ^{13}C NMR spectrum (100 MHz) of **10-OAc** in $CDCl_3$.

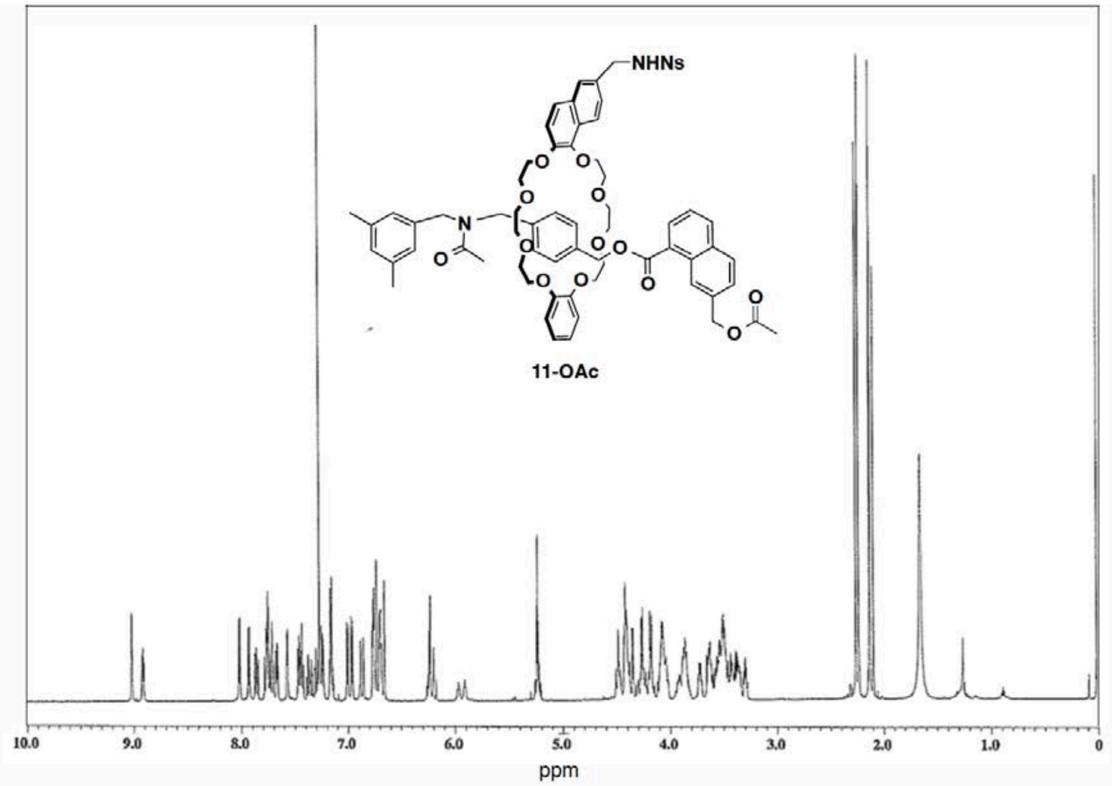

Supplementary Figure 50. ^{1}H NMR spectrum (600 MHz) of **11-OAc** in $CDCl_3$.

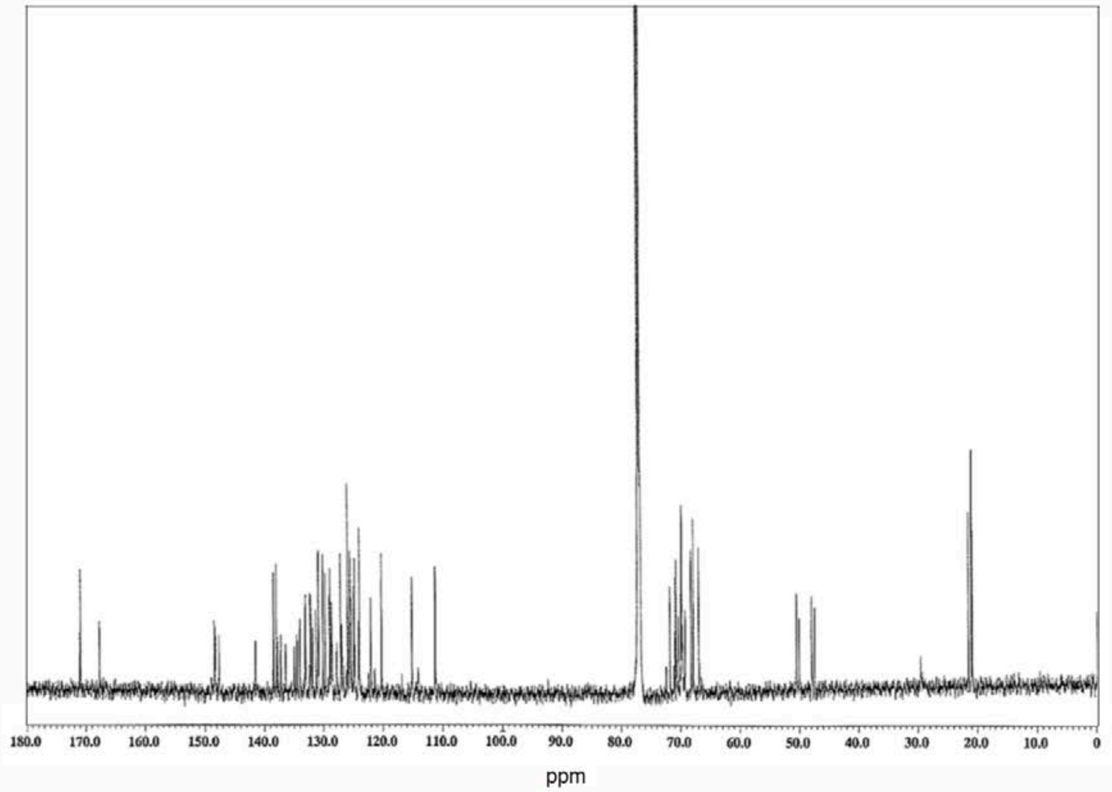

Supplementary Figure 51. ^{13}C NMR spectrum (150 MHz) of **11-OAc** in $CDCl_3$.

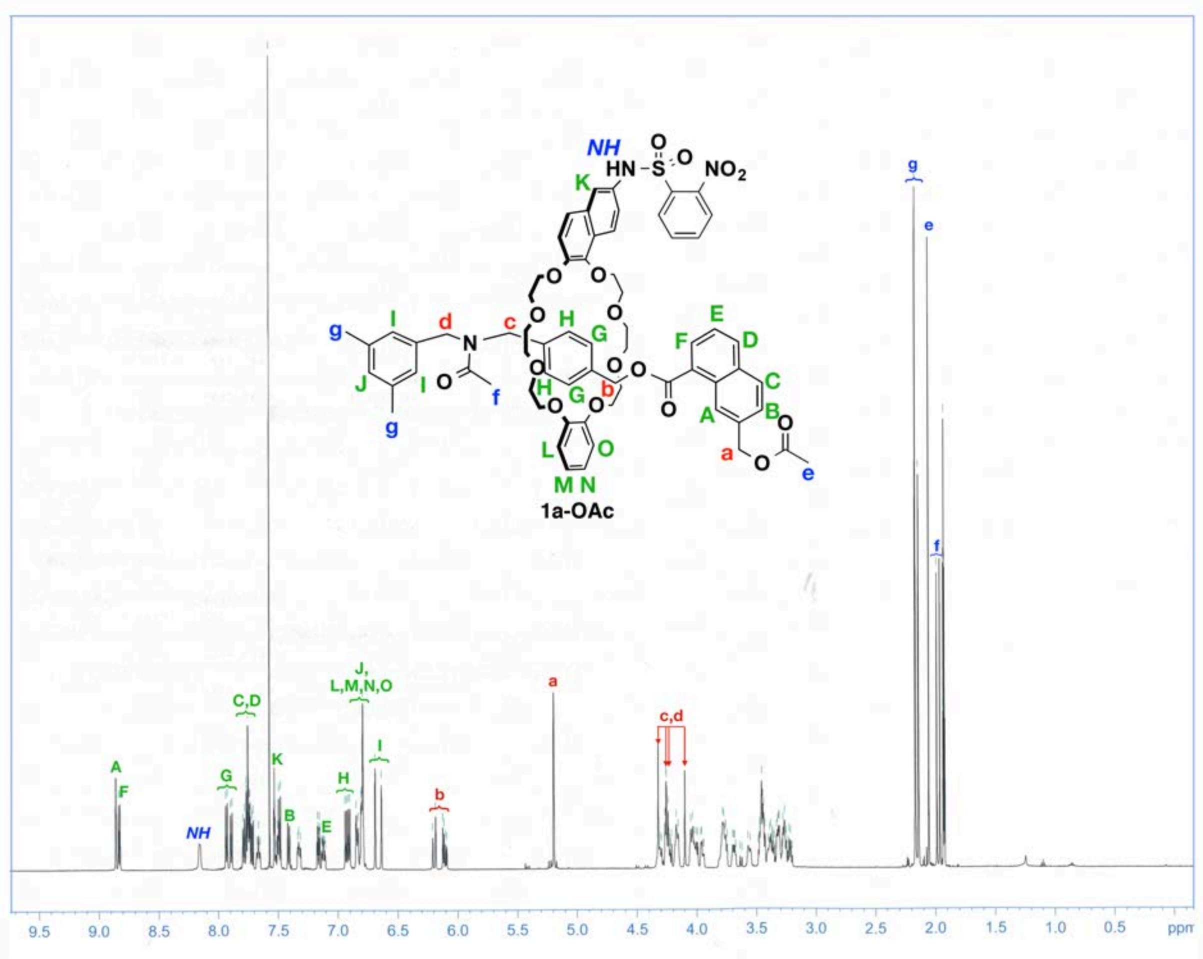

^{1}H NMR (600 MHz, CD_3CN, 20 °C): δ 8.86 (s, 1H, A), 8.84–8.83 (m, 1H, F), 8.16 (brs, 1H, *NH*), 7.94–7.89 (m, 2H, G), 7.80–7.71 (m, 5H, C+D+3H), 7.68–7.65 (m, 1H), 7.54–7.53 (m, 1H, K), 7.52–7.48 (m, 2H), 7.42–7.40 (m, 1H, B), 7.34–7.32 (m, 1H), 7.18–7.15 (m, 1H), 7.14–7.12 (m, 1H, E), 6.94–6.90 (m, 2H, H), 6.85–6.79 (m, 5H, J+L+M+N+O), 6.69–6.64 (m, 2H, I), 6.21–6.19 (m, 1H, b), 6.13–6.09 (m, 1H, b), 5.20 (s, 2H, a), 4.34–4.10 (m, 8H, c+d+4H), 4.06–3.94 (m, 4H), 3.82–3.75 (m, 3H), 3.71–3.68 (m, 1H), 3.57–3.55 (m, 1H), 3.48–3.20 (m, 11H), 2.17–2.14 (m, 6H, g), 2.06 (s, 3H, e), 1.99–1.96 (m, 3H, f).

Supplementary Figure 52. ^{1}H NMR spectrum (600 MHz) of **1a-OAc** in CD_3CN at 20 °C.

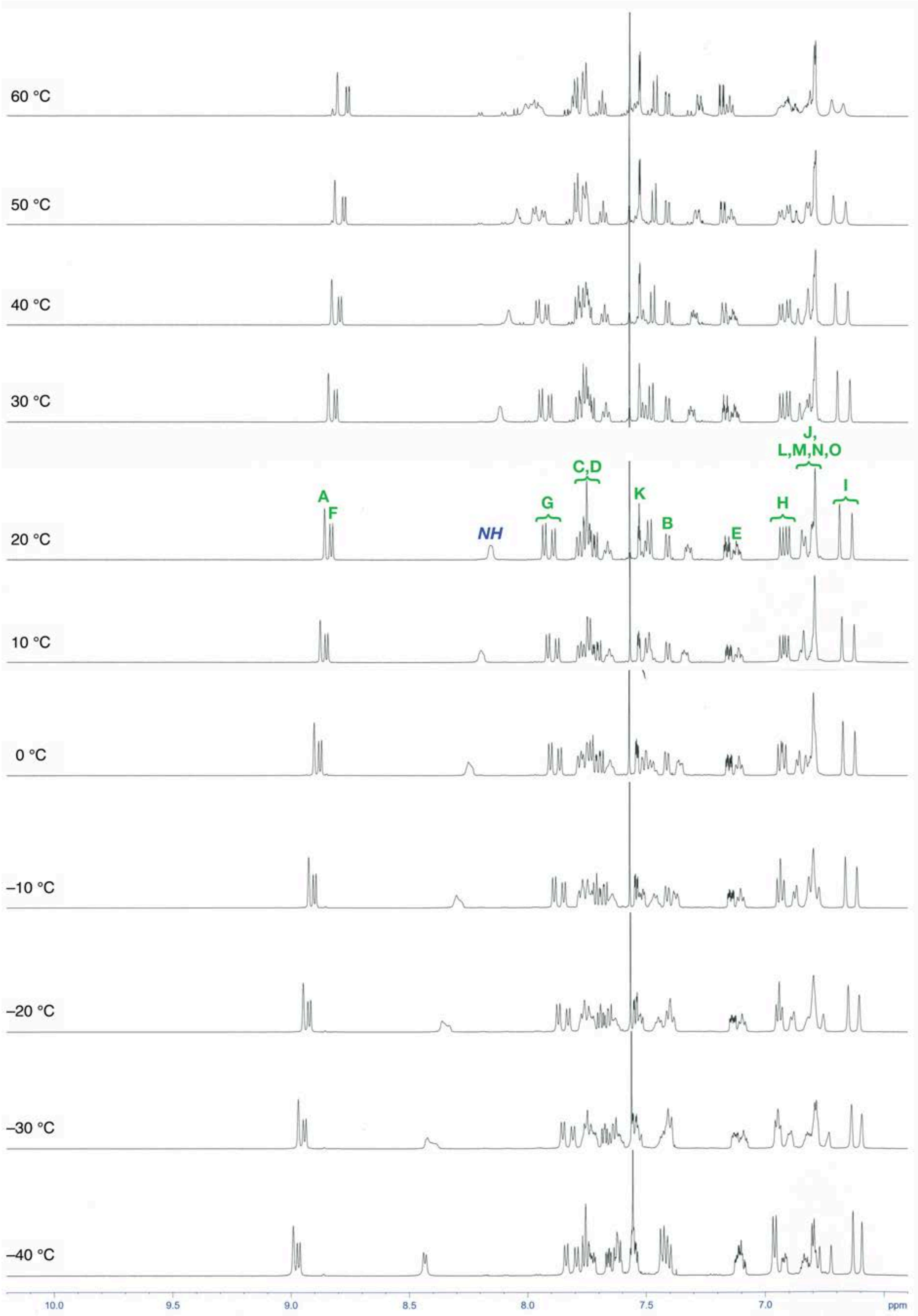

Supplementary Figure 53. VT-NMR spectrum (600 MHz) of **1a-OAc** in CD_3CN (10–6.5 ppm)

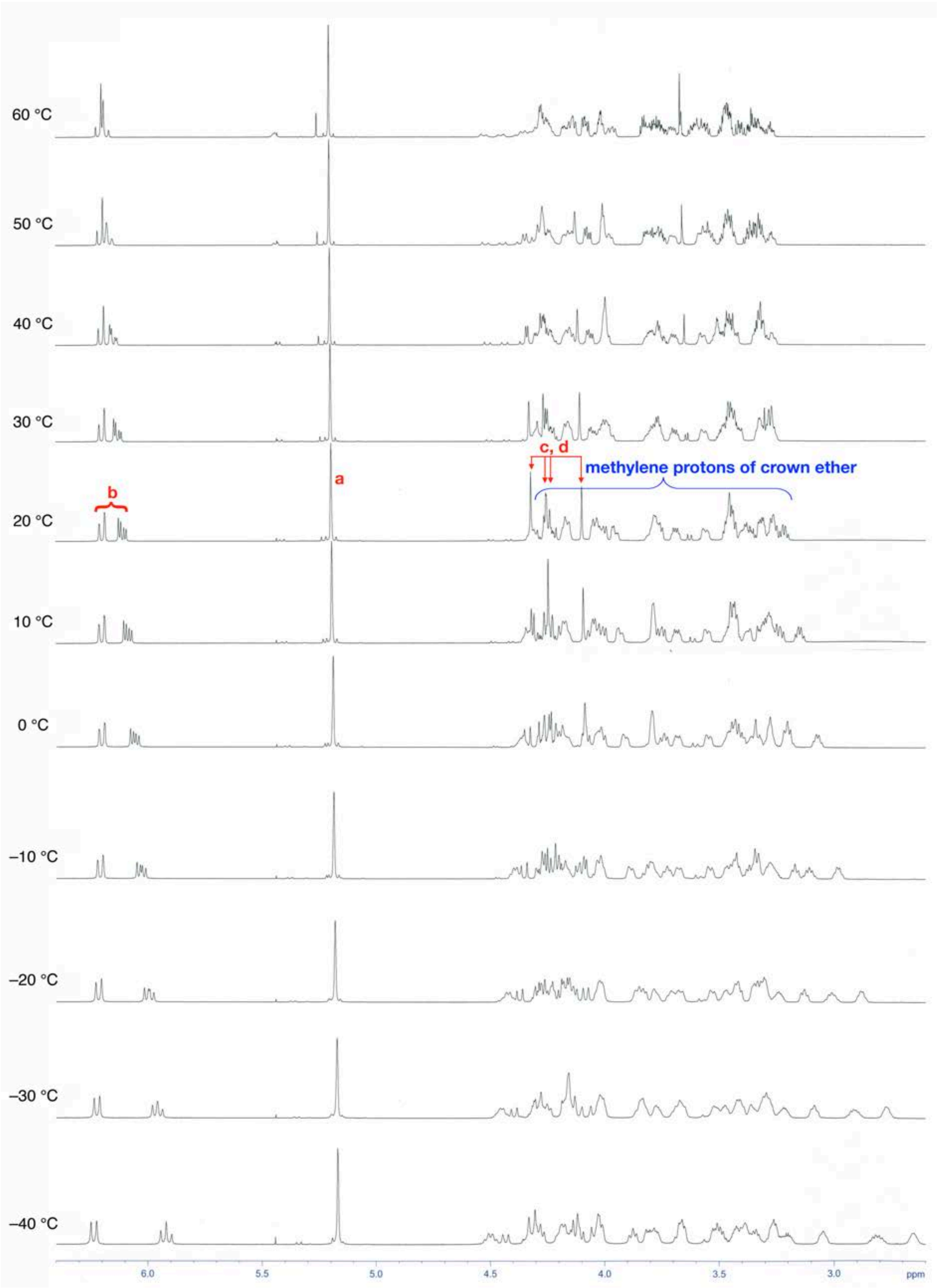

Supplementary Figure 54. VT-NMR spectrum (600 MHz) of **1a-OAc** in CD_3CN (6.5–2.5 ppm)

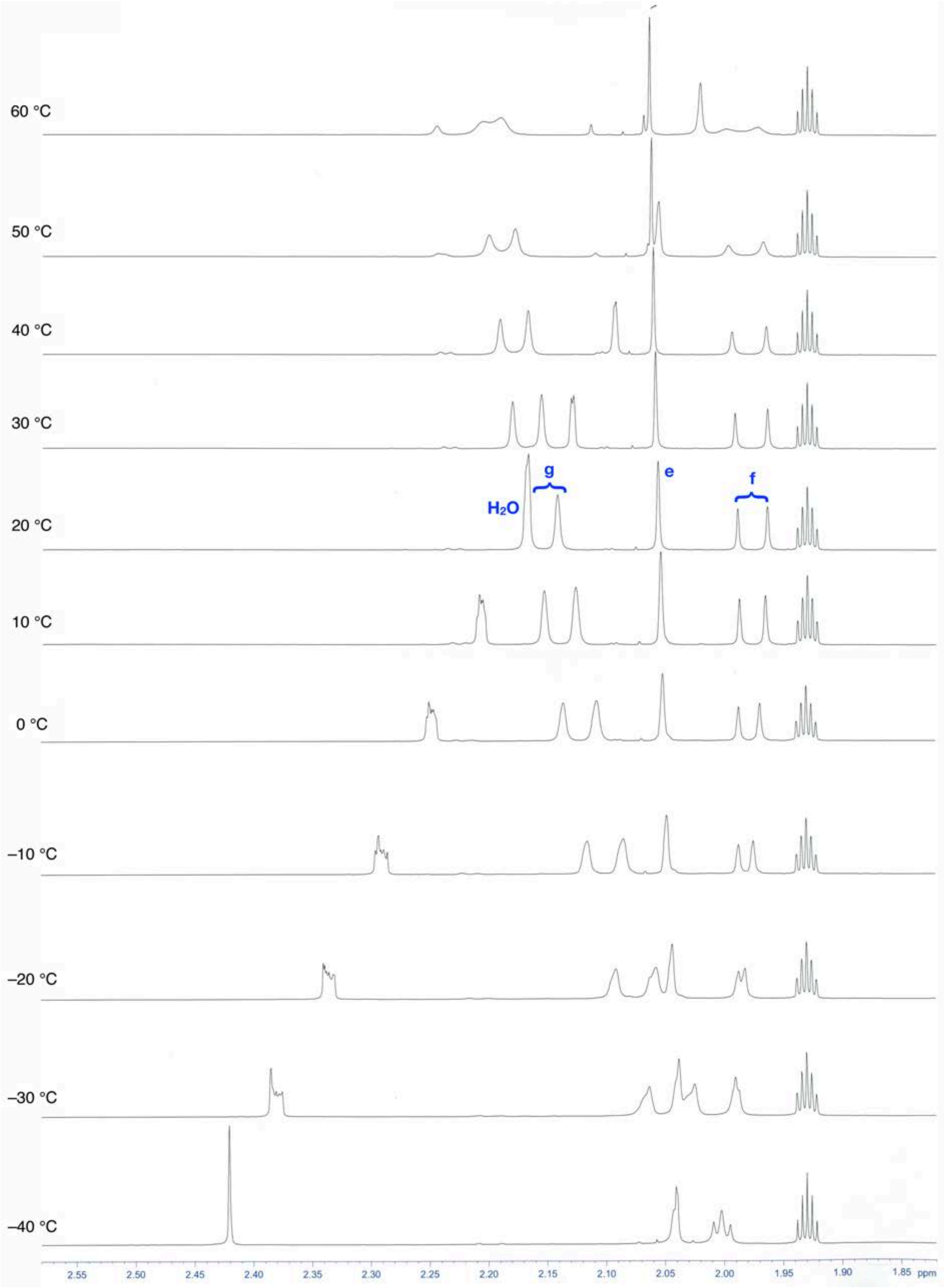

Supplementary Figure 55. VT-NMR spectrum (600 MHz) of **1a-OAc** in CD_3CN (2.5–1.9 ppm)

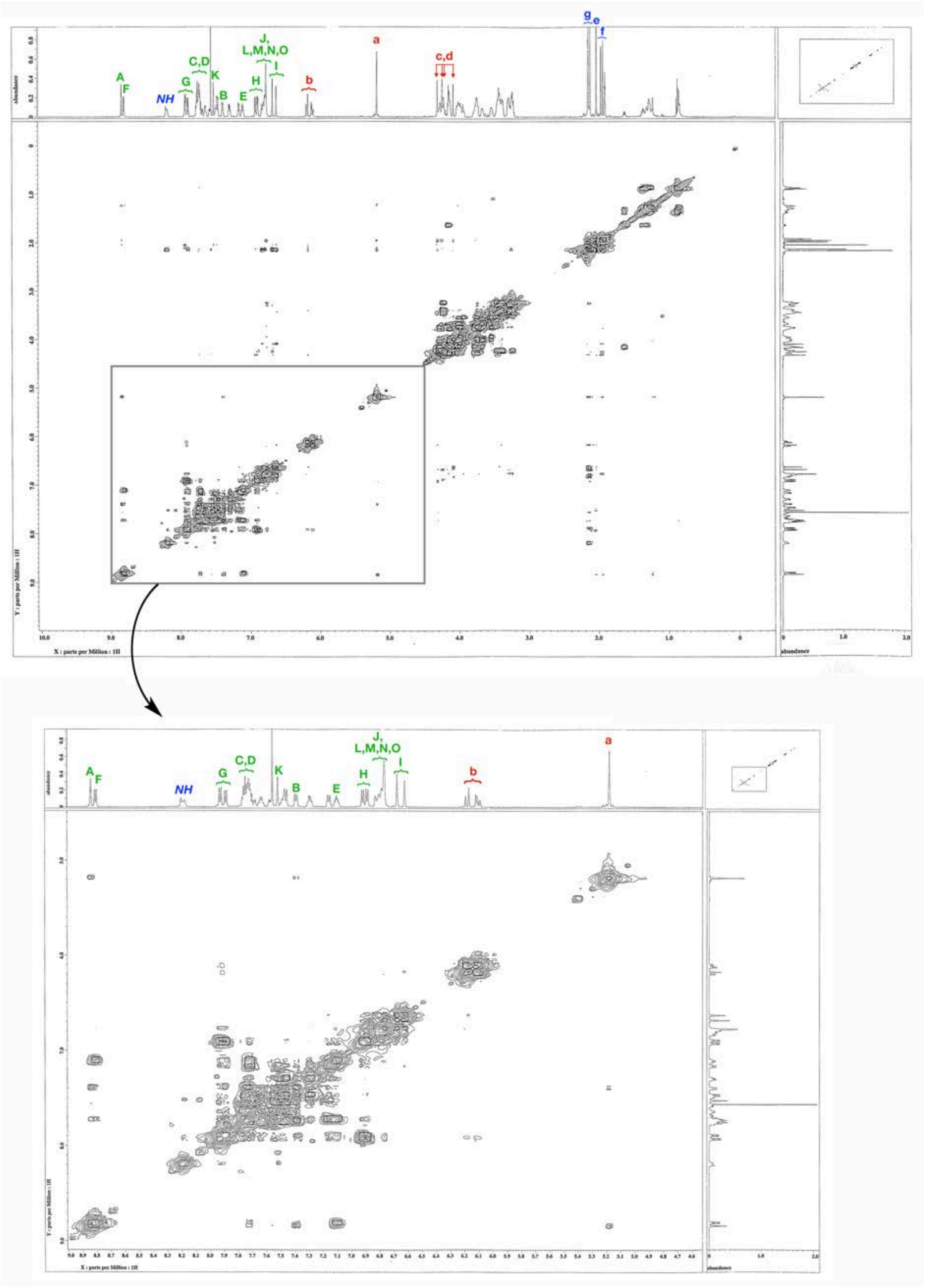

Supplementary Figure 56. COSY spectrum of **1a-OAc** in CD_3CN.

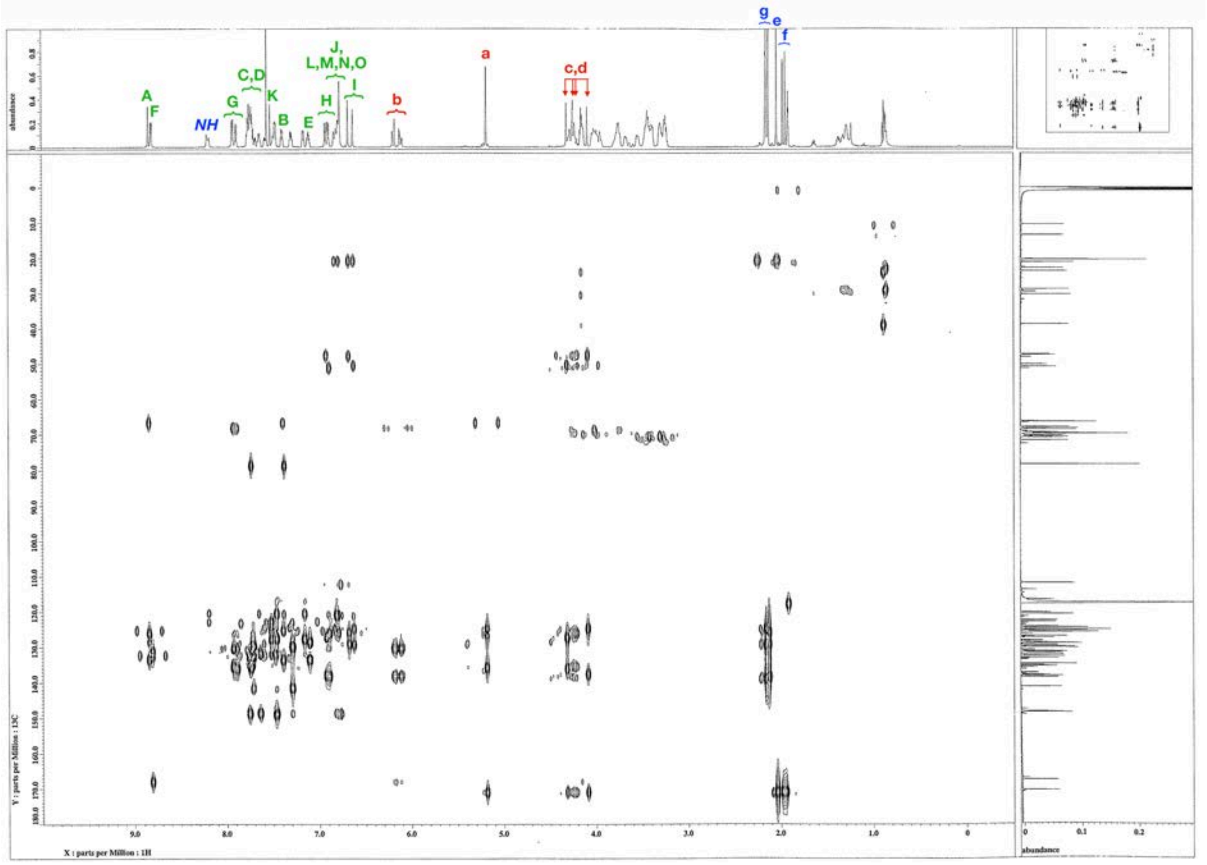

Supplementary Figure 57. HMBC spectrum of **1a-OAc** in CD_3CN.

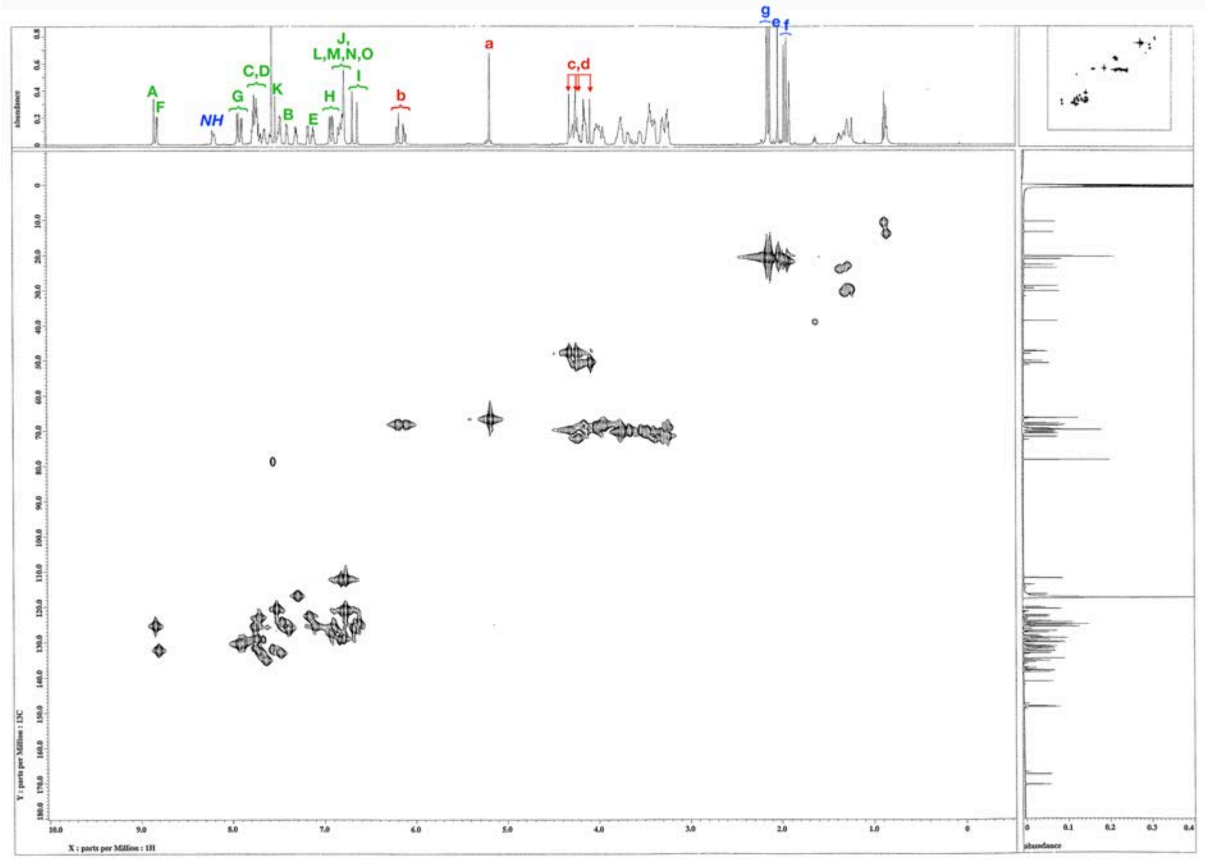

Supplementary Figure 58. HMQC spectrum of **1a-OAc** in CD_3CN.

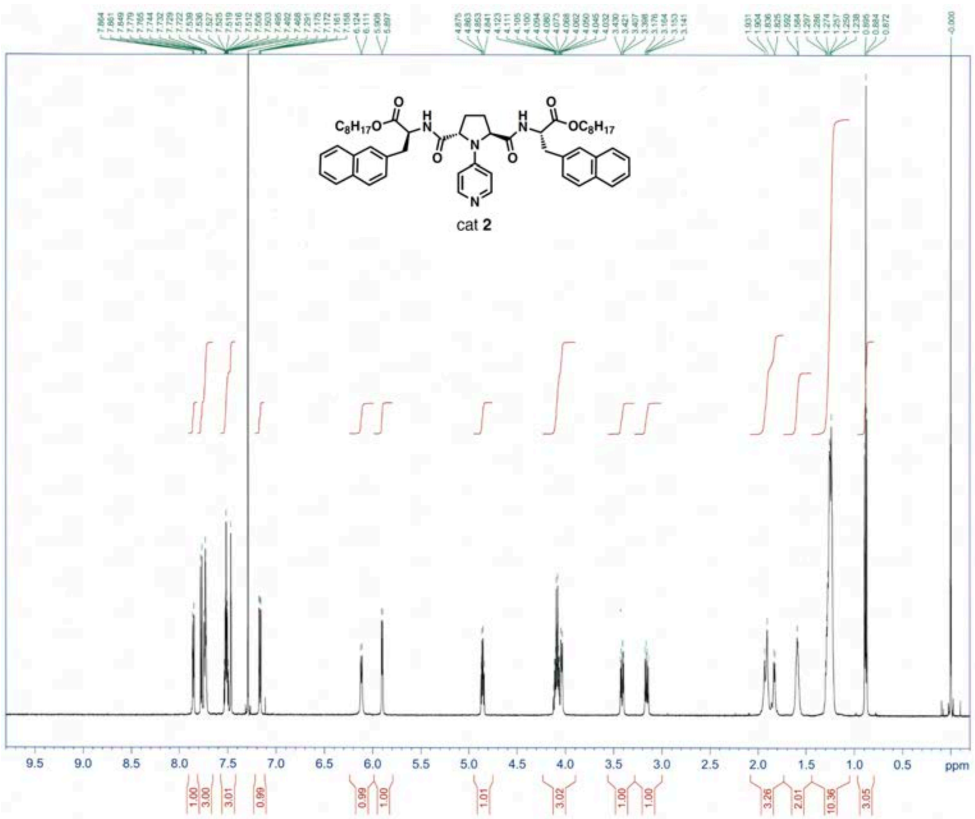

Supplementary Figure 59. ¹H-NMR of **2** in $CDCl_3$ at –40 °C.

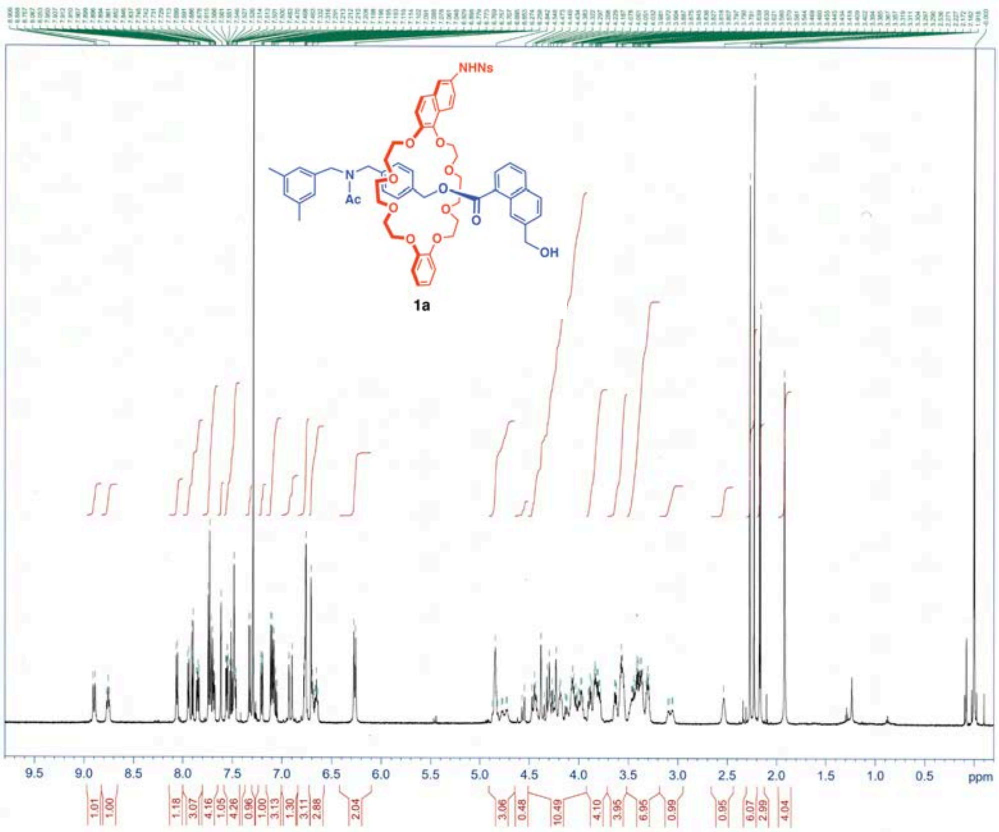

Supplementary Figure 60. ¹H-NMR of *racemic* **1a** in $CDCl_3$ at –40 °C.

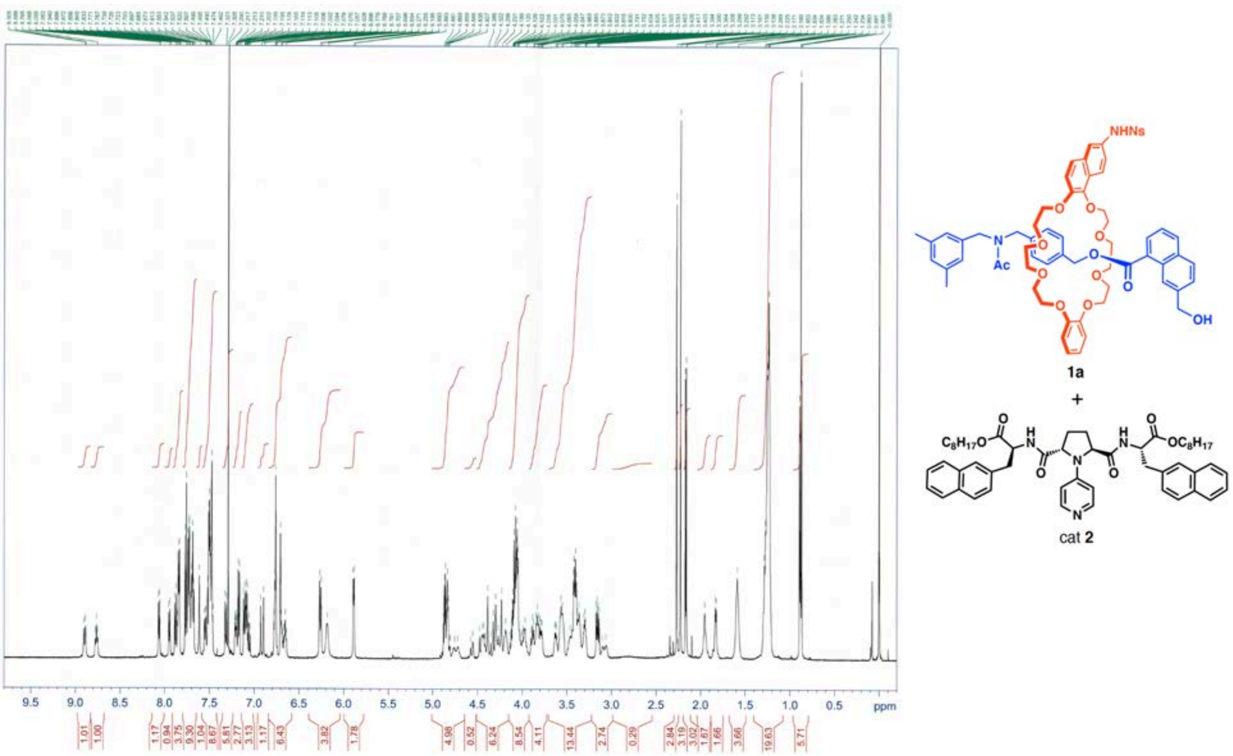

Supplementary Figure 61. ^{1}H-NMR of a 1:1 mixture of racemic **1a** and **2** in $CDCl_3$ (5 mM) at –40 °C.

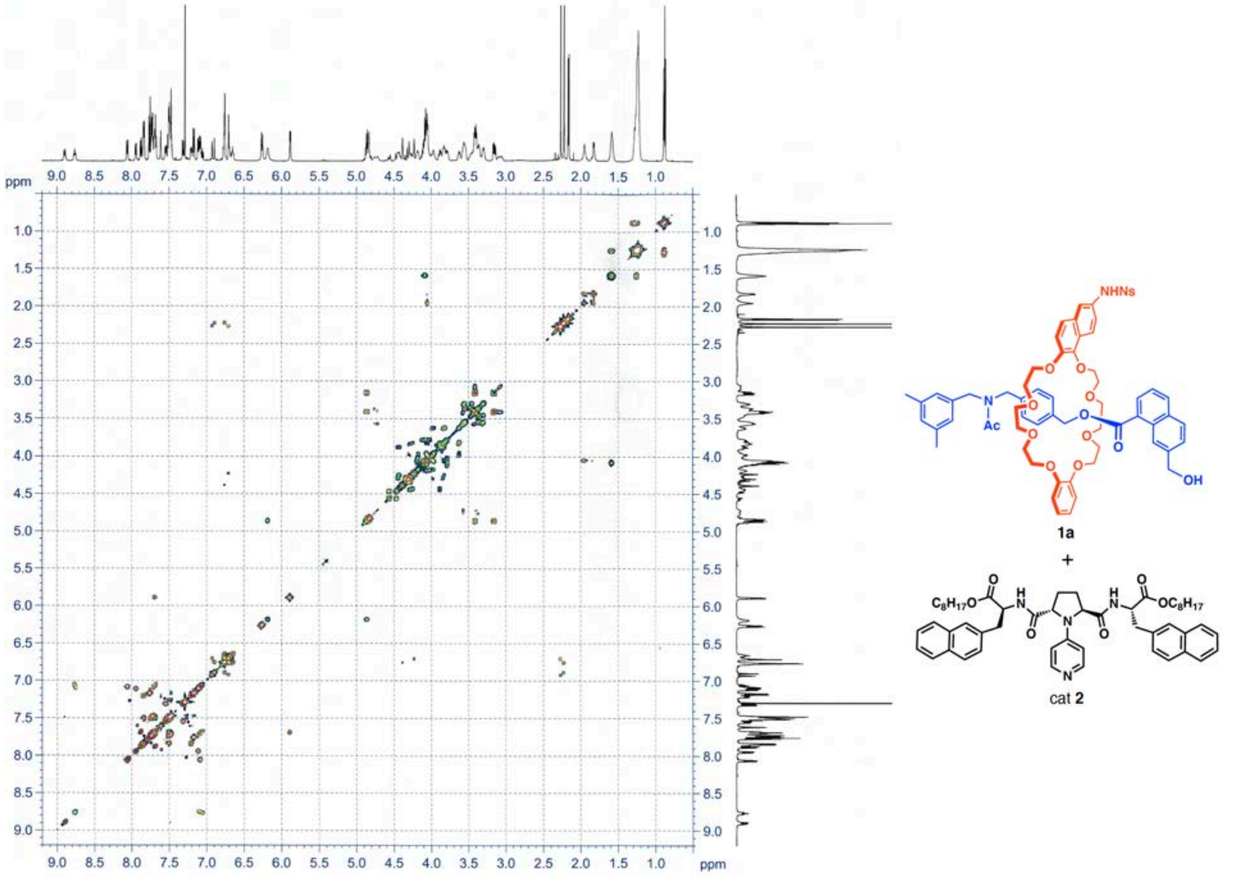

Supplementary Figure 62. COSY spectrum of a 1:1 mixture of racemic **1a** and **2** in $CDCl_3$ (5 mM) at –40 °C.

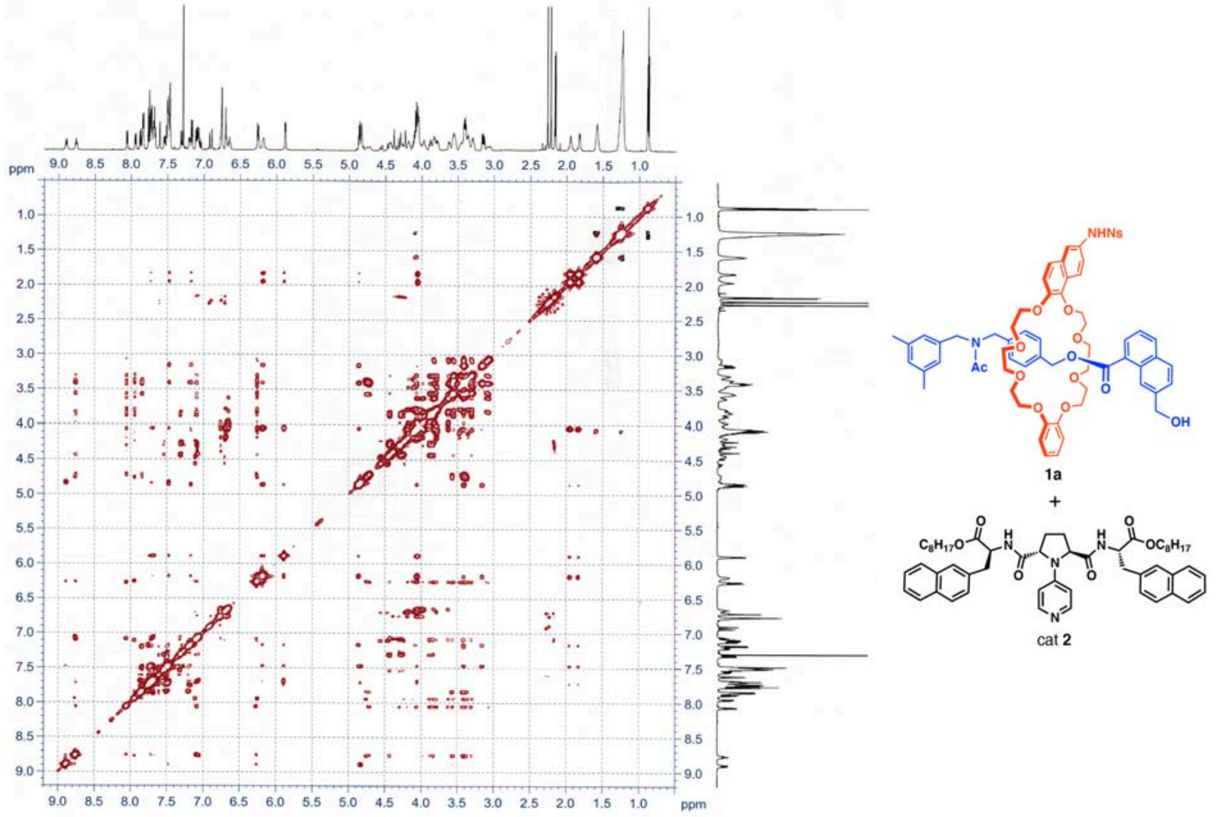

Supplementary Figure 63. NOESY spectrum of a 1:1 mixture of racemic **1a** and **2** in $CDCl_3$ (5 mM) at –40 °C.

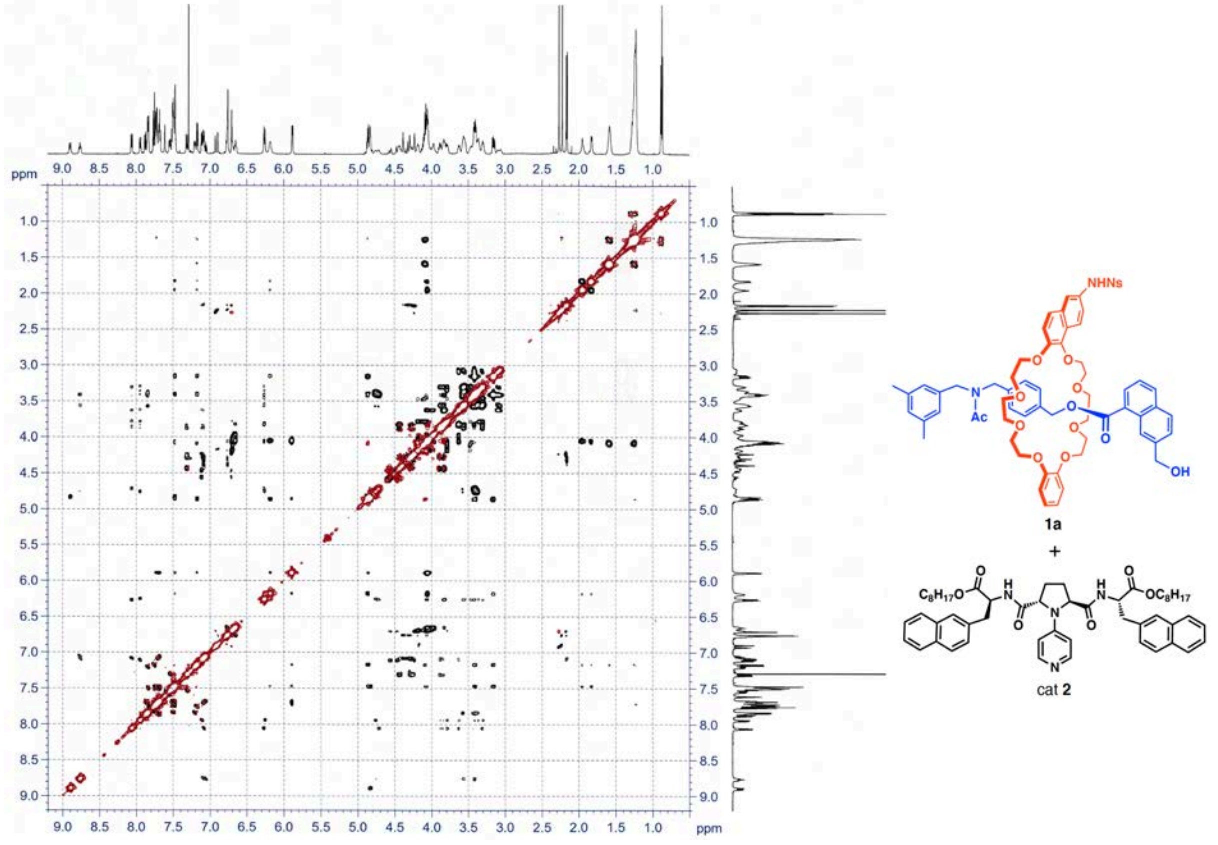

Supplementary Figure 64. ROESY spectrum of a 1:1 mixture of racemic **1a** and **2** in $CDCl_3$ (5 mM) at –40 °C.

Supplementary Reference

1. Kawabata, T., Muramatsu, W., Nishio, T., Shibata, T. & Schedel, H. A catalytic one-step process for the chemo- and regioselective acylation of monosaccharides. *J. Am. Chem. Soc.* **129**, 12890-12895 (2007).
